# A tumour-selective cascade activatable self-detained system for drug delivery and cancer imaging

**DOI:** 10.1038/s41467-019-12848-5

**Published:** 2019-10-24

**Authors:** Hong-Wei An, Li-Li Li, Yi Wang, Ziqi Wang, Dayong Hou, Yao-Xin Lin, Sheng-Lin Qiao, Man-Di Wang, Chao Yang, Yong Cong, Yang Ma, Xiao-Xiao Zhao, Qian Cai, Wen-Ting Chen, Chu-Qi Lu, Wanhai Xu, Hao Wang, Yuliang Zhao

**Affiliations:** 1 CAS Center for Excellence in Nanoscience, CAS Key Laboratory for Biomedical Effects of Nanomaterials and Nanosafety, National Center for Nanoscience and Technology (NCNST), No. 11 Beiyitiao, Zhongguancun, 100190 Beijing, China; 2 Institute of High Energy Physics, Chinese Academy of Sciences (CAS), Yuquan Road, 100049 Beijing, China; 3 Center of Materials Science and Optoelectronics Engineering, University of Chinese Academy of Sciences, No. 19A Yuquan Road, 100049 Beijing, China; 4 Department of Urology, The Fourth Hospital of Harbin Medical University, Heilongjiang Key Laboratory of Scientific Research in Urology, 150001 Harbin, China

**Keywords:** Cancer therapy, Drug delivery, Peptides

## Abstract

Achieving the activation of drugs within cellular systems may provide targeted therapies. Here we construct a tumour-selective cascade activatable self-detained system (TCASS) and incorporate imaging probes and therapeutics. We show in different mouse models that the TCASS system accumulates in solid tumours. The molecules show enhanced accumulation in tumour regions via the effect of recognition induced self-assembly. Analysis of the molecular penetration in tumour tissue shows that in vivo self-assembly increases the penetration capability compared to typical soft or hard nanomaterials. Importantly, the in vivo self-assembled molecules exhibit a comparable clearance pathway to that of small molecules, which are excreted from organs of the reticuloendothelial system (liver and kidney), while are relatively slowly eliminated from tumour tissues. Finally, this system, combined with the NIR probe, shows high specificity and sensitivity for detecting bladder cancer in isolated intact patient bladders.

## Abstract (web summary)

The activation of drugs within cellular systems may provide targeted therapies for cancer. Here, the authors make a drug delivery system that is activated within the cell and exploits XIAP expression to cleave a linker region, resulting in the self-assembly of the system and drug release within cancer cells.

## Introduction

Traditional molecular chemotherapeutics and imaging agents exhibit relatively low bioavailability due to their fast metabolic clearance^1,2^. Nanomaterial systems with targeting motifs and optimized particle sizes accumulate within the tumour and have enhanced molecular utilization efficiency^3–5^. Numerous strategies, such as developing small nano-sized superstructures^6^, enhancing long-term circulation^4^, and using physical tools, have been pursued to increase the accumulation and retention of nanoparticles^7,8^, decrease toxicity and optimize metabolism^9,10^. However, in the translation to clinical practice, the results remain unsatisfactory^11,12^. By taking advantage of small molecules and nanoscience, we expect in vivo self-assembly to outperform other strategies because (i) in addition to existing active/passive targeting processes, there is a new targeting mechanism which exhibit efficient accumulation and retention at desired sites; (ii) high penetration capability similar to that of small molecules in tumour region; and (iii) pharmacokinetics similar to that of small molecules except for reduced systemic toxicity in vivo. These advantages suggest that supramolecular systems may be promising for multifunctional nanomedicine.

Currently, in vivo self-assembly strategy based on supramolecular chemistry^13–16^ has been widely recognized as a promising tool for combating many life-threatening diseases^17–19^. Prominent examples in our previous work demonstrated some of the advantages of using peptide-based enzymatically induced assembly systems. For example, the aggregation/assembly induced retention (AIR) effect^20^ significantly enhanced bioactive molecule retention at disease sites, contributing to highly specific, sensitive and quantitative bioimaging^21^ and efficient tumour therapy^20,22^. Other groups have also confirmed that in vivo enzyme-triggered nanoassembly may facilitate endogenous enzyme imaging or induce cell death^23,24^. However, design of controllable building blocks with biofunctions and predicting the in vivo self-assembly behaviour still faces obstacles. Thus, de novo designed biocompatible small molecules with capability of self-assembly in living systems are imperative for in-depth biological studies. In this work, we attempt to construct a new tumour-selective cascade activatable self-detained system (TCASS) as a tumour nanomedicine, and reveal its mechanisms of efficient accumulation and retention at the tumour sites, and analyse the pharmacokinetics of the system.

**Fig. 1:**
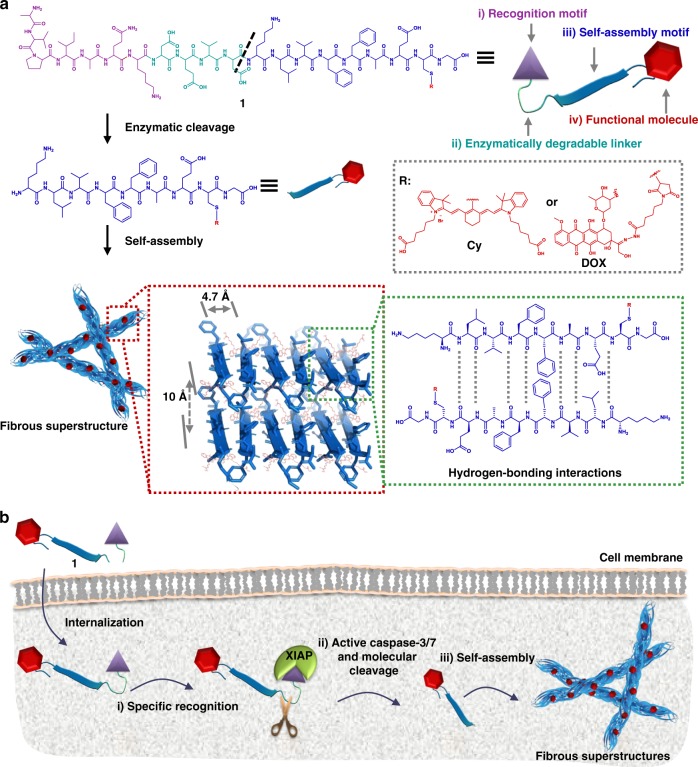
Illustration of the tumour-selective cascade activatable self-detained system (TCASS) and its molecular design. **a** the modularized peptide-based molecules consisted of four sections: (i) recognition motif: AVPIAQK was the binding motif; (ii) DEVD was the enzymatically degradable linker; (iii) KLVFFAECG was the self-assembly motif; and (iv) cyanine dye (Cy) or doxorubicin (DOX) was functional molecule. **b** the mechanism of the specific recognition, molecular cleavage and in situ self-assembly of molecule 1. First, molecule 1 was specifically recognized by X-linked inhibitor of apoptosis protein (XIAP); then the caspase-3/7, activated by the recognition process, cleaved the molecules; further, the cleaved molecules rapidly self-assembled in situ and formed fibrous β-sheet superstructures; finally, the β-sheet nanostructures significantly enhanced the accumulation and retention of functional molecule in tumour tissue

Herein, we rationally design a TCASS system to optimize accumulation, penetration and organ competition. The modularized units of the system (Fig. 1a) consist of (i) a tumour-specific recognition motif (AVPIAQK), (ii) an enzymatically cleavable linker (DEVD), (iii) a self-assembly motif (KLVFFAECG) and (iv) a functional molecule (cyanine dye, Cy; or doxorubicin, DOX). The recognition motif specifically recognizes the X-linked inhibitor of apoptosis protein (XIAP), which is overexpressed in cancer cells^25–28^. Then the recognition process activates downstream caspase-3/7, which triggers the self-assembly process (Fig. 1b). Hydrogen bonding directs the growth of the assemblies, and we obtain fibrous superstructures with β-sheet domains. Our system is retained in cancerous cells based on nucleation-elongation mechanisms^29,30^. Due to the AIR effect^20^, the accumulation efficiency of the system is approximately 9.2 ± 0.5% ID/g via IV injection after 48 h, which is higher than that of small molecules (<3.0% ID/g)^1,31^ and nanomaterials (5.2 ± 4.4% ID/g)^32^ reported in mouse models. Compared to typical nanomaterials (e.g. SiO_2_ nanoparticles and liposomes with diameters of approximately 100 nm), the TCASS exhibits remarkable penetration due to its monomeric molecular diffusion before self-assembly. Meanwhile, the organ competition behaviour of the TCASS is comparable to that of small molecules, which are rapidly excreted from the liver and kidneys. By integration of the chemodrug or contrast agent, the TCASS can be successfully employed in the process of drug delivery in mice or image-guided surgery in isolated patient intact bladders.

## Results

### Self-assembly behaviour of functional peptides in the solution

**Fig. 2:**
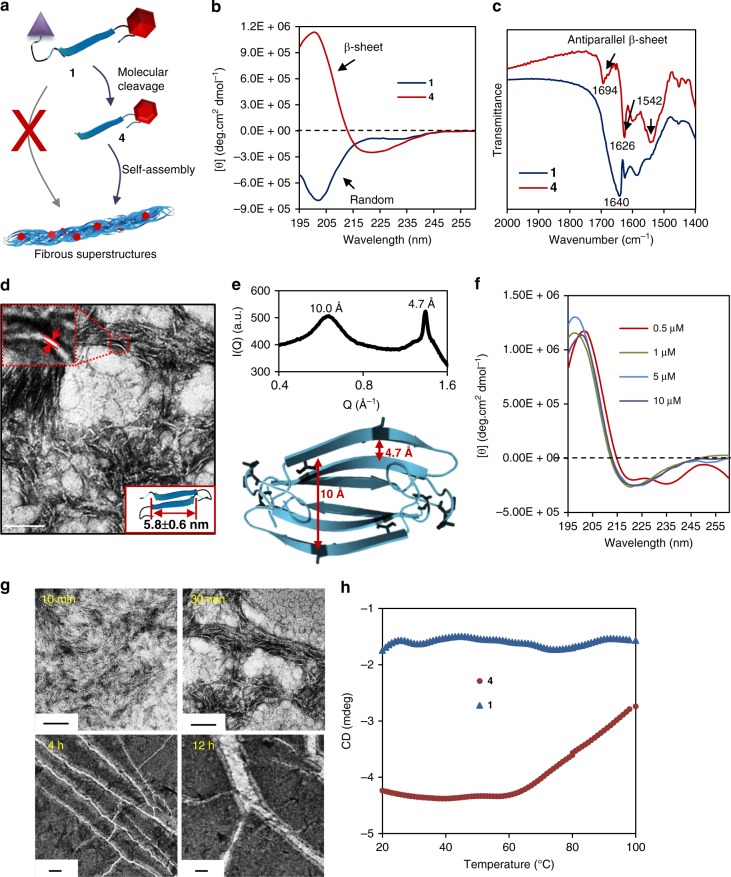
The structure conformations and self-assembly properties of molecule 1 in aqueous solution. **a** Illustration of the self-assembly strategy of molecule 1. **b** CD spectra of molecule **1** and molecule 4 in deionized water at a concentration of 1 μM. **c** FTIR spectra of molecule 1 in a monomeric state and molecule 4 in a fibrous assembled state. The arrows identify the typical characteristic peaks of the antiparallel β-sheet structure. **d** TEM image of self-assembled β-sheet nanofibrils of molecule 1 after treatment with caspase-3 (substrate-to-enzyme ratio of 1 μM/U) in HEPES buffer (50 mM, pH 7.4) for 2 h. The manual measurement of the nanofibril diameter is shown in the zoomed image, and the computational simulation result for the length of molecule 4 is inserted. **e** WAXS and computational simulation results of the β-sheet self-assemblies (molecule 4) identified the β-sheet and laminate spacing of the structures. **f** The concentration-dependent CD spectra of molecule 4 in the concentration range of 0.5–10 μM. **g** Time-dependent TEM images of molecule 4 from 10 min to 12 h for monitoring the growth process of the nanofibrous structures. **h** Temperature-dependent CD intensity changes at 220 nm of molecules 1 and 4 with a heating rate of 1 °C/min and an equilibrium period of 30 s to reveal the self-assembly mechanisms. Scale bar: 100 nm

To systematically investigate the structure-related self-assembly behaviour, we synthesized three different peptide molecules, i.e.: molecules 1 (AVPIAQKDEVDKLVFFAEC(Cy)G) with recognition and self-assembled motifs, molecule 2 (MDEKAQKDEVDKLVFFAEC(Cy)G) with the self-assembled motif alone and molecule 3 (AVPIAQKDEVDEC(Cy)G) with the recognition motif alone (Supplementary Figs. 1–6). In order to further elucidate the functions of the designed motifs and the self-assembled nanostructures, we synthesized a control molecule 4 (KLVFFAEC(Cy)G) (Supplementary Figs. 7 and 8) that mimics the cleaved residues from molecule 1 (Fig. 2a). First, we tested the enzymatic response of these molecules by high-performance liquid chromatography (HPLC). The results indicated that the DEVD motif was hydrolysed in the presence of recombinant human caspase-3 with a substrate-to-enzyme ratio of 1 μM/U in HEPES buffer (Supplementary Fig. 9). Next, the secondary structures of the self-assembled peptides were studied by circular dichroism (CD) spectrometry. The full-length molecule 1 (1 μM) exhibited a negative Cotton effect at 199 nm, which was attributed to the random coiled conformation^33,34^. The CD signals of molecule 4 showed a positive value at 199 nm and a negative value at 217 nm, which were considered to be β-sheet stacking self-assembly^35^ (Fig. 2b). Moreover, similar CD signals of β-sheet were observed when molecule 1 was incubated with caspase-3 for 2 h in HEPES buffer (Supplementary Fig. 10), indicating that after removing the responsive motif (Supplementary Fig. 9), the simultaneously released residues would self-assemble into nanofibrils through hydrogen bonding interactions^36^. To study the hydrogen bond-assisted self-assembly and its conformation, we performed Fourier transform infrared analysis of molecules 1 and 4. As shown in Fig. 2c, the amide II band was detected at 1542 cm^−1^, the amide I band shifted from 1640 to 1626 cm^−1^ and higher energy but weaker shoulder band was at 1694 cm^−1^ suggesting the formation of nanofibrils with an antiparallel β-sheet conformation, which was similar to the molecule 4 and assembled Aβ (16–22)^37^. Furthermore, to study the blocking effect of Cy molecules in the peptide sequence, we synthesized two control molecules with high structural similarity to molecule 1, P1 (AVPIAQKDEVDKLVFFAECG) and 5 (AVPIAQKDEVDGC(Cy)KLVFFAEG) (Supplementary Figs. 1, 11 and 12). Unlike the well-dissolved molecule 1 in water, both P1 and 5 molecules simultaneously formed fibrous structures (Supplementary Fig. 13) under the same conditions, indicating that the Cy molecule and its modified site in the peptide sequence played a crucial role in the self-assembly behaviour. Nanofibrils of molecule 1 with a diameter of approximately 5.8 ± 0.6 nm were visually observed after incubation with caspase-3 for 2 h in HEPES buffer (Fig. 2d, Supplementary Fig. 14), which corresponded well to the molecular length of molecule 4 (Fig. 2d). The similar fibrous structures were also observed in TEM images for molecule 2 (Supplementary Fig. 15) but not for molecule 3, which lacked the self-assembly motif. These results demonstrated that caspase-3-mediated proteolytic hydrolysis induced the self-assembly of molecules 1 and 2, which then formed nanofibrils. The molecular packing mode was further studied by wide-angle X-ray scattering (WAXS). We observed a strong and sharp reflection at 4.7 Å and a broad band at 10 Å (Fig. 2e), which was attributed to the spacing of the β*-*sheets and laminates, respectively^37,38^. Our results are consistent with the characteristic amyloid cross-β diffraction pattern^37^. Compared to the monomeric molecule 1, the photostability of Cy in the assembled molecule 4 was significantly enhanced. After irradiation for 6 h, molecule 1 showed 42% photobleaching in pure water, while only 5% photobleaching happened for molecule 4 under the same condition (Supplementary Fig. 16). The rate constants of the photodegradation reaction of molecules 1 and 4 were tested in parallel. The photodegradation rate of molecule 1 was 7.5-fold larger than that of 4. Based on the results above, we concluded that the β-sheet structure-enriched nanofibril effectively bounded and locked the Cy dye in hydrophobic domains and consequently inhibited the radiationless internal conversion (IC), isomerization and degradation of Cy^39^. We analysed the self-assembly process and mechanism of the peptide molecules using concentration/temperature/time-dependent CD spectra and time-dependent TEM technique. As shown by the concentration/time-dependent CD spectra of molecule 4 in water, obviously self-assembled β-sheet structures were detected when the concentration was more than 1 μM (Fig. 2f). The formation of the β-sheet structure was a kinetically controlled process on the order of minutes, and the growth lasted for a period of hours (Supplementary Fig. 17). Accordingly, the size and morphological evolution of the resulting nanofibrils were studied by TEM over an ageing period from 10 min to 12 h (Fig. 2g). The large diameter of the nanofibrils indicated bundles of infinitely long nanofibrils. Meanwhile, a sigmoidal curve was exhibited by the temperature-dependent CD spectral change at 220 nm, implying nucleation-dependent growth (Fig. 2h). The curve indicated that the mechanism was similar to the well-known nucleation-elongation mechanisms of β-amyloid (Aβ) peptides^40^.

### The mechanism of the TCASS in living cells

**Fig. 3:**
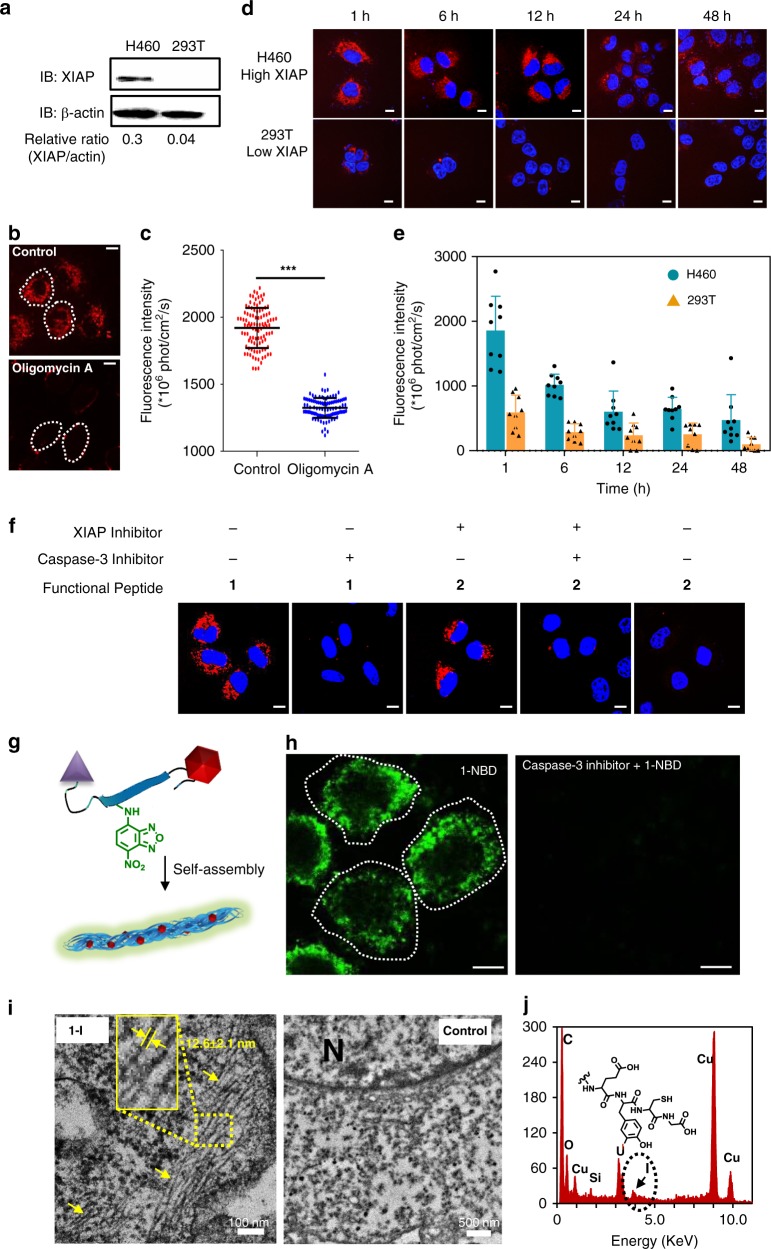
Recognition and self-assembly of molecule 1 in cells. **a** Western blots of XIAP expression in H460 and 293T cells. **b** Confocal images of H460 cells treated with or without energy-dependent inhibitor (oligomycin A, 20 μM) for 1 h, and followed by washing with PBS and treated with the molecule 1 (50 μM) for 1 h. **c** Statistical analysis of average fluorescence intensities in the cytoplasm of H460 cells from confocal images. Data are presented as the mean ± s.d. (*n* = 100). ***p < 0.001, *p* values were performed with one-way ANOVA followed by post hoc Tukey’s test for the indicated comparison. **d** Confocal images of time-dependent monitoring of H460 and 293T cells treated with the molecule 1 (50 μM) for 1 h followed by washing with PBS and replacing the medium and a further incubation up to 48 h. **e** Statistical analysis of average fluorescence intensities in the cytoplasm of H460 and 293T cells from confocal images with a timescale range of 1–48 h. Data are presented as the mean ± s.d. (*n* = 15). **f** H460 cells pretreated with P6 and z-VDK-FMK pretreated for 3 h, respectively, for the artificial adjustment of XIAP expression and caspase-3 activity. Then remove the supernatant and wash it with PBS. Further, molecule 1 or 2 was incubated with treated-H460 for 1 h, followed by washing with PBS and fresh medium replacement. Finally, incubate up to 6 h and imaging by confocal microscope. **g** Schematic diagram illustrates the process of NBD fluorescence illumination. **h** Confocal images of H460 cells treatment with molecule 1-NBD (50 μM) for 1 h. **i** TEM images of ultrathin section of H460 cells treated with molecule I-1 (50 μM) and without the molecule I-1 for up to 12 h. N labels the nucleus of the H460 cell, and the red arrows and dashed box indicate the nanofibrils formed from the molecule 1. **j** EDS scanning of the nanofibrils with iodine labelling in the cells. Scale bar: 10 μm

High levels of XIAP^41^ may be a potential tumour biomarker^42^. We selected H460 (human NSCLC cells, high XIAP) and 293T (normal human kidney cells, low XIAP) for in vitro studies to evaluate the targeting mechanism. First, we examined the expression of XIAP in two cell lines by western blotting. As shown in Fig. 3a, compared to the XIAP level in 293T cells, XIAP was overexpressed in H460 (Fig. 3a, Supplementary Fig. 18), which was consistent with the literature^28^. Then we found that the internalization of molecule 1 into cells was energy-dependent because the fluorescence intensity was decreased when the cells co-treated with energy inhibitor (oligomycin A)^43^ (Fig. 3b, c). As widely reported, endocytosis is one of the most important energy-dependent ways for cells to obtain macromolecules from the extracellular space^44,45^. We therefore investigated whether molecule 1 enters the cells through the endocytic pathway. The endocytic pathway includes macropinocytosis, clathrin-mediated endocytosis and caveolin-mediated endocytosis, which can be inhibited by amiloride, chlorpromazine and filipin III, respectively^45,46^. Consequently, we detected the fluorescence signals in cells after pre-treatment with these inhibitors. As a result, the intracellular fluorescence signals were significantly decreased only when filipin III was added (Supplementary Fig. 19). Therefore, we could speculate that molecule 1 was likely to enter the cells via a caveolin-mediated endocytic pathway. According to a previous report, the AVPIAQK peptide sequence was essential for binding XIAP and blocking its caspase-inhibitory activity^47^. Therefore, we treated both types of cells with molecule P6 (AVPIAQKGGGRRRRRRRRGC) (Supplementary Fig. 20), which could interact with XIAP and subsequently activate caspase-3 in H460 cells but had little effect on 293T cells (Supplementary Fig. 21)^28^. Surprisingly, the H460 cells survived despite the activation of caspase-3 after treatment with molecule 1 and molecule 3. We carefully repeated the experiments and studied the apoptotic cell death induced by molecules 1, 2 and 3 by flow cytometry (Supplementary Fig. 22) and cell viability assays (Supplementary Fig. 23). No apoptosis was observed from any of the experimental groups, this was probably because the cells could tolerate a wide range of caspase-3 activation levels^48^.

To study the XIAP-specific AIR effect of molecule 1 inside cells, we incubated molecule 1 with H460 for 1 h then washed with phosphate-buffered saline (PBS) and replaced the medium. After further incubation for 48 h, we still observed significant red fluorescence of molecule 1 in the cytoplasm by confocal microscopy. Under the same experimental protocol, only weak fluorescence was found in molecule 1-treated 293T cells (Fig. 3d). Statistical analysis of the average fluorescence intensity in the cytoplasm showed that the molecule 1 exhibited higher accumulation and longer retention in H460 cells (up to 48 h) than that in 293T cells. While the maximum difference was observed at 6 h, and the fluorescent intensity in H460 cells was fivefold higher than that in 293T cells (Fig. 3e). These results were further confirmed by cell passage after the cell uptake molecules. In Supplementary Fig. 24, it was shown that the labelling ratio of H460 cells was 100% at the first generation and remained above 16.4% at the third generation as compared to the untreated cells. On the contrary, only 6.83% and 3.89% of molecule 2- and 3-treated cells were labelled at the third generation, respectively. These results implied that the targeting mechanism of the TCASS contributed to the accumulation and retention of molecule 1 in XIAP-overexpressing cells. Furthermore, to rule out the possibility that the differences in molecular uptake originated from intrinsic properties of the different cell lines rather than depending on both recognition and post-cleavage self-assembly, H460 cells were treated with molecules 1 and 2 independently in the presence of inhibitors of XIAP or caspase-3 (Fig. 3f). When caspase-3 activity was inhibited by z-VDK-FMK^28^, the fluorescence intensity of H460 cells treated with molecule 1 was significantly decreased compared with the untreated group. Additionally, down-regulating XIAP with P6 (ref. ^49^) in H460 cells and blocking the caspase-3 activity with z-VDK-FMK dramatically decreased the fluorescence intensity of molecule 2. All of the above results indicated that the activity of caspase-3 was the indispensable factor for molecule self-assembly. We speculate that the recognition of XIAP promotes the activity of caspase-3, which consequently cleaves molecule 1 and results in self-assembly. To study the supramolecular nanofibrils formation inside cells, we synthesized molecule 1 labelled 4-nitro-2,1,3-benzoxadiazole (NBD) (1-NBD) (Supplementary Figs. 25 and 26). Recently, Xu and co-workers^23^and Ryu and co-workers^50^ reported that NBD, which is brightly fluorescence in hydrophobic environments, could act as a reporter for nanofibrils formation with small peptide molecule intracellular^23,50^. Bright green fluorescence was visible in H460 cell, negligible fluorescence for the control group treated with caspase-3 inhibitor (Fig. 3g, h). To obtain visual evidence of supramolecular nanofibrils inside cells, we utilized high-resolution bio-TEM and energy dispersive spectroscopy (EDS) to study morphology and identify the elemental distribution of the nanofibrils in ultrathin sections of cells (Fig. 3i, j). To differentiate sample signals from the background, an iodine-labelled molecule 1 (I-1) (AVPIAQKDEVDKLVFFAEY(I)C(Cy)G) was synthesized (Supplementary Fig. 27). Similar assemblies between 1-I and 1 suggest there was no influence to molecular self-assembly by this labelling method (Supplementary Fig. 28). We then used bio-TEM to observe the formation of nanofibrils inside H460 cells after treatment with I-1 (50 μM) for 12 h. Fibrous structures were clearly observed in treated H460 cells (Fig. 3i, Supplementary Fig. 29). Meanwhile, the EDS results unambiguously showed that the significantly heightened iodine in the elemental distribution of these nanofibrils was attributable to I-1 (Fig. 3j). However, similar fibrous morphology was barely observed in untreated H460 cells and 293T cells (Supplementary Figs. 29 and 30). Consequently, the TCASS system contributes to the accumulation and long-term retention of molecules in tumour cells.

### The TCASS enhanced accumulation in the tumour model

**Fig. 4:**
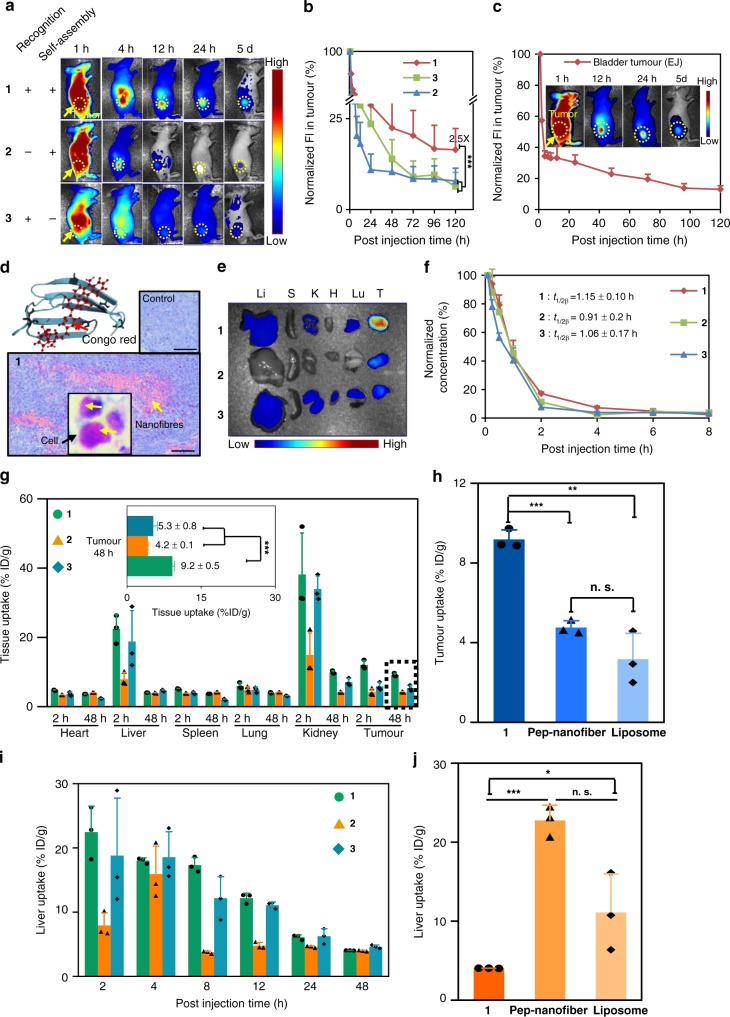
The TCASS induced accumulation and organ competition. **a** Representative NIR fluorescence images of molecules 1, 2 and 3 on H460 tumour-bearing mice after IV injection. Images were acquired at 1, 4, 12, 24 h and 5 days post injection (p.i.). **b** Normalized fluorescence intensity in tumour in **a**. **c** Representative NIR fluorescence images and normalized fluorescence intensity in tumour on EJ tumour-bearing mice after IV injection. **d** Light microscopy images of Congo red-stained sections of the tumours. The yellow arrow indicated the nanofibers. From the magnified image, it could be observed that the stained area was in the cell. **e** Ex vivo NIR fluorescence images of tumour and major organs collected post 48 h injection. Li liver, S spleen, K kidney, H heart, Lu lung, T tumour. **f** Blood circulation data on molecules 1, 2 and 3. Blood was collected from the mouse tail at different points of time p.i. The molecule levels (in units of ID%/g) in the blood were measured by fluorescence spectroscopy. **g** The molecular uptakes of molecules 1, 2 and 3 by tumour and major organs in H460 tumour-bearing nude mice at 2 and 48 h p.i. The molecular content of molecules 1, 2 and 3 was determined by the fluorescence of diluted tissue lysates. **h** Statistical analysis of the tumour accumulation of molecule 1, Pep-nanofiber and Liposome at 48 h p.i. **i** Liver uptake of molecules 1, 2 and 3 in H460 tumour-bearing mice at 2, 4, 8, 12, 24 and 48 h after injection. **j** Statistical analysis of the liver retention of molecule 1, Pep-nanofiber and Liposome at 48 h p.i. All data are presented as the mean ± s.d. (*n* = 3). The molecular concentration used in the experiment was 14 mg/kg.%ID/g = percentage of the injected dose per gram of tissue. In **b**, **g**, **h** and **j**, n.s. not significant, ***p < 0.001, **p < 0.01, *p < 0.05, *p* values were performed with one-way ANOVA followed by post hoc Tukey’s test for the indicated comparison. Scale bar: 100 μm

We evaluated the TCASS in a mouse model (BALB/c nude) with xenografted XIAP-overexpressing H460 and EJ cells. Time-dependent fluorescence images of mice treated with molecules 1, 2 and 3 (14 mg/kg) were recorded at 1 h post injection and thereafter up to 5 days using a multispectral imaging system. Over time, during the clearance of the molecules, the stranded fluorescence signal in the tumour site of the mice treated with molecule 1 was clearly differentiable from the surrounding normal tissues as early as 4 h post injection. After 12 h, only the tumour continued to have a relatively strong fluorescence signal, which was stable and lasted up to 5 days (Fig. 4a). In contrast, in the control groups of mice treated with molecules 2 and 3, a major portion of these molecules were quickly excreted within 4 h (Fig. 4a). To obtain quantitative data on the molecules retention in the tumour sites, we evaluated the integrated fluorescence intensity in the region of tumour site shown in Fig. 4a and Supplementary Fig. 31. With the similar fluorescent signal intensity of these three molecules at 1 h, the collected molecule 1 exhibited approximately 22.0% retention efficiency repeated three times up to 48 h post injection, while less than 10.0% retention efficiency was observed for molecules 2 and 3 (Fig. 4b, Supplementary Fig. 32). Surprisingly, on the fifth day, the tumour treated with molecule 1 still retained over 16.0% of its fluorescence signal, which was nearly 2.5-fold higher than the value for tumours treated with molecules 2 and 3. After quantitative analysis, there was still a very low fluorescence signal around the tumour site after 5 days. In our previous work, we demonstrated that the self-assembled nanofibrils were excreted out of the tumour by hydrolysis^5^. Therefore, we speculated that the signal mainly came from the metabolite of the nanofibrils in tumour. These results implied that the molecular recognition and subsequent caspase-3 cleavage allowing self-assembly were both indispensable for tumour-specific targeting. To validate the generality of the TCASS in different tumours in vivo, besides the H460 lung xenografts mouse model, we carried out additional experiments in EJ bladder xenograft tumour model and evaluated the targeting, accumulation and retention (Fig. 4c). EJ cells showed overexpression of XIAP as well as H460 cells, compared to 293T cells (Supplementary Fig. 33). The results of targeting, accumulation and retention of molecule 1 in the EJ bladder xenograft tumour mouse were the same with the H460 lung xenografts mouse model. Based on these results, we concluded that the TCASS showed good application to tumours with high expression of XIAP. Furthermore, to clearly visualize and identify the fibrous superstructures of molecule 1 in the tissue, we used Congo red, which is a gold standard amyloid dye for clinical histological assays^51^, to stain paraffin sections of tumour tissues. The self-assembled fibrous structures in the Congo red-stained paraffin sections revealed a typical pale pinkish to pink-reddish colour in bright light (Fig. 4d, Supplementary Fig. 34).

Moreover, to verify this new targeting strategy promoted tumour competition for the nanomaterial against other organs, especially reticuloendothelial system (RES)-rich organs, we harvested the major organs of mice for ex vivo imaging at 48 h (Fig. 4e). Biodistribution studies of molecules 1 (positive for both recognition and self-assembly), 2 (positive only for self-assembly) and 3 (positive only for recognition) at 48 h post administration showed that molecule 1 displayed very different distribution patterns in major organs and tumours from those of molecules 2 and 3 (Fig. 4e). The concentration of molecule 1 in tumour tissue was significantly higher than that of molecules 2 and 3 (Fig. 4e). This result implied that with the new targeting mechanism, molecule 1 self-assembled in situ after molecular cleavage and exhibited higher retention efficiency in the tumour site, which increased the tumour competition for the nanomaterial against other organs. Next, to systematically understand the in vivo behaviour of molecule 1, we quantitatively compared the molecular distribution in major organs and the tumour through pharmacokinetics experiments. Herein, the pharmacokinetic parameters were analysed using the DAS2.0 software and the molecular concentrations in the blood were determined by fluorescence emission intensity. As a result, the blood circulation half-life (*t*_1/2β_) values of molecules 1, 2 and 3 were 1.15 ± 0.10, 0.91 ± 0.23 and 1.06 ± 0.17 h, respectively (Fig. 4f). Furthermore, the molecular concentrations in tissues were identified by the fluorescence emission intensity found in tissue lysates by Maestro II. We observed a linear correlation between the molecular concentration and the corresponding fluorescence intensity (Supplementary Fig. 35). After the administration of molecules 1, 2 and 3 to mice for 48 h, the concentration of molecule 1 at the tumour site increased to 9.2 ± 0.5% ID/g, which was higher than that of 2 (4.2 ± 0.1% ID/g) and 3 (5.3 ± 0.8% ID/g) at 48 h post injection, because the new targeting mechanism induced tumour accumulation (Fig. 4g, Supplementary Fig. 36). We selected peptide-modified liposomes (Supplementary Figs. 37 and 38) and Pep-nanofibers (Supplementary Fig. 12) formed by self-assembly of molecule 5 as control groups. In Fig. 4h, the accumulation efficiency of molecule 1 (9.2 ± 0.5% ID/g) (48 h IV injection) was much higher than that of Pep-nanofiber (4.7 ± 0.3% ID/g) (Supplementary Fig. 39) and liposome (3.2 ± 1.3% ID/g) (Supplementary Fig. 40). It was also much higher than those of small molecules (<3% ID/g)^1,31^ and nanomaterials (6.2 ± 5.1% ID/g) (Supplementary Fig. 41)^32^ as reported in mouse models in the literature of the past 10 years. Furthermore, the pharmacokinetic parameters were analysed as previously described. According to the concentration–time curve, from 0 to 48 h (AUC_0–48h_), the concentration of molecule 1 was six-fold and three-fold higher than that of 2 and 3, respectively (Supplementary Table 1). It was also shown that molecule 1 was highly accumulated in tumours from *T*_max_ and *C*_max_. Meanwhile, we noted the elimination half-life and mean retention time (MRT) of molecule 1 (69.3 ± 0, 23.4 ± 0.2 h) were significantly longer than that of molecule 2 (10.0 ± 1.9, 15.2 ± 4.5 h) and 3 (10.6 ± 2.8, 10.7 ± 1.8 h). Meanwhile, we noted that molecule 1 showed a similar distribution and clearance behaviour to molecules 2 and 3 at 2 h. Moreover, clearance of medicine (CL) of molecular 1 removal was significantly slower than that of molecules 2 and 3. These results suggested that molecule 1 exhibited small-molecule behaviour in major organs other than the tumour. The imaging of tissue lysates at different times showed that molecules 1, 2 and 3 followed a clearance pathway comparable to that of small molecules. The strong accumulation of molecule 1 observed in kidneys and liver were indicative of excretion from the organs of the RES, and the imaging of urine at different time points also indicated renal excretion (Fig. 4i, Supplementary Figs. 42 and 43). Interestingly, via the imaging of faeces collected at different time points, we found that a portion of molecules 1, 2 and 3 are excreted from the gastrointestinal tract, which also illustrated hepatic excretion (Supplementary Fig. 43). In the case of liver uptake, molecule 1 exhibited minimal liver retention (4.1 ± 0.2% ID/g, 48 h injection), compared with control groups of Pep-nanofiber (22.7 ± 1.9% ID/g) and liposome (11.1 ± 4.9% ID/g) and nanomaterials (Fig. 4j, Supplementary Figs. 39–41)^32^ reported by literature reviewed for the past 10 years in mouse models. All of the above results indicate that the TCASS significantly enhanced molecular accumulation and retention in the tumour region with nanomaterial-like behaviour and achieved a comparable clearance pathway for small molecules, which were excreted from the organs of the RES, such as the liver and kidney, and from the gastrointestinal tract.

**Fig. 5:**
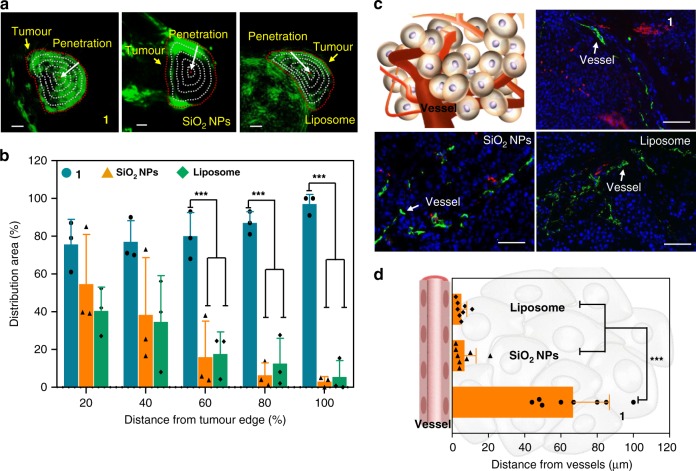
Tumour penetrations of TCASS. **a** 3D reconstruction PA images of tumour-bearing mice injected with molecule 1, SiO_2_ nanoparticles (SiO_2_ NPs) and liposome labelled by Cy. The degrees of penetration into the tumour were calculated. To better illustrate the penetration extent of molecule 1, SiO_2_ nanoparticles (SiO_2_ NPs) and liposome labelled by Cy, the tumour tissue was divided into five parts with identical radius intervals, which indicated the trend from the tumour edge to the interior (the red dashed circles show the tumour margin, and the grey dashed circles show the normalized distance from the tumour edge). **b** Quantification of the PA signal intensity. The PA intensity in the intratumoural distribution area between neighbouring polygons was normalized by the corresponding area. Data are presented as the mean ± s.d. (*n* = 3). ***p < 0.001, *p* values were performed with one-way ANOVA followed by post hoc Tukey’s test for the indicated comparison. **c** Frozen se**c**tions of tumour removed after treatment with molecule 1, SiO_2_ NPs and liposome labelled by Cy for 12 h. The tumour cell nucleus and vessels were stained with DAPI and FITC-tagged CD31 antibody, respectively. **d** Quantification of the penetrative distance of molecule 1, SiO_2_ NPs and liposome labelled by Cy from the tumour vessels. Data are presented as the mean ± s.d. (*n* = 8). ***p < 0.001, *p* values were performed with one-way ANOVA followed by post hoc Tukey’s test for the indicated comparison. Scale bar: **a** 2 mm; **c** 50 μm

We further investigated the tumour penetrability resulting from the TCASS, using a multispectral optoacoustic tomographic (MSOT) imaging system. Typical nanomaterials, such as SiO_2_ nanoparticles and liposomes labelled with cyanine dye with diameters of approximately 100 nm, were used as controls (Supplementary Fig. 44). We used H460 tumour-bearing mice for photoacoustic (PA) imaging (Fig. 5a). Using continuous tomography, 3D tumour images were reconstructed with a space interval of 0.2 mm and a range of 2 cm for precise spatial location. The measured results indicated that molecule 1 was distributed unevenly throughout the full extent of the tumour tissues (Fig. 5a). In addition, the SiO_2_ nanoparticles and liposome remained distributed only in the margins of the tumour tissues (Fig. 5a). The quantification of the signal distribution area in the tumours showed that the distribution area of molecule 1 was larger than that of liposomes and SiO_2_ nanoparticles at 12 h post injection when the latter invaded into more than 70% of the depth of the tumour (Fig. 5b). This enhanced tumour accumulation and penetration were also validated by the histological examination of cryosections of the H460 tumours. To evaluate the penetration efficiency, we labelled the tumour vessels with fluorescent-tagged antibodies against the endothelial marker CD31. As depicted in Fig. 5c, although the liposomes and SiO_2_ nanoparticles could be observed, most of them were still localized around the blood vessels. However, molecule 1 penetrated much further into the tumour, away from the vessels, and occurred in a larger area of the tumour tissue than the liposomes and SiO_2_ nanoparticles. We further used simulated scatter diagrams to confirm the excellent penetration of molecule 1. The signal of molecule 1 was found to show approximately 67.4 ± 10.3 μm from the blood vessels, and the signals of the liposomes and SiO_2_ nanoparticles were at approximately 4.9 ± 3.1 and 6.8 ± 5.8 μm from the blood vessels (Fig. 5d). It is important to consider that improvements in nanomaterial penetration can result in significant enhancements to diagnostic and therapeutic effectiveness. Moreover, molecule 1 had an enhanced penetration distance from the blood vessels of approximately 13 and 10 times that of liposomes and SiO_2_ nanoparticles. We also altered the surface modification, size and charges of liposomes, but limited tumour penetration was achieved by this commonly used strategy^52^. These results confirmed that the TCASS showed a higher tumour penetrating efficiency, which was consistent with the in vivo imaging data.

### In vivo antitumor efficacy of TCASS

**Fig. 6:**
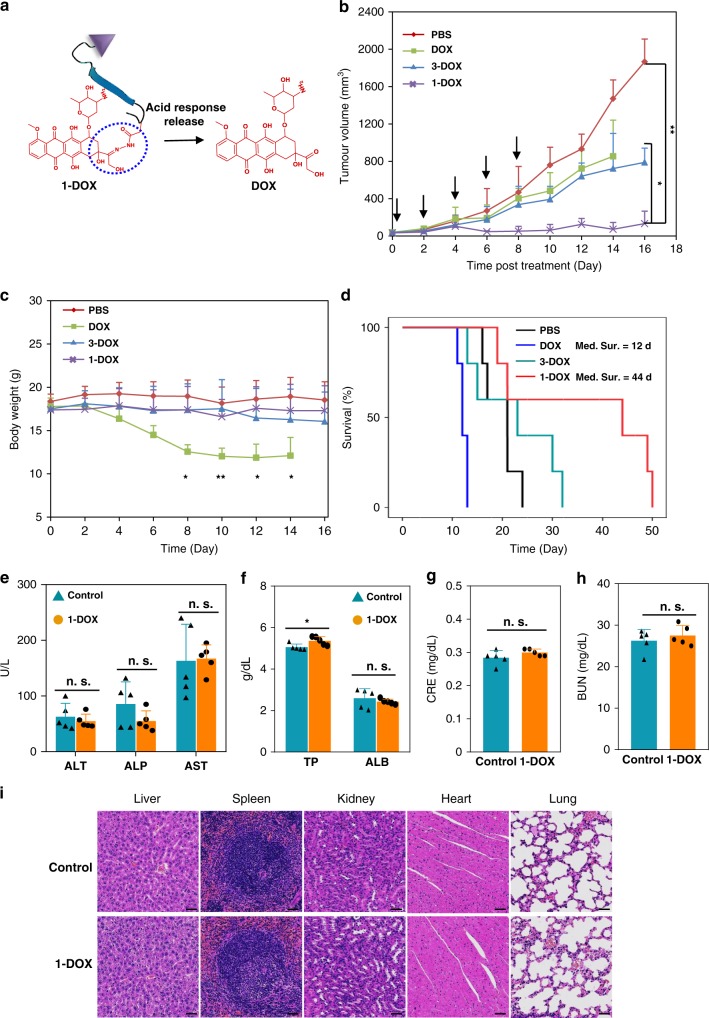
In vivo antitumor activity evaluation of TCASS. **a** Schematic diagram of molecule 1-DOX (positive for both recognition and self-assembly). **b** Tumour growth inhibition. **c** Body weight changes of H460 tumour-bearing nude mice after treatment with PBS, DOX (2.0 mg/kg), 3-DOX (an identical DOX dose of 2.0 mg/kg), 1-DOX (an identical DOX dose of 2.0 mg/kg). The black arrow indicates the administrated time point. **d** Kaplan–Meimer survival curve of H460 tumour-bearing nude mice. **e**–**i** Blood biochemistry and haematology data of the H460 tumour-bearing nude mice which were treated with molecule 1-DOX (an identical DOX dose of 2.0 mg/kg, once every other day for a total of five times). The control group was H460 tumour-bearing nude mice without treatment. **e** Major indicators of liver function, alanine aminotransferase (ALT), alkaline phosphatase (ALP) and aspartate aminotransferase (AST) levels. **f** Total protein (TP) and albumin (ALB) levels. The creatinine (CRE) (**g**) and blood urea nitrogen (BUN) (**h**) levels, important indicators of kidney function. **i** Histology evaluation of the major organs (liver, spleen, kidney, heart and lung) collected from the control group without treatment and molecule 1-DOX treatment group. Data are presented as mean ± s.d. (*n* = 5). *P* values were performed with the Cox Mantel log-rank test. n.s. means not significant, *p* values *p < 0.05, **p < 0.01, one-way ANOVA followed by post hoc Tukey’s test for the indicated comparison. Scale bar: 20 μm

To verify the promising application of TCASS in biomedicine, we synthesized a new molecule with a clinically used chemotherapy drug (doxorubicin, DOX)^53–55^ as a payload (1-DOX, positive for both recognition and self-assembly) and 3-DOX as a control molecule (positive only for recognition) (Supplementary Figs. 45 and 46). The molecule 1-DOX exhibited better cellular internalization capability compared with free DOX in H460 cells (Supplementary Fig. 47). Once the molecule 1-DOX got into cells, the acid-sensitive hydrazone bond would be cleaved^53,56^ (Fig. 6a) and then free DOX could enter the nucleus (Supplementary Fig. 47). Next, by cytotoxicity assay, we show that the molecule 1-DOX (100 nM) had a significant inhibitory effect on tumour cells (H460) compared with free DOX (100 nM) and the molecule 3-DOX (100 nM) (Supplementary Fig. 48). Finally, we investigated the therapeutic effect of the molecule 1-DOX in vivo using the H460 tumour-bearing nude mouse model. The antitumor efficacy of various formulations is illustrated in Fig. 6. The tumours treated with PBS grew exponentially over time and exhibited an average tumour volume of 1865 mm^3^ after 16 days (Fig. 6b). In the DOX treatment group, a moderate tumour inhibition was achieved, with the mean tumour volume of 854 mm^3^ after 14 days. Importantly, the molecule 1-DOX exhibited a stronger antitumor efficiency than both 3-DOX and free DOX with the mean tumour volume of 133 mm^3^ after 16 days. As an indicator of systemic toxicity, body weight was observed and measured closely (Fig. 6c). As we expected, there was no apparent loss of body weight in mice treated with 1-DOX during the days of injection compared with free DOX, implying the reduced systemic toxicity of 1-DOX. Meanwhile, the survival time of mice treated with 1-DOX (median survival time (Med sur.) = 44 days) was significantly prolonged over the group treated with DOX (median survival time (Med sur.) = 12 days) (Fig. 6d). The histological analysis and haematology analysis of 1-DOX at the end of the therapeutic experiments in vivo were carried out. The results indicated that there was no significant difference between 1-DOX and control group in blood biochemistry analysis including ALT, ALP, AST, ALB, CRE and BUN (Fig. 6e–h). Although TP had a slight increase, it was still in the normal range. All the results of blood biochemistry showed no significant liver or kidney toxicity. Moreover, no evident damage was observed in histological analysis (Fig. 6i). These results demonstrated that 1-DOX was safe and tolerated in vivo experiments. The haematology data were basically within the normal range except for a few slight fluctuations in several parameters, including white blood cells, red blood cells, haemoglobin, mean corpuscular volume, mean corpuscular haemoglobin, mean corpuscular haemoglobin concentration, platelets and haematocrit after treatment with molecule 1-DOX compared with normal range (Supplementary Fig. 49). Therefore, the substantially prolonged survival and severely impaired tumour progression manifested that the payload (1-DOX) based on the TCASS presented an effective antitumor effect with negligible systemic toxicity. It was convinced that our system potentially enables the delivery of various biotherapeutics, e.g., contrast agents, chemotherapy drug, peptides and even small molecular proteins, resulting in highly efficient accumulation and retention at tumour site, and exert their biological effects after that.

### Ex vivo imaging of human intact bladder of TCASS

**Fig. 7:**
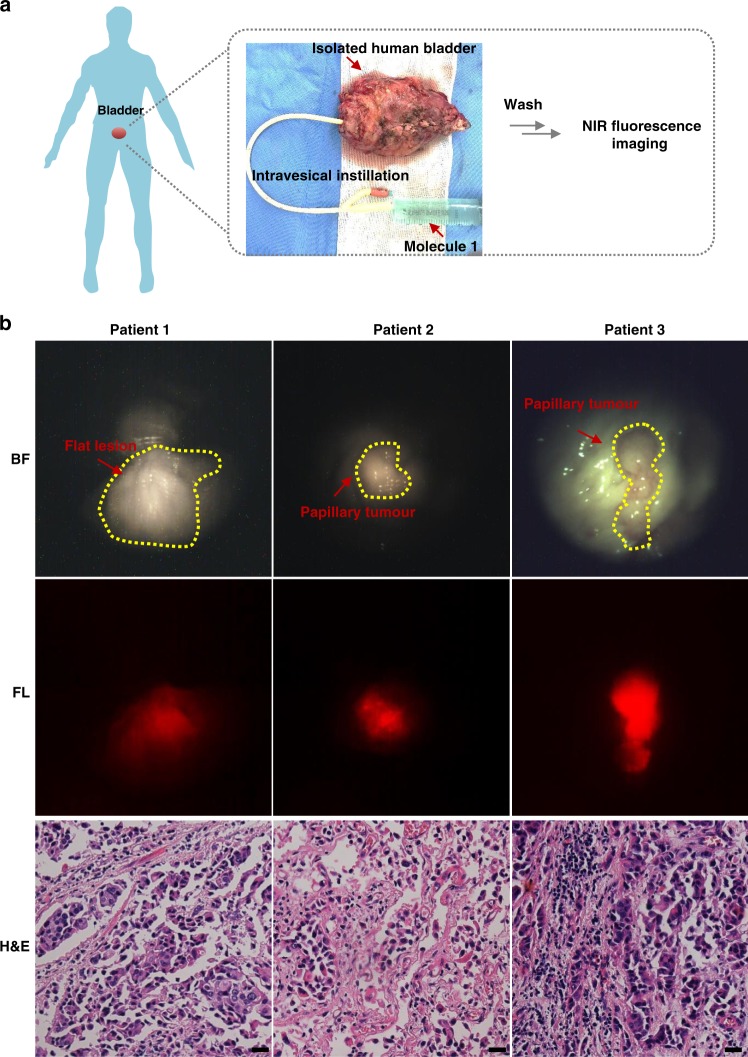
Ex vivo imaging of human intact bladder of TCASS. **a** Schematic illustrated the procedure of intravesical instillating the molecule 1 using ex vivo human intact bladder. **b** NIR fluorescence imaging of human bladder tumour tissues of three patients and their corresponding H&E stained. Scale bar: 20 μm

Bladder, as an easily accessible hollow organ, provided a well enclosed space for intravesical instillation of agents in clinic^57^. This intravesical administration method minimized potential systemic toxicity of compounds including chemo-drugs, imaging agents, etc.^57^. Like 5-ALA was widely utilized for clinical intravesical instillation^58^, we used the same method to treat the EJ orthotopic bladder cancer mice model and the isolated intact bladder with molecule 1. Firstly, mice were sacrificed after instilled and incubated 100 uL molecule 1 in PBS for 1 h. Then, the bladder was removed and placed in a blackboard for ex vivo fluorescence imaging. As a result, the fluorescence signal was observed on the tumour site (Supplementary Fig. 50). Moreover, the corresponding histopathology of these tissues with the fluorescence signal was confirmed as tumour tissues (Supplementary Fig. 50), further demonstrated the high sensitivity of the TCASS. In addition, XIAP was overexpressed in bladder cancer tissues compared with normal urothelium tissues^59^. Owing to these characteristics of bladder cancer, we speculated that our strategy could specifically target bladder cancer tissues through intravesical administration. Moreover, we obtained fresh isolated intact bladder from patients who underwent radical cystectomy, then intravesical instillation of molecule 1 according to the method described (Fig. 7a). The imaging results exhibited red fluorescence around the tumour site (Fig. 7b, Supplementary Movie 1), especially on flat lesion (Patient 1). Additionally, the corresponding histopathology of these lesions with the binding of molecule 1 was confirmed as tumour tissues (Fig. 7b), further demonstrated the high sensitivity of the TCASS. By contrast, no obvious fluorescence was detected on the normal urothelium. This evident distinguishment made clear tumour boundaries detection which had a great clinical value in imaging-guided surgery of bladder cancer. The signal intensity between the tumour tissue and the surrounding normal tissue was analysed statistically. The signal-to-noise ratio of the tumour area was up to 2.7 times (Supplementary Fig. 51). Above all, the ex vivo imaging results of human intact bladder indicated the high specificity and sensitivity of the TCASS in detecting bladder cancer, also showed a great potential clinical translation value in the diagnosis and surgical treatment of bladder cancer.

### Toxicology evaluation of TCASS

Potential in vivo toxicity of nanomaterials has been a major concern in the nanomedicine community. The mice were sacrificed after treatment with molecule 1 (14 mg/kg) on the 3, 7 and 15 days for blood collection. During our experiments, we did not notice any obvious signs of acute toxicity for molecule 1-injected mice within 15 days. To reveal any potential toxic effect of molecule 1 on the treated mice, we performed blood biochemistry and haematology analyses. As a result, the levels of major liver function markers, including alanine aminotransferase (ALT), aspartate aminotransferase (AST), alkaline phosphatase (ALP) and the albumin concentration, were all within reference normal ranges^60,61^, indicating that no obvious hepatic toxicity was induced by molecule 1 (Supplementary Fig. 52a). The assay of the activities of total protein (TP), albumin (ALB), globulin (GLO) and the A/G (ALB/GLO) ratio as indices for liver damage showed no apparent toxicity (Supplementary Fig. 52b). Next, we examined the creatinine (CRE) and blood urea nitrogen (BUN) levels in the blood of treated mice which were important indicator of kidney function, were also within the normal range (Supplementary Fig. 52c). Furthermore, for the haematological assessment, we selectively analysed the major indicator like white blood cells, red blood cells, haemoglobin, mean corpuscular volume, mean haemoglobin, mean corpuscular haemoglobin concentration, platelet count and haematocrit (Supplementary Fig. 52d). All above indices in the treated groups did not show obvious changes compared with the control groups which were within the reference normal ranges^60,61^. Therefore, no appreciable toxicity of molecule 1 was observed in the blood biochemistry and haematological data. Finally, in the histology evaluation, slightly enlarged splenic corpuscle and expanded germinal centres were observed. These changes probably came from the slightly inflammation and also might be related to environmental factors. Meanwhile, the rest organs had no obvious abnormalities (Supplementary Fig. 52e). Above results concluded that the molecule 1 displays no obvious toxicity at the treatment dose, which was highly encouraging for applications in nanomedicine.

## Discussion

In summary, we presented a TCASS consisting of (i) a tumour-specific recognition motif, (ii) an enzymatically cleavable linker, (iii) a self-assembly motif and (iv) a functional molecule. We systematically studied the TCASS in different mouse models to optimize the tumour nanomedicine, including accumulation, penetration and organ competition. TCASS exhibited high-efficiency molecular accumulation and retention in tumour tissue. Moreover, this system enhanced tumour penetration compared with that of typical nanomaterials (such as SiO_2_ nanoparticles and liposomes with diameters of approximately 100 nm). Meanwhile, the organ competition behaviour of the TCASS was comparable to that of small molecules, which were rapidly excreted from the liver and kidneys, which linked directly to the reduced nanotoxicity. Furthermore, by integration of chemodrug (e.g., DOX), the TCASS has an enhanced therapeutic effect and negligible systemic toxicity. Importantly, the significantly prolonged median survival time further convinced the therapeutic outcome of this strategy. Finally, by integration of a contrast agent, our TCASS has a high specificity and sensitivity for detecting bladder cancer in an isolated human intact bladder, verifying its potential clinical translation. This study provided a de novo concept for tumour targeting that might be more efficient than active/passive targeting mechanisms.

## Methods

### General

All relevant characterizations of molecules, cellular imaging experiments, molecular biology experiments and toxicology evaluation experiments are described in the Supplementary Methods.

### Synthesis of materials

The peptides were synthesized by conventional solid phase peptide synthesis (SPPS). The 9-fluorenylmethoxycarbonyl (Fmoc) coupling chemistry on the Wang-resin was used. The Fmoc-protection groups were removed by piperidine/DMF (v/v = 1:5) solution. The Fmoc protected amino acids (10 equiv.) were dissolved in DMF. (2-(1*H*-benzotriazol-1-yl)-1,1,3,3-tetramethyluronium hexafluorophosphate (HBTU, 5 equiv.) was dissolved in 0.4 M 4-methylmorpholine (NMM)/DMF solution as a coupling reagent. The peptide was deprotected and cleaved from the resin by reacting with the mixture of trifluoroacetic acid (TFA, 92.5%, v/v), H_2_O (2.5%, v/v), 1,2-ethanedithiol (EDT, 2.5%, v/v) and triisopropylsilane (TIPS, 2.5%, v/v) for 2.5 h in ice bath. The peptide was precipitated into cold diethyl ether and dried under vacuum. The peptides were characterized with mass spectrometry (MALDI-TOF) and HPLC. The peptide was reacted with cyanine dye (Cy) (1.1 equiv) for 2 h in PBS. And then the final reaction solution was dialysed against deionized water to obtain a green solid with a 95–99% yield. The compounds were characterized with HPLC, mass spectrometry (MALDI-TOF) and nuclear magnetic resonance (H^1^ NMR).

### Bio-TEM of the ultrathin sections of cells

H460 cells (3111C0001CCC000355) and 293T cells (3111C0002000000112) were treated with molecule I-1 for 12 h, then fixed in 3% glutaraldehyde overnight at 4 °C, further washed triple with PBS, and fixed in buffer (Dalton’s osmium in potassium dichromate) for 1.5 h. Next, treated-H460 cells were rinsed three times in PBS. Subsequently, samples were dehydrated through increasing concentration of acetone solutions (10 min each, 50%, 70%, 80%, 96%, three changes of 100%), then utilize 1:1 and 1:2 propylene oxide Epon 812 mixture for twice, respectively. Finally, the sample infiltrated with pure Epon 812 at 4 °C overnight, then solidified at 37 °C, 45 °C and 60 °C for 24 h, respectively. When the samples were accomplished, they were sectioned with a thickness of 70 nm, either transverse section or longitudinal section. Next, the sections were put onto copper grids; post-stained in 2% uranyl acetate in 70% ethanol for 0.5 h rinsed with H_2_O; then incubated the grids in 3% lead citrate for another 10 min rinsed with H_2_O. The sections were observed by a HT7700 Transmission Electron Microscope under 80 kV. The functional peptide I-1 was labelled with iodine and further identified the nanofibrils through energy dispersive spectra.

### Western blotting

The lysis buffer which contains 50 mM Tris-HCl (pH 8.0), 150 mM NaCl, 1% (v/v) Triton-X 100 and protease inhibitor was used to re-suspend the pretreated cells. The estimation of the protein content was accomplished by a BCA kit (Applygen). Each sample (60 μg of protein) was subjected to SDS-PAGE and then transferred onto nitrocellulose membrane. Blots were blocked in a blocking buffer containing 5% (wt/v) non-fat milk, 0.1% (v/v) Tween-20 in 10 mM TBS, incubated overnight with primary antibodies at 4 °C on a shaker, incubated with an appropriate secondary antibody for 1 h at room temperature on a shaker and subsequently scanned on a Typhoon Trio Variable Mode Imager. Pretreated cells were re-suspended in lysis buffer containing 50 mM Tris-HCl (pH 8.0), 150 mM NaCl, 1% (v/v) Triton-X 100 and protease inhibitor. The protein content was estimated using a BCA kit (Applygen). Each sample (60 μg of protein) was subjected to SDS-PAGE and then transferred onto nitrocellulose membrane. Blots were blocked in a blocking buffer containing 5% (wt/v) non-fat milk, 0.1% (v/v) Tween-20 in 10 mM TBS, incubated with primary antibodies overnight at 4 °C, incubated with an appropriate secondary antibody for 1 h at room temperature and subsequently scanned on a Typhoon Trio Variable Mode Imager. The uncropped and unprocessed scans are shown in Supplementary Figs. 18 and 33.

### Fluorescence imaging and pharmacokinetics in vivo

Animal experiments were performed in accordance with the Guide for the Care and Use of Laboratory Animals and approved by the Institutional Animal Care and Use Committee (IACUC) of the National Center for Nanoscience and Technology (NCNST), China. Female Balb/c nude mice (6–8 weeks, 18–22 g) were purchased from Vital River Laboratory Animal Technology Co. Ltd. H460 cells were subcutaneously implanted into these mice on the right flank. Then the mice were randomly grouped (*n* = 3). When the tumour grew to a single aspect of about 0.5 cm, we injected the molecules 1, 2 and 3 intravenously in the same dose of 14 mg/kg in PBS solution, respectively. Fluorescence images were measured by a Maestro II in vivo imaging system (CRi) at 1, 2, 4, 6, 8, 12, 24, 48, 72, 96 and 120 h post injection. Further, tumours and other major organs (i.e., heart, liver, spleen, lung and kidney) were collected for ex vivo fluorescence imaging at 48 h post injection. The organ biodistribution of the 1, 2 and 3 was examined using H460 xenografts mice. The mice were sacrificed at 2, 4, 8, 12, 24 or 48 h post injection. Finally, all the major organs and tumour were collected, weighed, and homogenized (0.3 mL of lysis buffer per 100 mg of tissues). The homogeneous tissue solutions were obtained for fluorescence imaging by IVIS Spectrum CT. Known concentrations of compound 1, 2 or 3 are used to make the standard curve (concentration from 2.8 to 25.2 μg/mL), the “Predicted R-Squared” value of 0.99. Then we utilized this standard curve to measure the molecule content in each tissue. In addition, EJ xenograft tumour mice model was built, as above described, to validate the generality of the strategy.

### In vivo photoacoustic imaging

All animal experiments were performed as previously described. H460 cells were also subcutaneously implanted on the right flank of xenografts mice. After the tumour had developed, Molecule 1 (14 mg/kg, *n* = 3), liposome and SiO_2_ nanoparticles labelled by Cy (the same molar amount of Cy, *n* = 3) dissolved into PBS was intravenously injected through tail vein of mice. The control group was PBS at the same conditions. After injection, the mice were scanned with MOST (mode: MOST 128) at 12 h post injection individually, then the averaged PA signals of the tumour areas were obtained with the ViewMSOT software. Next, 3D reconstructions were generated from the PA images with Image J software. The continuous tomography was used with a space interval of 0.2 mm in a range of 2 cm. In order to quantify the PA signal intensity, the intratumoral distribution area was normalized with the tumour regions.

### In vivo antitumor efficacy

All H460 xenografts mice were built as described above. When the tumour volume reached 50 mm^3^, the mice were randomly grouped (*n* = 5 in each group) and i.v. administrated with PBS, DOX, 3-DOX and 1-DOX, under an identical DOX dose of 2.0 mg/kg. Treatment is administered once every other day for a total of five times. The body weight and the tumour volume were measured once every other day. Tumour volume (*V*) was calculated by the following equation: *V* = *LW*^2^ /2, where *L* was the longer and *W* was the shorter diameter (mm). Mice with a tumour volume of more than 2000 mm^3^ or a 20% weight loss are a humanitarian endpoint. Kaplan–Meimer survival curve was compared using the Cox Mantel log-rank test. Statistical analyses were performed using SPSS (Statistical Package for the Social Sciences) version 22.0.

### Toxicology evaluation

The healthy female Balb/c nude mice (6–8 weeks, *n* = 3) treated with molecule 1 was systematically investigated over 15 days. The mice were sacrificed after treatment with molecule 1 at a dose of 14 mg/kg on the 3rd, 7th and 15th day for blood collection. The blood biochemistry and haematology analyses were performed by Vital River Laboratory Animal Technology Co. Ltd. The histology evaluation was performed by Servicebio. All the toxicology evaluation was carried out in blind manner.

### Establishment of the bladder orthotopic tumour mouse model

Mice were intraperitoneally anaesthetised with Pentobarbital sodium (40 mg/kg body weight) and fixed in the abdominal position. Then, the abdomen was gently squeezed to empty the bladder. Under sterile conditions, mice were catheterized with a modified IV catheter (24G) to slightly damage the inner mucosa of the bladder. Subsequently, 100 μL of EJ cells (1 × 10^7^) was infused and incubated in the bladder for 2 h. Finally, the mouse urethra was clamped to prevent tumour cells from overflowing along the urethra. The growth of the mice was observed daily to observe the presence or absence of gross haematuria as well as the growth of the bladder mass by palpation of the abdomen. After 3 weeks, a 1 cm incision is made through the skin and abdominopelvic wall, anddr then the bladder was exteriorized to confirm tumour growth.

### Intact bladder specimen selection and preparation

After obtaining approval from the institutional review board of the Fourth Hospital of Harbin Medical University, patients with urothelial carcinoma were scheduled for radical cystectomy and appropriate informed consent was obtained. Fresh intact bladder was obtained from the operating room immediately after radical cystectomy and irrigated three times for 5 min via an 18-French (Fr) urinary catheter with sterile saline, and 70 mL of molecule 1 in PBS (50 μM, pH 7.4) was instilled and incubated at 37 °C for 1 h. Then, the unbound molecule 1 was removed by irrigating with sterile saline three times for 5 min, the bladder was opened longitudinally on the anterior wall. Ex vivo fluorescent and white light imaging of the bladder cancer was performed using a near infrared imaging system. To explore the correlation between appearance of fluorescent signal and cancer lesions, standard pathological analysis of represented fluorescent spots were carried out after imaging.

### Statistical methods

Data are mean ± standard deviation for in vitro and in vivo. Statistical significance was one-way ANOVA followed by post hoc Tukey’s test. Kaplan–Meimer survival curve was compared using the Cox Mantel log-rank test. Statistical analyses were performed using SPSS (Statistical Package for the Social Sciences) version 22.0.

### Reporting summary

Further information on research design is available in the Nature Research Reporting Summary linked to this article.

## Supplementary information

Supplementary Information

Description of Additional Supplementary Files

Supplementary Movie 1

Reporting Summary

## Data Availability

The relevant data supporting this study can be obtained in the Article and the Supplementary Information or available from the corresponding authors on reasonable request. Results are available at: https://figshare.com/s/ebcaae7131e15db43ee7.
