# Supplementary material for: A tumour-selective cascade activatable self-detained system for drug delivery and cancer imaging
Source: Nat Commun. 2019 Oct 24;10:4861. doi: 10.1038/s41467-019-12848-5 (PMC6813295; doi:10.1038/s41467-019-12848-5)
Supplement: Supplementary file 1 — Supplementary Information [file 41467_2019_12848_MOESM1_ESM.pdf]

Supplementary Information

A tumour-selective cascade activatable self-detained system for drug delivery and cancer imaging

Hong-Wei An,[1,2,3] Li-Li Li,[1] Yi Wang,[1,2] Ziqi Wang,[4] Dayong Hou,[4] Yao-Xin Lin,[1,2] Sheng-Lin Qiao,[1,2] Man-Di Wang,[1,2] Chao Yang,[1] Yong Cong[1,2], Yang Ma,[1,2] Xiao-Xiao Zhao,[1,2] Qian Cai,[1] Wen-Ting Chen,[1] Chu-Qi Lu,[1] Wanhai Xu,[4,*] Hao Wang[1,2,*] and Yuliang Zhao[1,2,3,*]

1 CAS Center for Excellence in Nanoscience, CAS Key Laboratory for Biomedical Effects of Nanomaterials and Nanosafety, National Center for Nanoscience and Technology (NCNST). No. 11 Beiyitiao, Zhongguancun, Beijing, 100190, China.

2 Center of Materials Science and Optoelectronics Engineering, University of Chinese Academy of Sciences, No. 19A Yuquan Road, Beijing 100049, China.

3 Institute of High Energy Physics, Chinese Academy of Sciences (CAS), Yuquan Road, Beijing, 100049, China.

4 Department of Urology, the Fourth Hospital of Harbin Medical University, Heilongjiang Key Laboratory of Scientific Research in Urology, Harbin, 150001, China.

* Correspondence: xuwanhai@hrbmu.edu.cn or wanghao@nanoctr.cn or zhaoyl@nanoctr.cn.

Supplementary Methods

All the reagents and solvents for organic synthesis were purchased from commercially available sources and used without further purification unless otherwise stated.

Chemicals and reagents	Commercial Companies
L-a-Phosphatidylcholine(PC);<br>Cholesterol(CH)	Avanti Polar Lipids
1,2-distearoyl-sn-glycero-3-phosphoethanolamine-N-[NHS (polyethyleneglycol) 2000] (DSPE-PEG-NHS)	Nanocs
Piperidine;<br>Human recombinant caspase-3;<br>bisBenzimide H 33342 trihydrochloride	Sigma-Aldrich Chemical Co
O-(benzotriazol-1-yl)-*N*, *N*, *N'*, *N'*-tetramethyluronium hexafluorophosphate (HBTU);<br>4-methylmorpholine (NMM);<br>Triisopropylsilane (TIPS);<br>Hexafluoroisopropanol (HFIP);<br>Trifluoroacetic acid (TFA);<br>1,2-Ethanedithiol	TCI
Fmoc-amino acids;<br>Wang resins	GL Biochem. (Shanghai) Ltd
zVDK-FMK (caspase inhibitor)	Selleck Chemicals LLC

The other solvents	Sinopharm Chemical Reagent Beijing Co., Ltd.
Anti-XIAP antibody (ab21278, 1:1000)	abcam
Chlorpromazine	Solarbio
Oligomycin A	Meilunbio

Fluorescence spectra and UV-vis spectra were recorded using an F-280 fluorescence spectrophotometer and a Shimadzu 2600 UV-vis spectrophotometer respectively. Circular dichroism (CD) spectra and wide-angle X-ray scattering (WAXS) studies were carried out using a *J*-1500 Circular Dichroism Spectroscope and a WZX-SAXS/WAXS diffractometer. The morphology of the aggregates was observed by Tecnai G2 20 S-TWIN transmission electron microscopy. Mass spectra were obtained by Microflex LRF MALDI-TOF. Photoacoustic images and signal data were measured and analyzed using a MSOT 128 Multi-Spectral Optoacoustic Tomography device.

Cellular imaging experiment. H460 (3111C0001CCC000355), and 293T (3111C0002000000112) cell lines were purchased from National Infrastructure of Cell Line Resource. They cultured in 1640 (H460) and DMEM (293T cells) with 10% (v: v) fetal bovine serum, 1% penicillin–streptomycin in an incubator (Thermo Scientific) at 37 °C under an atmosphere of 5% CO_2 and 95% relative humidity. H460 and 293T cells (1 x 10^5 cells per well) were seeded in confocal microscopy dishes (MatTek), respectively. After cultured for 24 h, the cells were incubated with molecule **1** (50 μM) at 37 °C for 1 h and followed by PBS washing and fresh medium replacement for further incubation 1 h, 6 h, 12 h, 24 h and 48 h, respectively, and then washed twice by PBS buffer at 4 °C. Subsequently, the cells were stained with Hoechst 33342 (1 mg ml^{-1}; Life Technologies) at 37 °C for 10 min. Finally, the cells were washed by PBS twice at 4 °C and immediately observed under confocal laser scanning

microscopy (UltraVIEW VoX).

H460 cells (1 x 10^5 cells per well) were seeded in a confocal microscopy dish (MatTek). After culture for 24 h, the cells were treated with P7 (XIAP inhibitor) (50 µM) or zVDK-FMK (caspase inhibitor) (10 µM) for 3 h and followed by PBS washing. And then the molecule **1** (50 µM) and **2** (50 µM) were individually added for 1 h and followed by PBS washing and fresh medium replacement. After further 6 h of incubation, the cells were washed twice by PBS and stained with bisBenzimide H 33342 trihydrochloride (Hoechst 33342) (1 mg ml^{-1}; Life Technologies) at 37 °C for 10 min. Finally, the cells were washed by PBS twice at 4 °C and immediately observed under confocal laser scanning microscopy (UltraVIEW VoX).

Cyanine dye (Cy) synthesis. Cyanine dye synthesis method reference to previously published work[1].

TEM sample preparation. Samples of the supramolecular aggregates were prepared by dropping a solution of molecules **P1** and **5** in 1% HFIP into aqueous solutions. The mixtures were vortexed and set aside for 24 h before analysis. Transmission electron microscope (TEM) studies were carried out on a Tecnai G2 20 electron microscope operating at an accelerating voltage of 200 keV. The TEM samples were prepared by placing nanofibrils droplets on copper grids for 5 min and removing the excess droplets. Finally, the samples were stained by uranyl acetate for 2 min, then washed with water.

The photostability of Cyanine dye. Based on the mechanism of photoreaction, the equation of photoreaction is shown as below[2]:

$-d[Cy]/dt = k_1[Cy][O_2]$ (1)

As a constant, the concentration of O_2 in the solution full of oxygen is about 3×10^{-4} mol L^{-2}. So the formulas are as follows:

$-d[Cy]/dt = k[Cy]$ (2)

$\ln([Cy]_0/[Cy]_t) = kt$ (3)

$[Cy]_0$ is the initial concentration of the molecule 1 or 4, $[Cy]_t$ is the concentration of the molecule 1 or 5 after the irradiation of *t* min. Based on Beer rule, it has the linear relation between the concentration of the molecule 1 or 4 and the absorbance in maximal wavelength for the low concentration cyanine dye. So the formula is as follows:

$\ln A_0/A_t = kt$ (4)

A_0 is the absorbance in maximal wavelength before the irradiation, and A_t is the absorbance in maximal wavelength after the irradiation. The slope of the line, *k,* is the speed constant of the photodegradation reaction.

The pathway of molecule 1 entering cells. First, specific endocytic inhibitors were used to explore uptake pathways of molecule 1. Filipin Ⅲ (5 μg/ml), chlorpromazine hydrochloride (10 μg/ml) or 5-(N, Ndimethyl) amiloride hydrochloride (10 μM) were added to H460 cells in serum-free culture medium for 1 h, at 37℃. The untreated H460 cells were used as control group. Subsequently, H460 cells were treated with molecule 1 (50 uM), after 2 h of incubation at 37 ℃ the medium was removed and the cells were washed three times with PBS and analysed by confocal laser scanning microscopy (UltraVIEW VoX).

Detection of caspase-3 activity. H460 cells and 293T cells were treated with P7 (50 μM) for 1 h. In the other group, the H460 cells were firstly treated with P6, then treated with caspase-3 inhibitor zVDK-FMK (10 μM). After treated with corresponding molecule, each group H460 cells were trypsinized and collected into 1640 cell culture media. The cells were collected by centrifugation at 600 g, 4 ℃ for 5 minutes, and the supernatant was carefully aspirated and washed once with PBS. After that, cell lysis buffer (approximately 2-10 million cells/ml) was added. After resuspended and

lysed in an ice bath at 1℃, 16000g for 15 minutes, the supernatant was transferred to a pre-cooled centrifuge tube in an ice bath. For each group, 20 µM (final concentration) Ac-DEVD-AMC and 100µl cell lysate were added to 1 ml Protease Assay Buffer [20 mM HEPES (pH 7.5), 10% glycerol, 2 mM DTT]. The reaction mixtures were incubated for 1 hour at 37 °C. The AMC released from Ac-DEVD-AMC was measured using a spectrofluorometer with an excitation wavelength of 380 nm and an emission wavelength range of 400-550 nm.

Flow cytometry analysis. A density of 1×10^5 H460 cells per well were seeded in the 6-well plates in 1640 medium which contains 10% fetal bovine serum and 1% penicillin–streptomycin, then cultured at 37 °C in a humidified atmosphere with 5% CO_2 for 16 h. Then molecules **1**, **2** and **3** (50 µM) were added to each well, and the cells were incubated for additional 6 h, 12 h and 24 h, respectively. The apoptosis of the collected cells stained by Annexin V-FITC and PI (propidium iodide) was studied by flow cytometry.

Cell viability assays. CCK-8 (cell counting kit-8) assay was carried out to investigate the cell viability. H460 cells were utilized to evaluate the cytotoxicity of molecules **1**, **2** and **3** by the CCK-8 assay. Molecules **1**, **2** and **3** were dispersed in 1640 medium with different concentrations such as 1, 5, 10, 30, 60 and 100 µM. The cells with the density of 5×10^4 cells per well were seeded in the 96-well plates in 1640 medium containing 10% fetal bovine serum and 1% penicillin–streptomycin, then cultured at 37 °C in a humidified atmosphere with 5% CO_2 for 16 h. The sample solutions (10 µL) with different concentrations were added to each well, and the cells were incubated for additional 24 h. Then each well was added with 10 µL of CCK-8 solutions and cultured for another 4 h. Microplate reader was used to measure the UV-Vis absorptions of sample wells (A_{sample}), A_{black} and control wells ($A_{control}$) at a test wavelength of 450 nm and a reference wavelength of 690 nm,

respectively. Cell viability (%) was equal to (A_{sample} - A_{blank})/ ($A_{control}$ - A_{blank}) x 100. All the experiments were performed in triplicate.

Blood circulation. To investigate the pharmacokinetics of the compound **1**, **2** and **3**, Balb/c mice were randomly divided into three groups (n = 3). Each group was intravenous (iv) injected with a single dose of 14 mg/kg of compound **1**, **2** or **3**. Blood samples were collected via the orbital venous plexus at 0.017, 0.083, 0.25, 0.5, 1, 3, 4 and 6 h post injection and collected into EDTA tubes. The supernatant of the blood samples was collected by centrifugation at 10 000 rpm for 10 min, and the content of compound **1**, **2** and **3** in the supernatant were determined using fluorescence spectrophotometric measurement. Data for known concentrations of compound **1**, **2** or **3** (concentration from 1.2 µM to 25.0 µM) were used to make the standard curve, the "Predicted R-Squared" value of 0.99.

Preparation and characteristic of liposomes. Liposomes were prepared by a thin lipid film hydration method followed by extrusion. Briefly, PC: CH: DSPE-PEG-NHS = 66: 32: 2 (molar ratio) were dissolved in a chloroform/methanol mixture (4: 1) in a round bottom flask. The lipid film was hydrated with PBS (pH 7.4) after evaporation of the chloroform/methanol solvents. After hydration, small liposomes were obtained by extrusion through 100 nm polycarbonate membranes filters 5 times. The liposomes were labeled with peptide P8 (AVPIAQKDDEEC) and 5% Cy in PBS (pH 7.4) for 3 h. The liposomes sample was stained by uranium acetate for 30 s and washed by distilled water twice. Morphologies of liposomes were measured by Tecnai G20 electron microscope.

Supplementary Figures

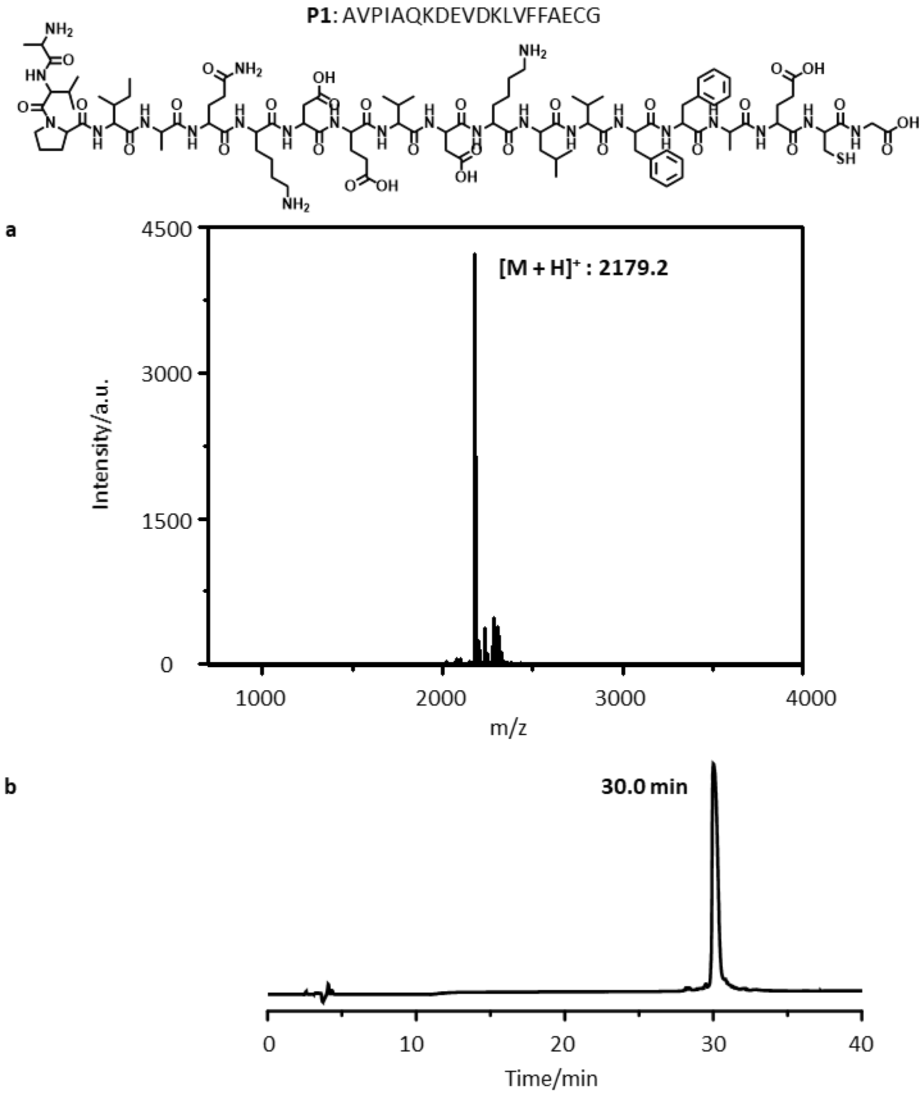

Supplementary Figure 1 | MALDI-TOF (**a**) and HPLC spectra (**b**) of **P1**. The method of HPLC spectra was as follows: solvent A, 0.1% trifluoroacetic in 100% water; solvent B, 0.1% trifluoroacetic in 100% acetonitrile; 5 min, 10% solvent B, 35 min, 50% solvent B, 40 min, 50% solvent B.

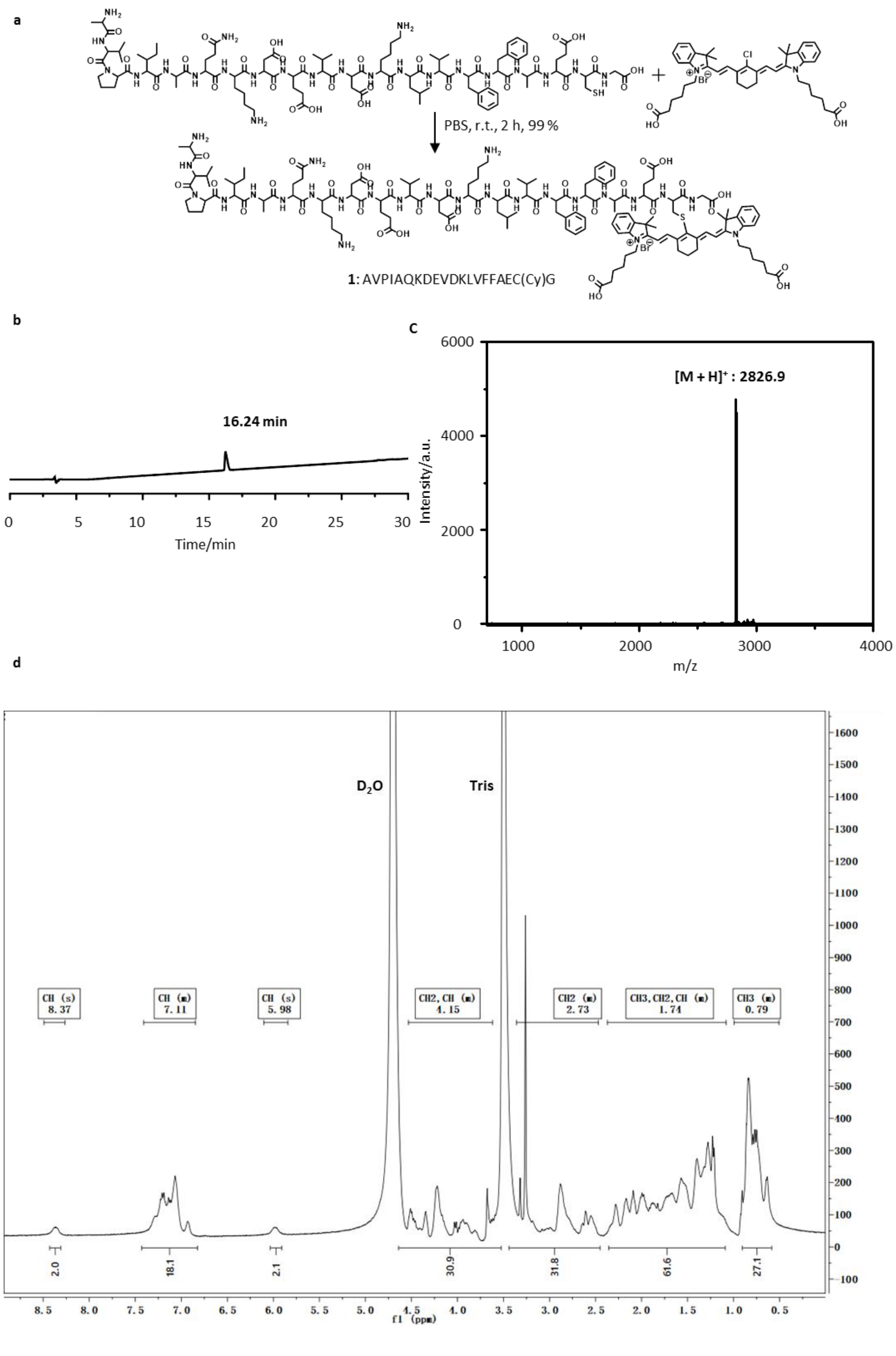

Supplementary Figure 2 | **a** The structure of molecule 1; HPLC spectra (**b**) and MALDI-TOF (**c**) molecule **1**; The method of HPLC spectra was as follows: solvent A, 0.1% trifluoroacetic in 100% water; solvent B, 0.1% trifluoroacetic in 100% acetonitrile; 0.01 min, 25% solvent B, 30 min, 90% solvent B. **d**, ^{1}H NMR of molecule **1** in D_2O. ^{1}H NMR (400 MHz, D_2O) δ 8.37 (s, 2H, CH), 7.42-6.85 (m, 18H, CH), 5.98 (s, 2H, CH), 4.53-3.62 (m, 31H, CH_2, CH), 3.36-2.47 (m, 32H, CH_2), 2.37-1.08 (m, 62H, CH_3, CH_2, CH), 1.00-0.51 (m, 27H, CH_3).

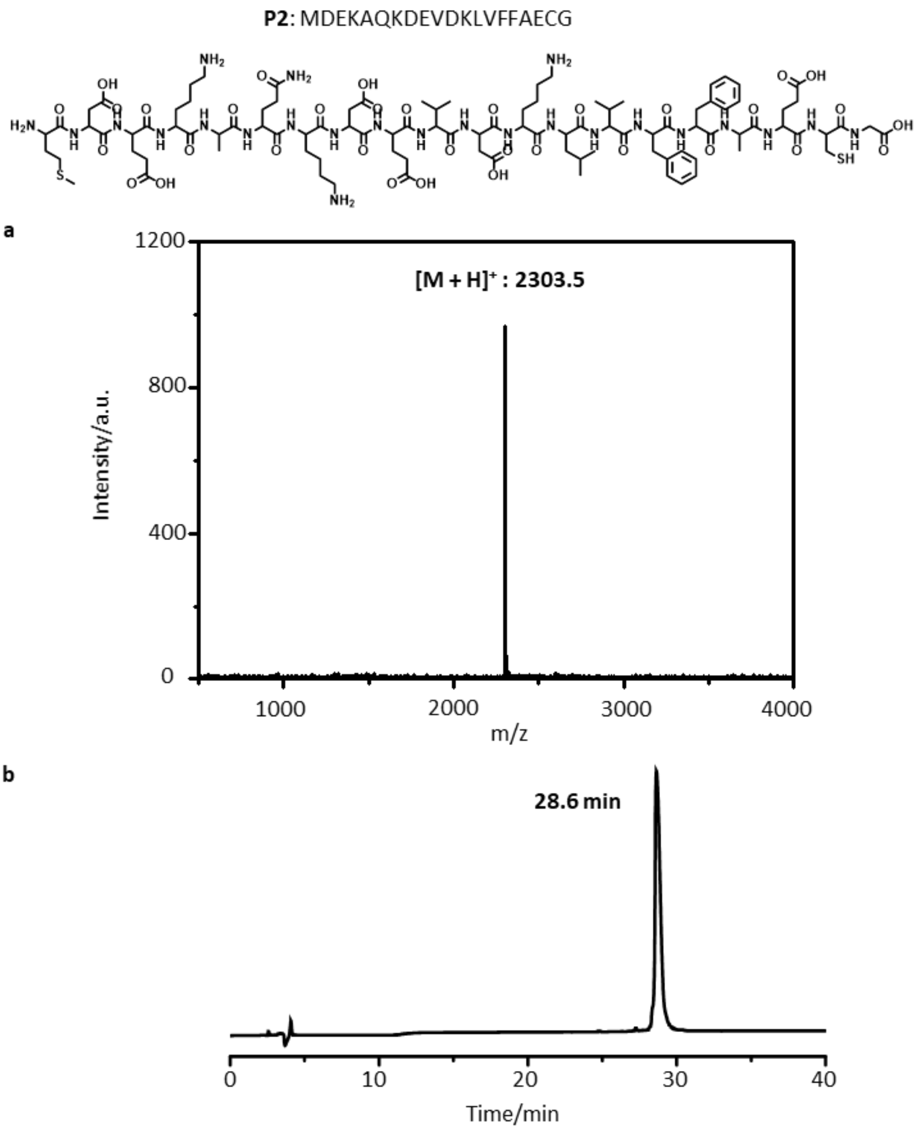

Supplementary Figure 3 | MALDI-TOF (**a**) and HPLC spectra (**b**) of **P2**. The method of HPLC spectra was as follows: solvent A, 0.1% trifluoroacetic in 100% water; solvent B, 0.1% trifluoroacetic in 100% acetonitrile; 5 min, 10% solvent B, 35 min, 50% solvent B, 40 min, 50% solvent B.

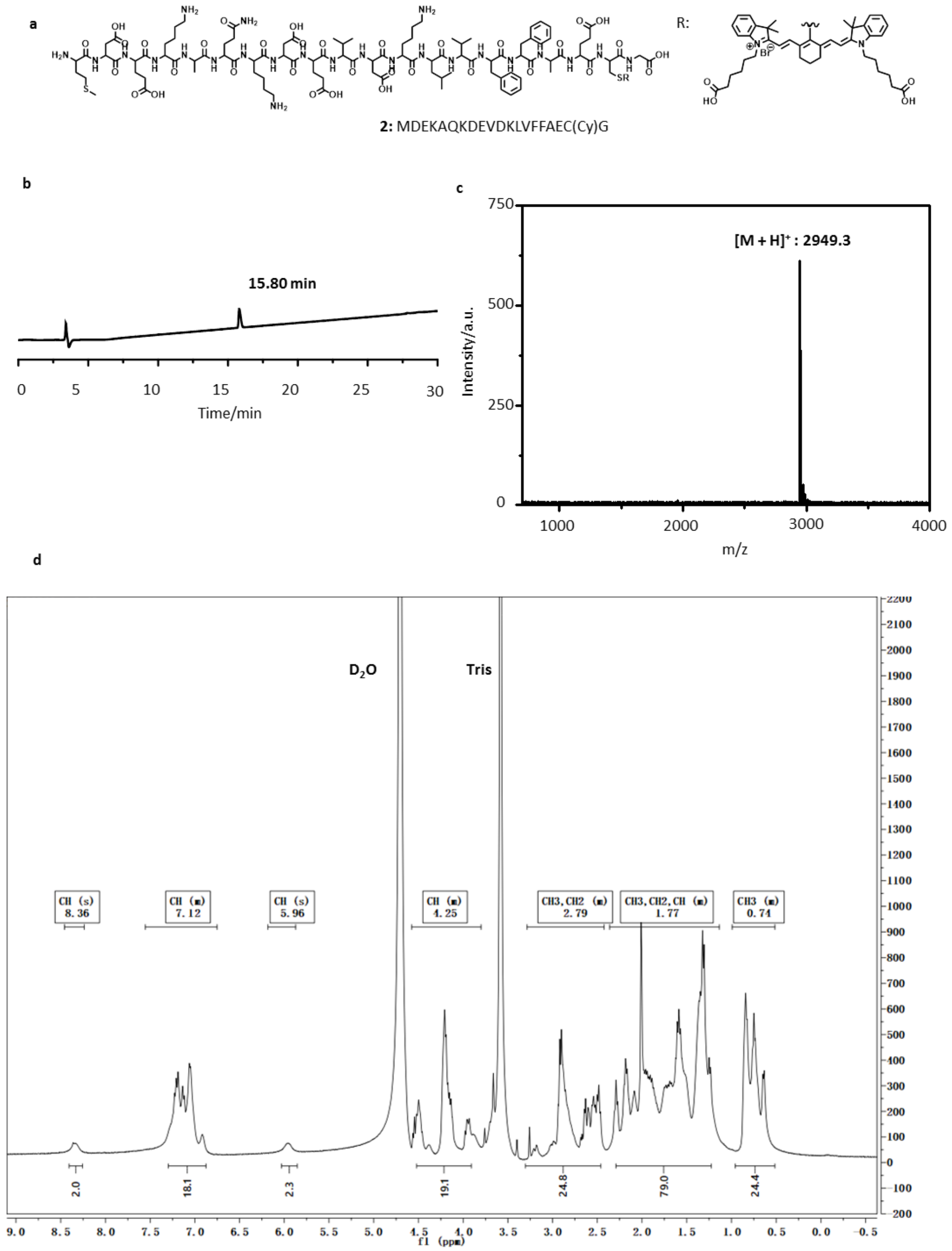

Supplementary Figure 4 | a The structure of molecule **2**; HPLC spectra (**b**) and MALDI-TOF (**c**) molecule **2**; The method of HPLC spectra was as follows: solvent A, 0.1% trifluoroacetic in 100% water; solvent B, 0.1% trifluoroacetic in 100% acetonitrile; 0.01 min, 25% solvent B, 30 min, 90% solvent B. **d**, ^{1}H NMR of molecule **2** in D_2O. ^{1}H NMR (400 MHz, D_2O) δ 8.36 (s, 2H, CH), 7.55-6.75 (m, 18H, CH), 5.96 (s, 2H, CH), 4.58-3.80 (m, 19H, CH), 3.28-2.42 (m, 25H, CH, CH_3), 2.36-1.14 (m, 79H, CH_3, CH_2, CH), 0.99-0.51 (m, 24H, CH_3).

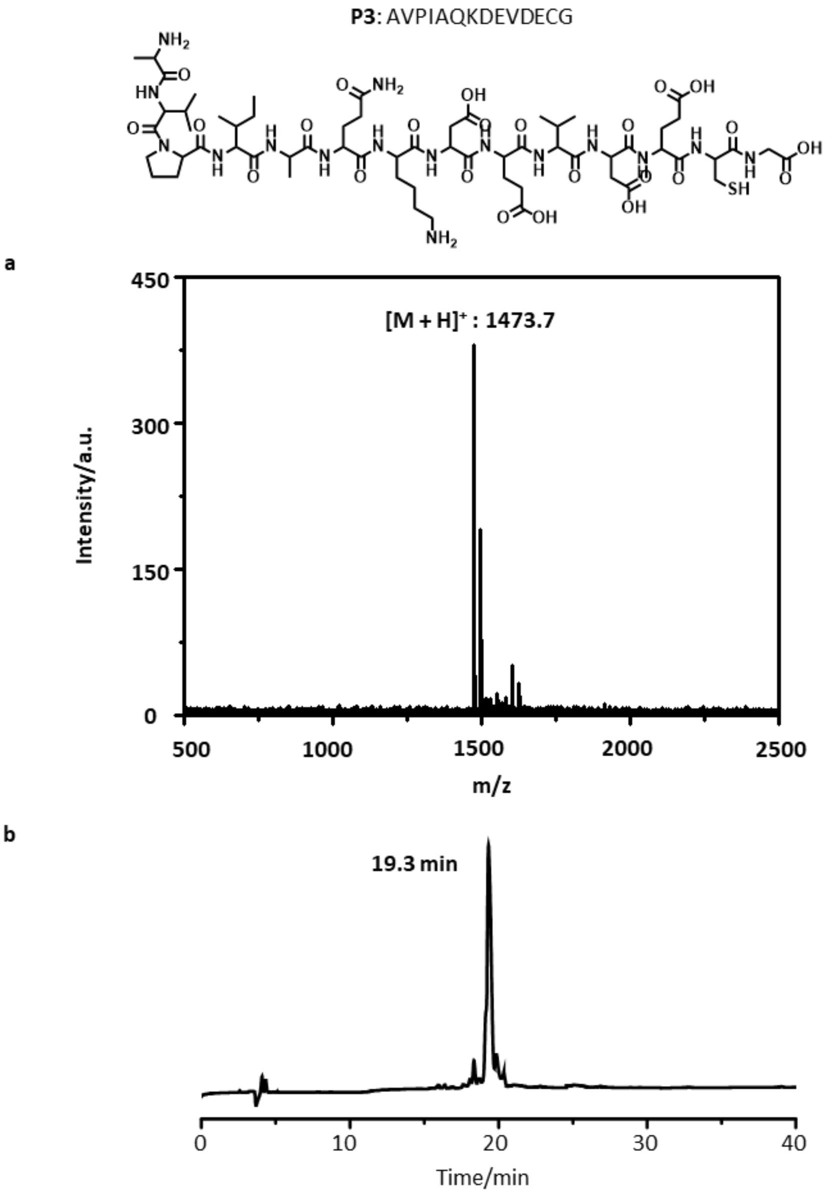

Supplementary Figure 5 | MALDI-TOF (**a**) and HPLC spectra (**b**) of **P3**. The method of HPLC spectra was as follows: solvent A, 0.1% trifluoroacetic in 100% water; solvent B, 0.1% trifluoroacetic in 100% acetonitrile; 5 min, 10% solvent B, 25 min, 50% solvent B, 40 min, 50% solvent B.

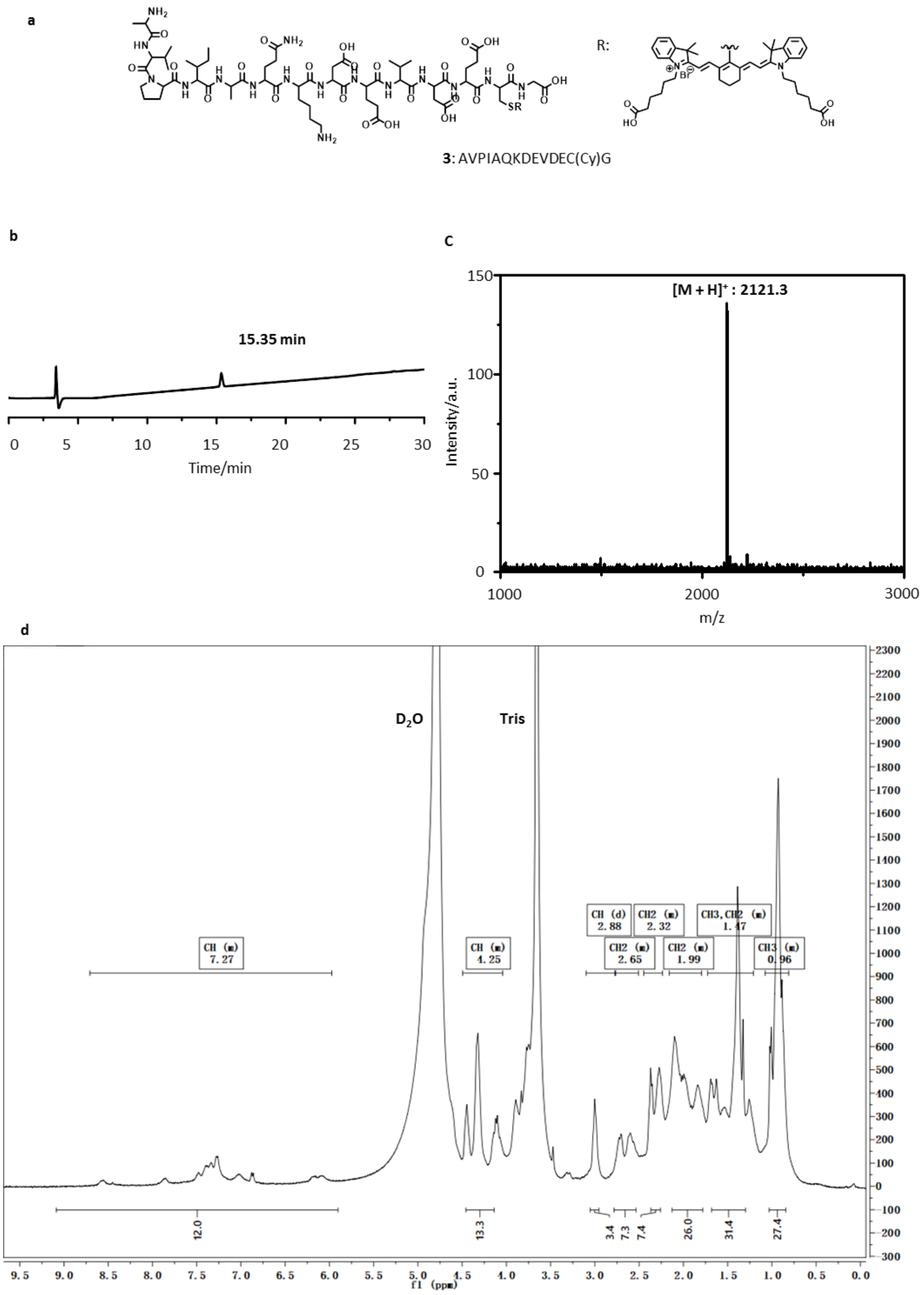

Supplementary Figure 6 | **a** The structure of molecule **3**; HPLC spectra (**b**) and MALDI-TOF (**c**) molecule **3**; The method of HPLC spectra was as follows: solvent A, 0.1% trifluoroacetic in 100% water; solvent B, 0.1% trifluoroacetic in 100% acetonitrile; 0.01 min, 25% solvent B, 30 min, 90% solvent B. **d**, ^{1}H NMR of molecule **3** in D_2O. ^{1}H NMR (400 MHz, D_2O) δ 8.71-5.97 (m, 12H, CH), 4.50-4.04 (m, 13H, CH), 2.88 (d, 3H, CH), 2.78-2.51 (m, 7H, CH_2), 2.45-2.23 (m, 7H, CH_2), 2.16-1.80 (m, 26H, CH_2), 1.72-1.21 (m, 31H, CH_2, CH_3), 1.08-0.81 (m, 27H, CH_3).

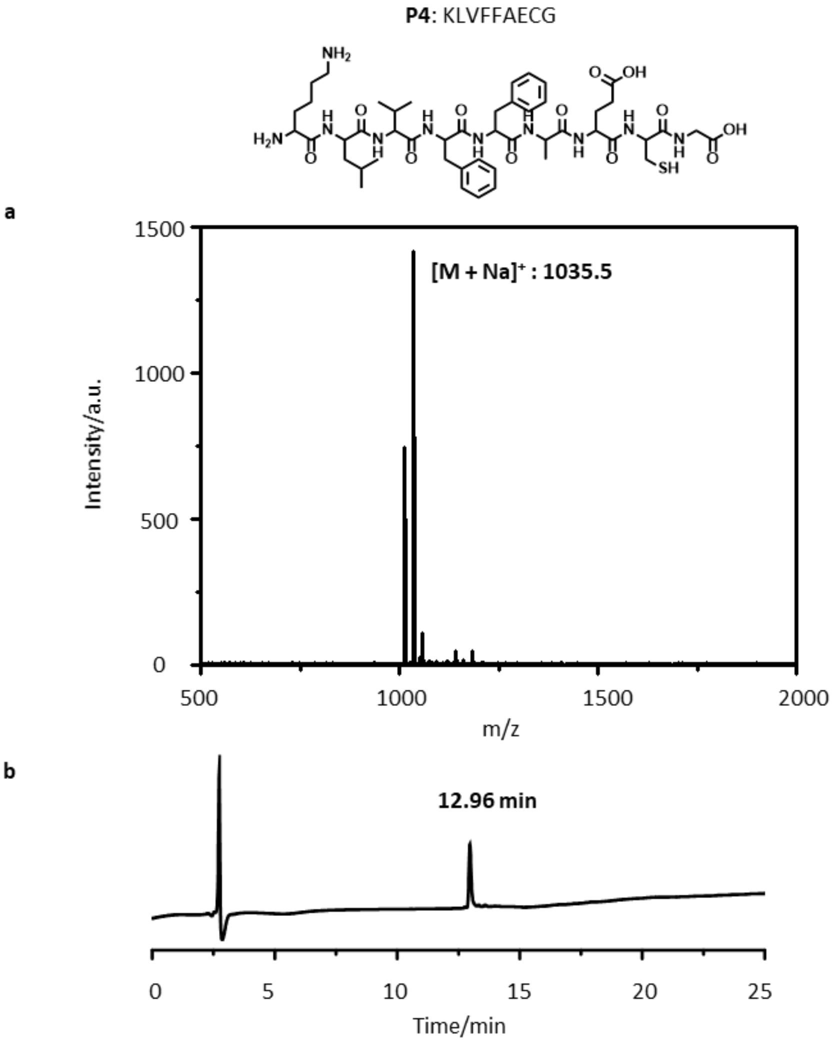

Supplementary Figure 7 | MALDI-TOF (**a**) and HPLC spectra (**b**) of **P4**. The method of HPLC spectra was as follows: solvent A, 0.1% trifluoroacetic in 100% water; solvent B, 0.1% trifluoroacetic in 100% acetonitrile; 0.01 min, 15% solvent B, 25 min, 70% solvent B.

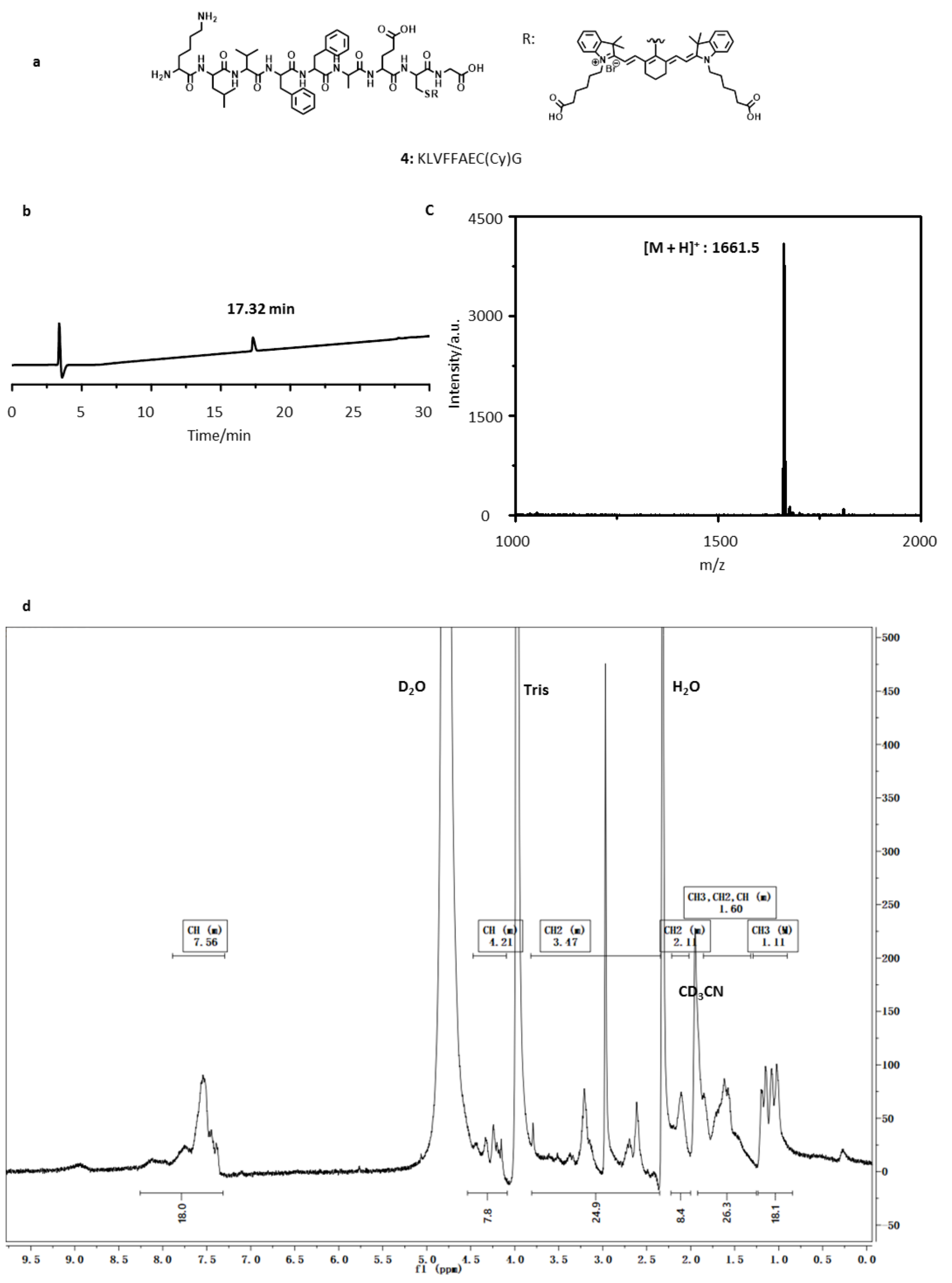

Supplementary Figure 8 | **a** The structure of molecule **4**; HPLC spectra (**b**) and MALDI-TOF (**c**) molecule **4**; The method of HPLC spectra was as follows: solvent A, 0.1% trifluoroacetic in 100% water; solvent B, 0.1% trifluoroacetic in 100% acetonitrile; 0.01 min, 25% solvent B, 30 min, 90% solvent B. **d**, ^{1}H NMR of molecule **4** in 60% D_2O, 40% CD_3CN and 0.1% CF_3COOD. ^{1}H NMR (400 MHz, 60% D_2O, 40% CD_3CN, 0.1% CF_3COOD) δ 7.89-7.30 (m, 18H, CH), 4.47-4.10 (m, 8H, CH), 3.82-2.34 (m, 25H, CH_2), 2.22-2.02 (m, 8H, CH_2), 1.86-1.32 (26H, CH_3, CH_2, CH), 1.29-0.90 (m, 18H, CH_3).

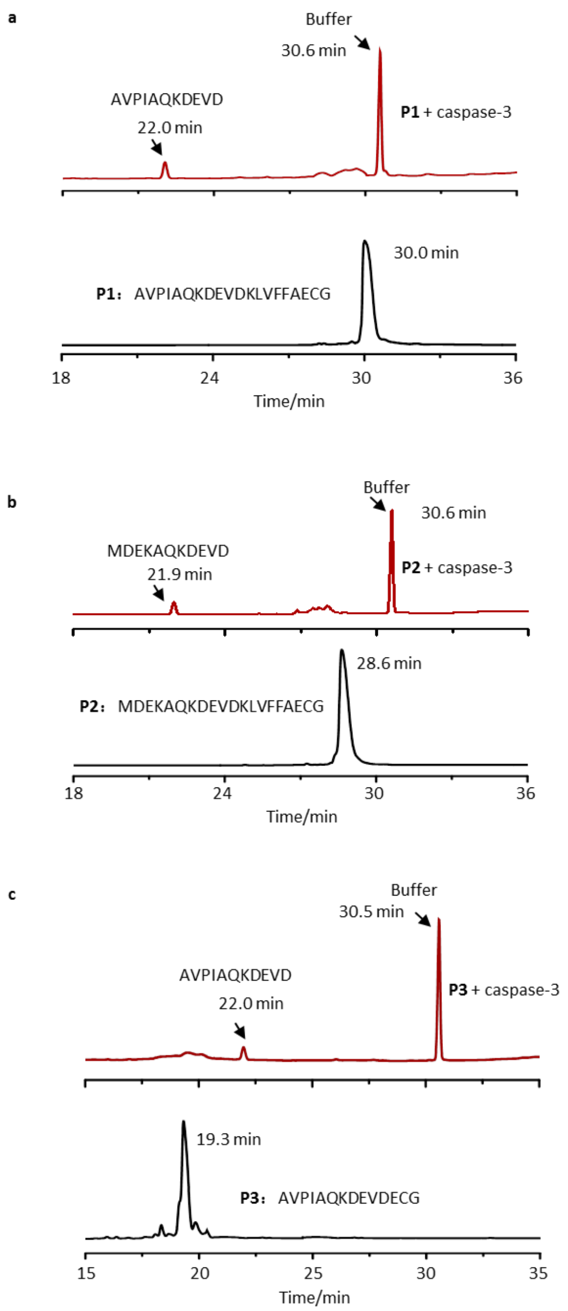

Supplementary Figure 9｜ HPLC traces of **P1**, **P2** and **P3** in water and the mixtures incubated with caspase-3 (substrate-to-enzyme ratio of 1 μM per U) for 12 h at 37 °C in HEPES (4-(2-hydroxyethyl) -1-piperazineethanesulfonic acid) buffer (50 Mm, pH 7.4). **a**, **P1** (black) and mixture with **P1** and caspase-3 (red). **b**, **P2** (black) and mixture with **P2** and caspase-3 (red). **c**, **P3** (black) and mixture with **P3** and caspase-3 (red).

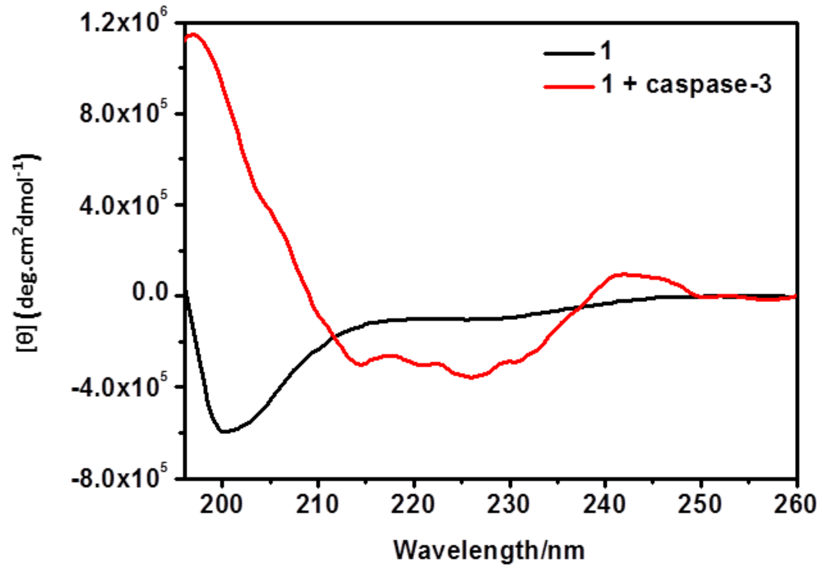

Supplementary Figure 10 | CD spectra of self-assembled β-sheet nanofibrils of molecule 1 after treatment with caspase-3 (substrate-to-enzyme ratio of 1 μM per U) in HEPES buffer (50 mM, pH 7.4) for 2 h.

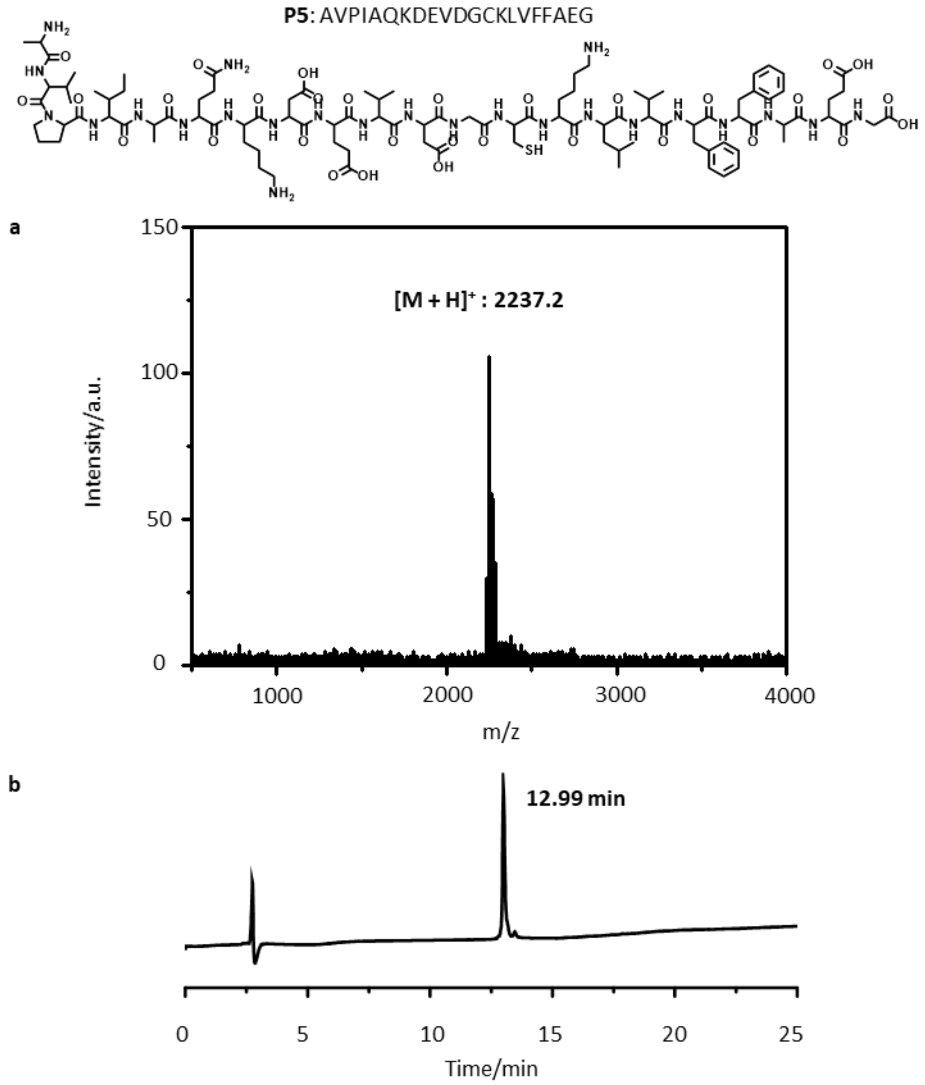

Supplementary Figure 11 | MALDI-TOF (**a**) and HPLC spectra (**b**) of **P5**. The method of HPLC spectra was as follows: solvent A, 0.1% trifluoroacetic in 100% water; solvent B, 0.1% trifluoroacetic in 100% acetonitrile; 0.01 min, 15% solvent B, 25 min, 70% solvent B.

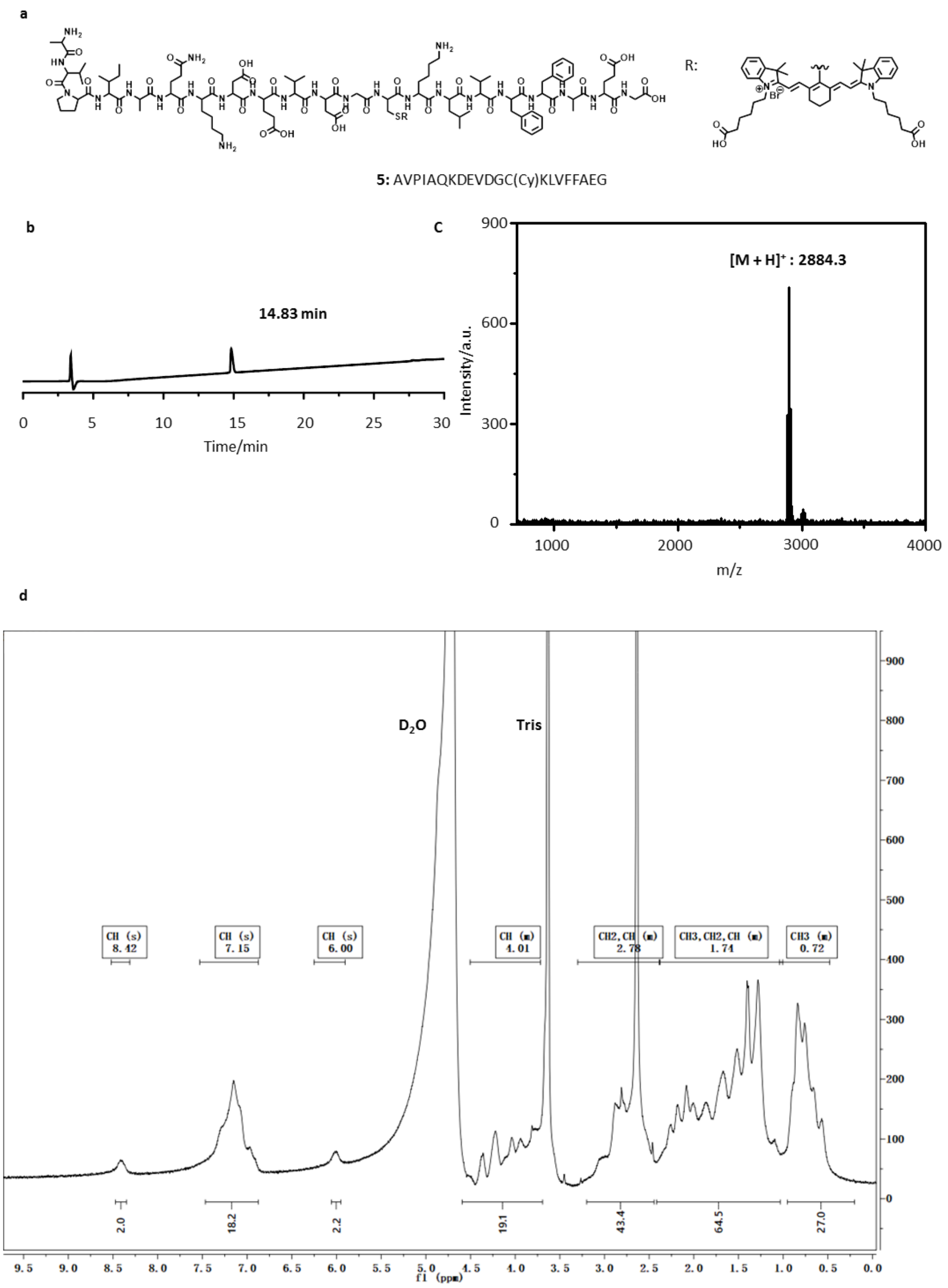

Supplementary Figure 12 | **a** The structure of molecule **5**; HPLC spectra (**b**) and MALDI-TOF (**c**) molecule **5**; The method of HPLC spectra was as follows: solvent A, 0.1% trifluoroacetic in 100% water; solvent B, 0.1% trifluoroacetic in 100% acetonitrile; 0.01 min, 25% solvent B, 30 min, 90% solvent B. **d**, ^{1}H NMR of molecule **5** in D_2O. ^{1}H NMR (400 MHz, D_2O) δ 8.42 (s, 2H, CH), 7.15(s, 18H, CH), 6.0 (s, 2H, CH), 4.50-3.78 (m, 19H, CH), 3.30-2.39 (m, 43H, CH_2, CH), 2.38-1.01 (m, 65H, CH_3, CH_2, CH), 1.04-0.48 (m, 27H, CH_3).

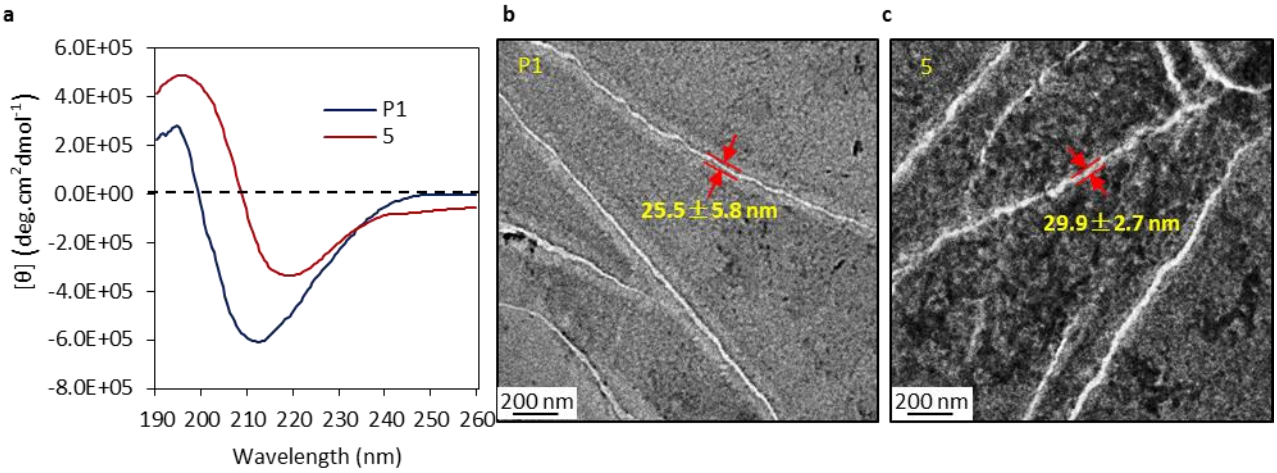

Supplementary Figure 13 | **a,** CD spectral of **P1** (1 μM) and **5** in deionized water. **b** and **c,** TEM of molecules **P1** (50 μM) and **5** (50 μM) in deionized solution.

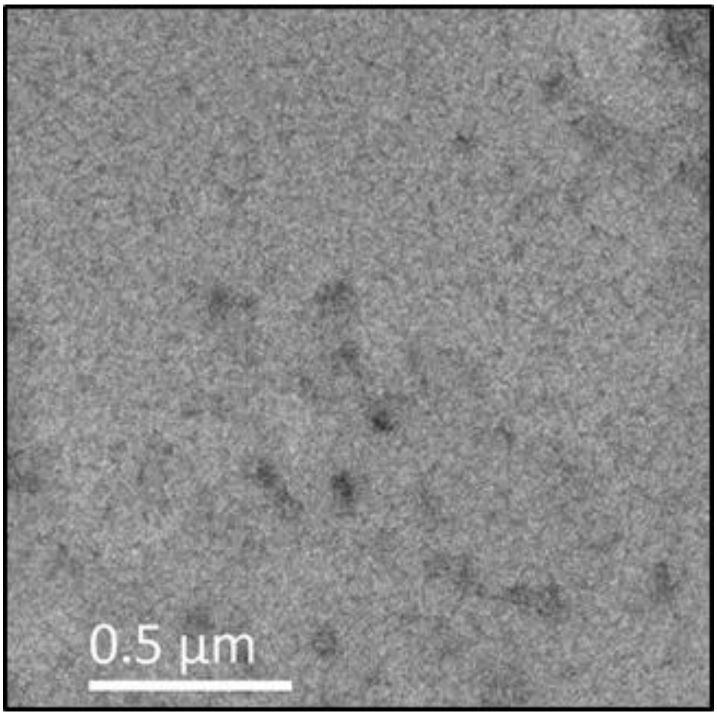

Supplementary Figure 14 | TEM image of caspase-3 in HEPES buffer (50 mM, pH 7.4).

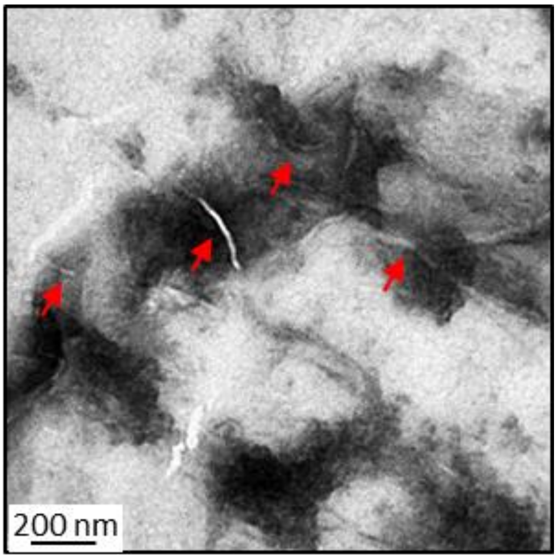

Supplementary Figure 15 | TEM imaging of caspase-3 treated with molecules **2** (substrate-to-enzyme ratio of 1 μM per U) in HEPES buffer (50 mM, pH 7.4) at 37 °C for 2 h. The TEM samples were prepared by placing nanofibrils droplets on copper grids for 5 min and the excess droplets were removed. Finally, the samples were stained by uranyl acetate for 2 min before the TEM studies.

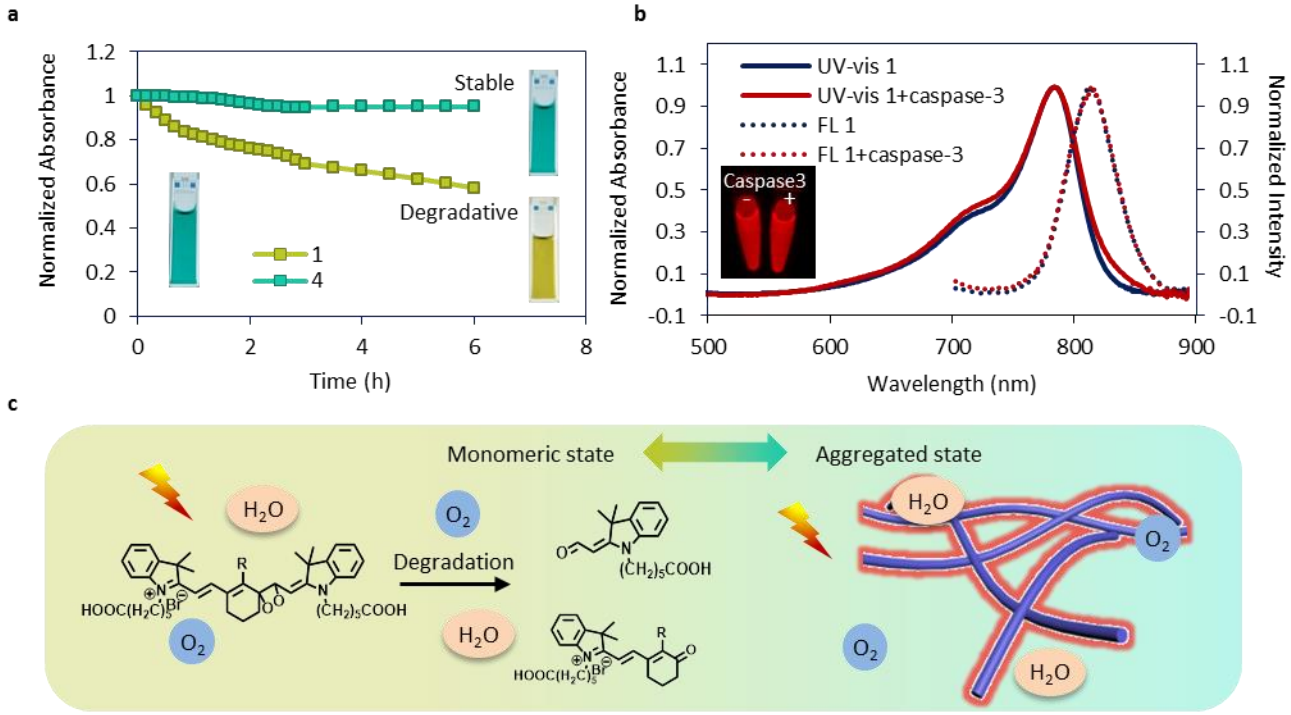

Supplementary Figure 16 | **a**, Photostability evaluation of **1** and **4** under a xenon flash lamp. Molecules **1** and **4** (5 μM) were dissolved in PBS buffer (10 mM, pH 7.4) and the absorbance at 780 nm were recorded for 6 h at r.t. **b**, The UV-vis and fluorescence spectra of molecule **1** (5 μM) before or after addition of caspase-3 (substrate-to-enzyme ratio of 1 μM per U) in HEPES buffer (50 mM, pH 7.4). Inset: Photographs of the color changing at the emission λ_{em}=820 nm of molecule **1** before/after addition of caspase-3 (substrate-to-enzyme ratio of 1 μM per U). **c**, Schematic illustration of the photostability differences in monomeric state and aggregated state.

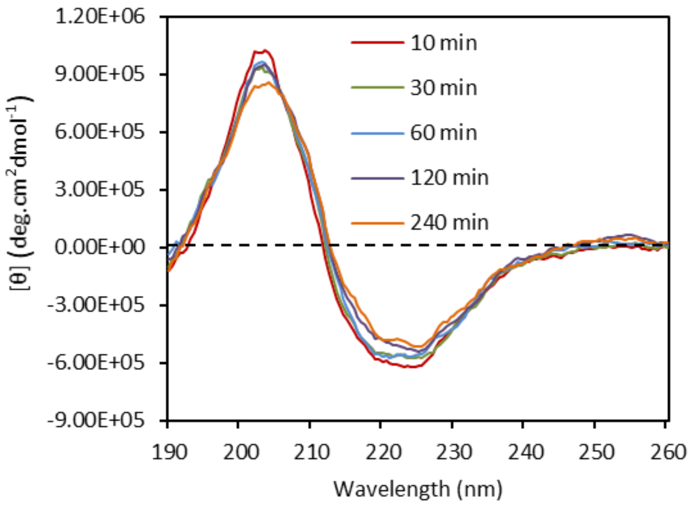

Supplementary Figure 17 | Time-depended CD spectra of the molecule **4** (5 μM) in deionized water.

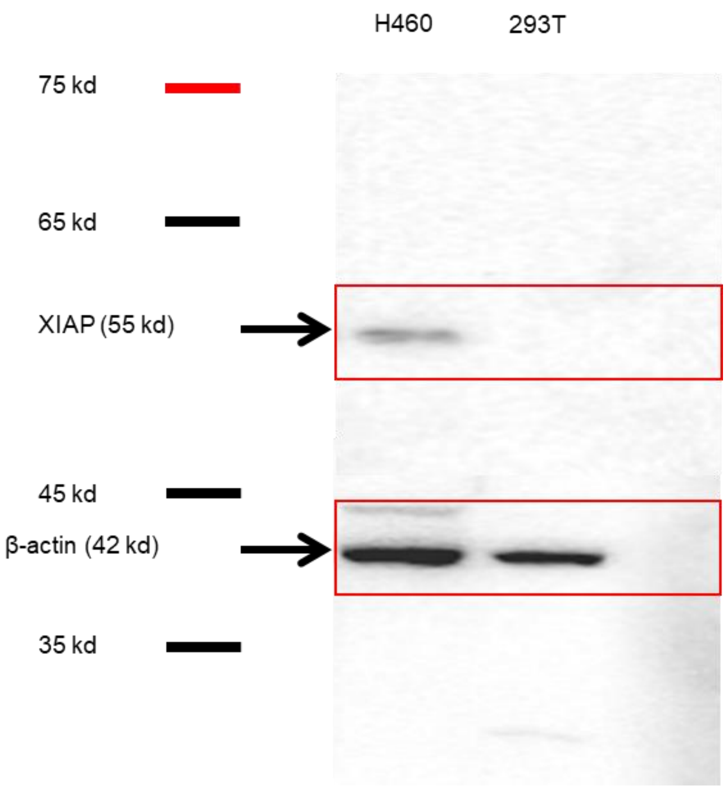

Supplementary Figure 18 | Uncropped version of XIAP western blot shown in Figure 2a.

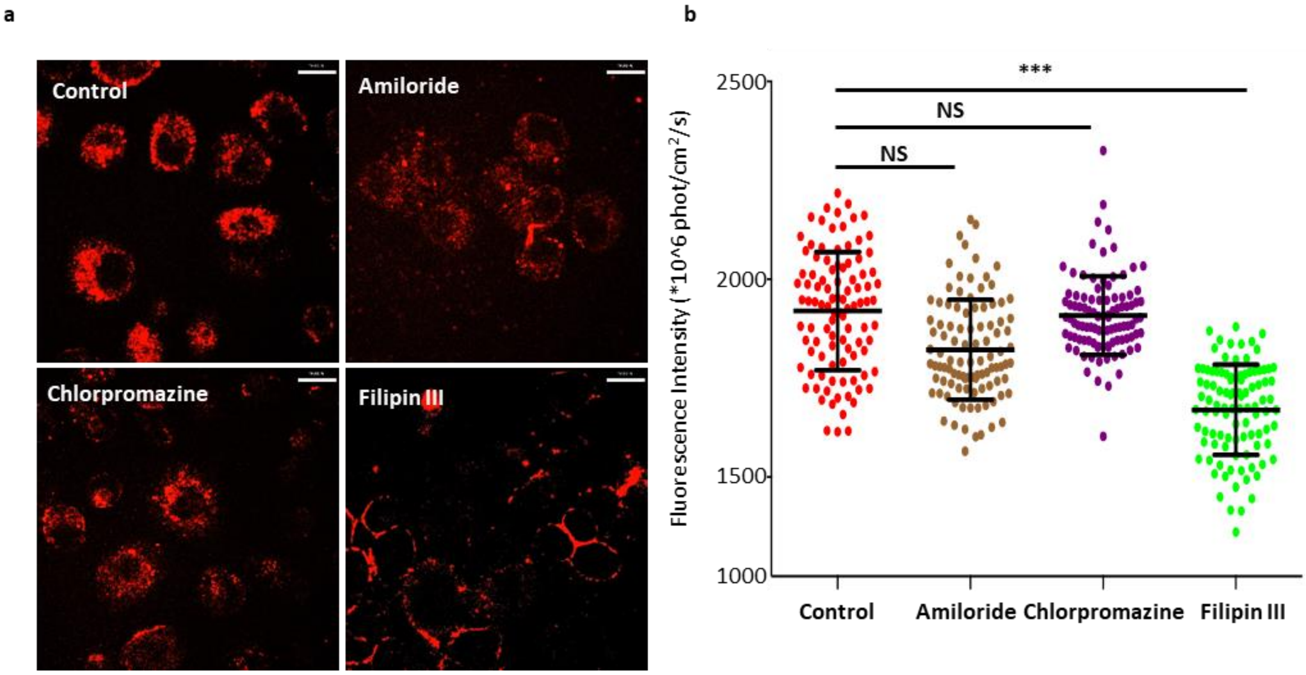

Supplementary Figure 19 | a) Confocal images showing cellular uptake of molecule 1; b) Statistical analysis of average fluorescence intensities in the cytoplasm of H460 cells from confocal images. Data are presented as the mean ± s.d. (n=100). n.s. means not significant, *** $p < 0.001$, p values were performed with one-way ANOVA followed by post hoc Tukey's test for the indicated comparison.

P6: AVPIAQKGGGRRRRRRRRGC

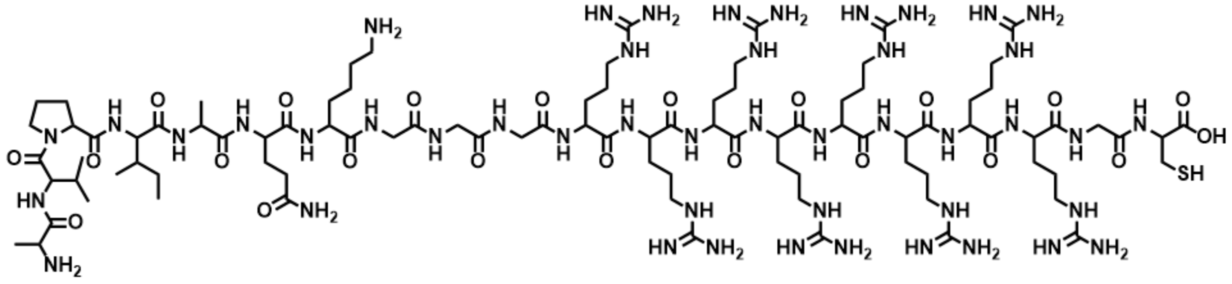

a

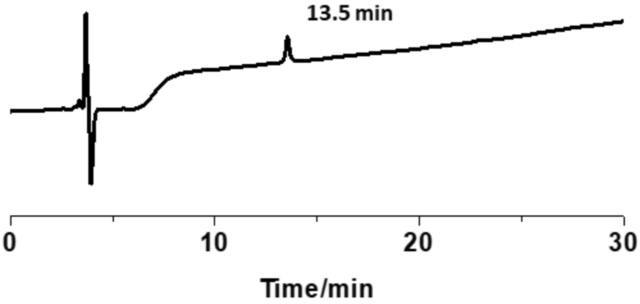

b

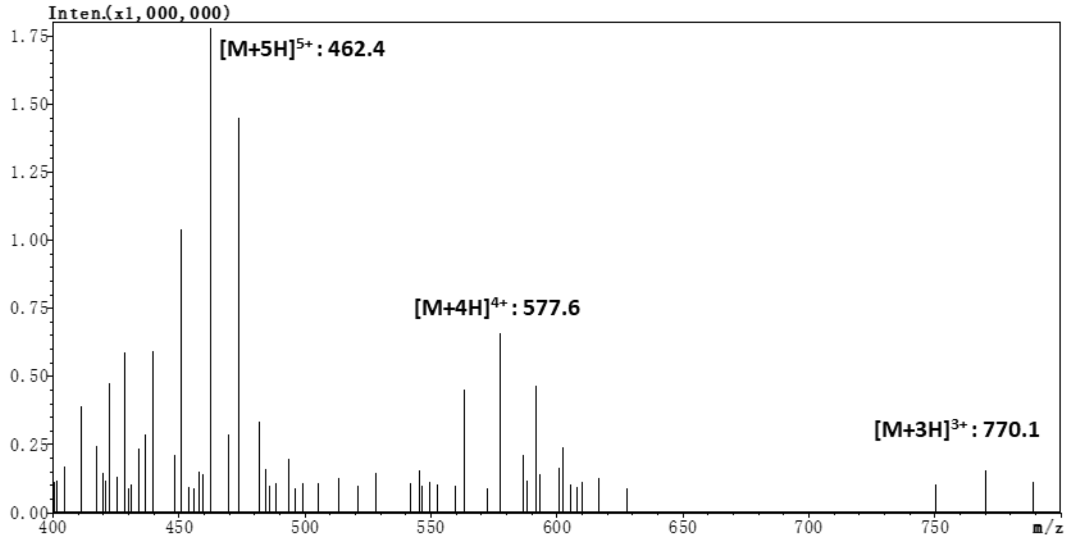

Supplementary Figure 20 | HPLC spectra (**a**) and MS-ESI (**b**) spectra of **P6**. The method of HPLC spectra was as follows: solvent A, 0.1% trifluoroacetic in 100% water; solvent B, 0.1% trifluoroacetic in 100% acetonitrile; 0.01 min, 10% solvent B, 30 min, 40% solvent B.

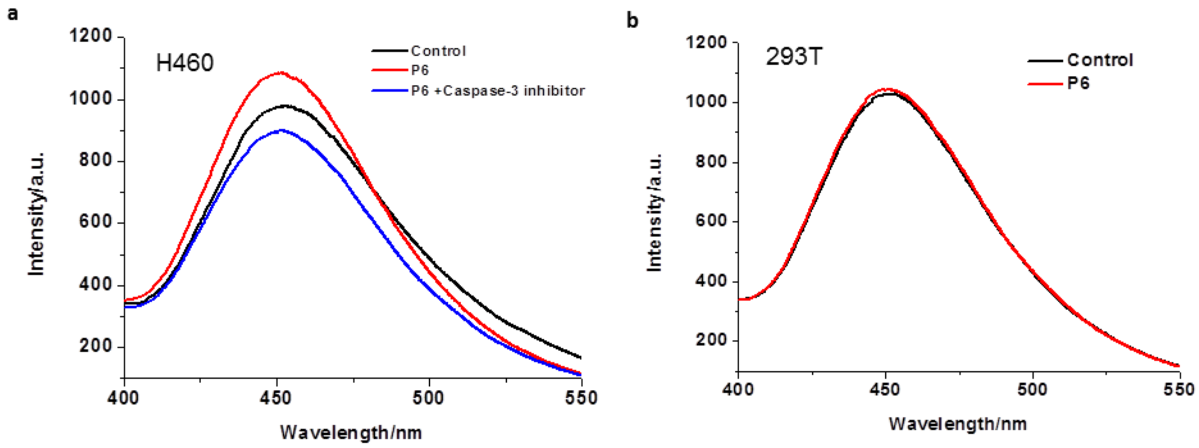

Supplementary Figure 21 | The fluorescence intensity of caspase substrate DEVD-MCA in H460 cells (a) and 293T cells (b).

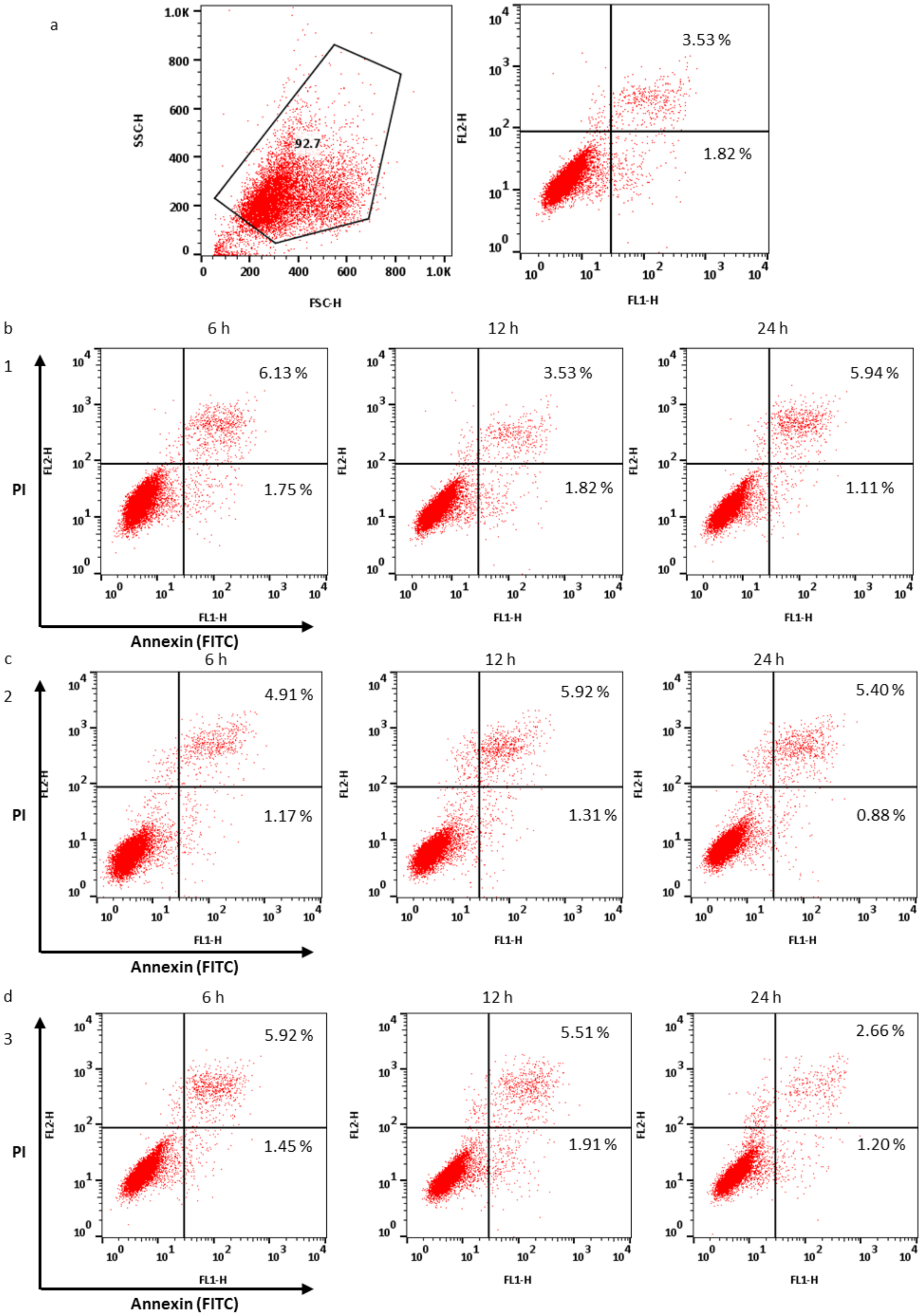

Supplementary Figure 22 | a. Gate analysis of apoptotic H460 cells. Flow cytometry of molecules **1** (**b**), **2**(**c**) and **3** (**d**) at 6 h, 12 h and 24 h.

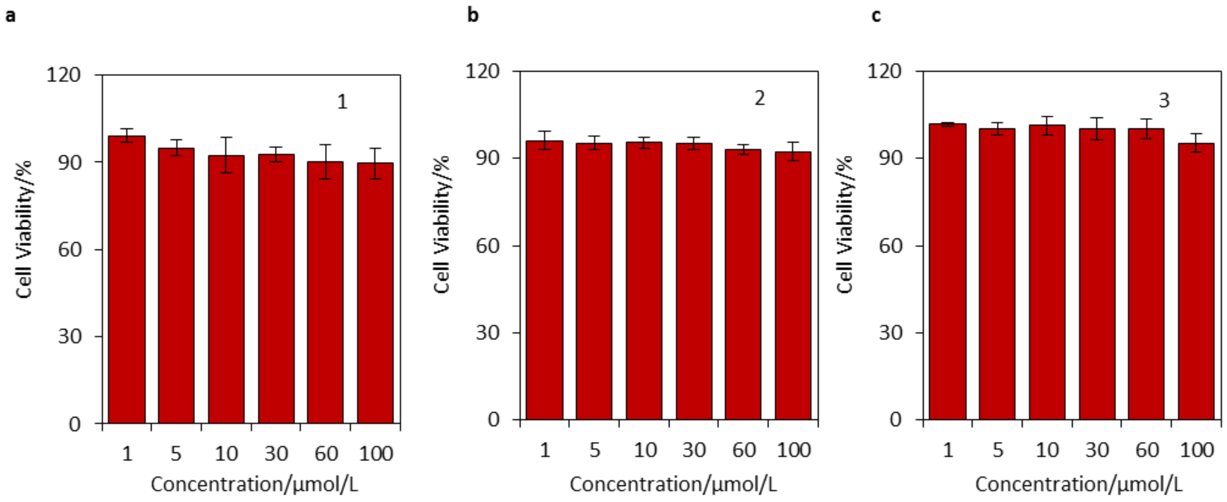

Supplementary Figure 23｜ Viability of non-small cell lung cancer (NSCLC) cells (H460) upon treatment with molecules **1** (a) ,**2** (b) and **3** (c) at different concentrations.

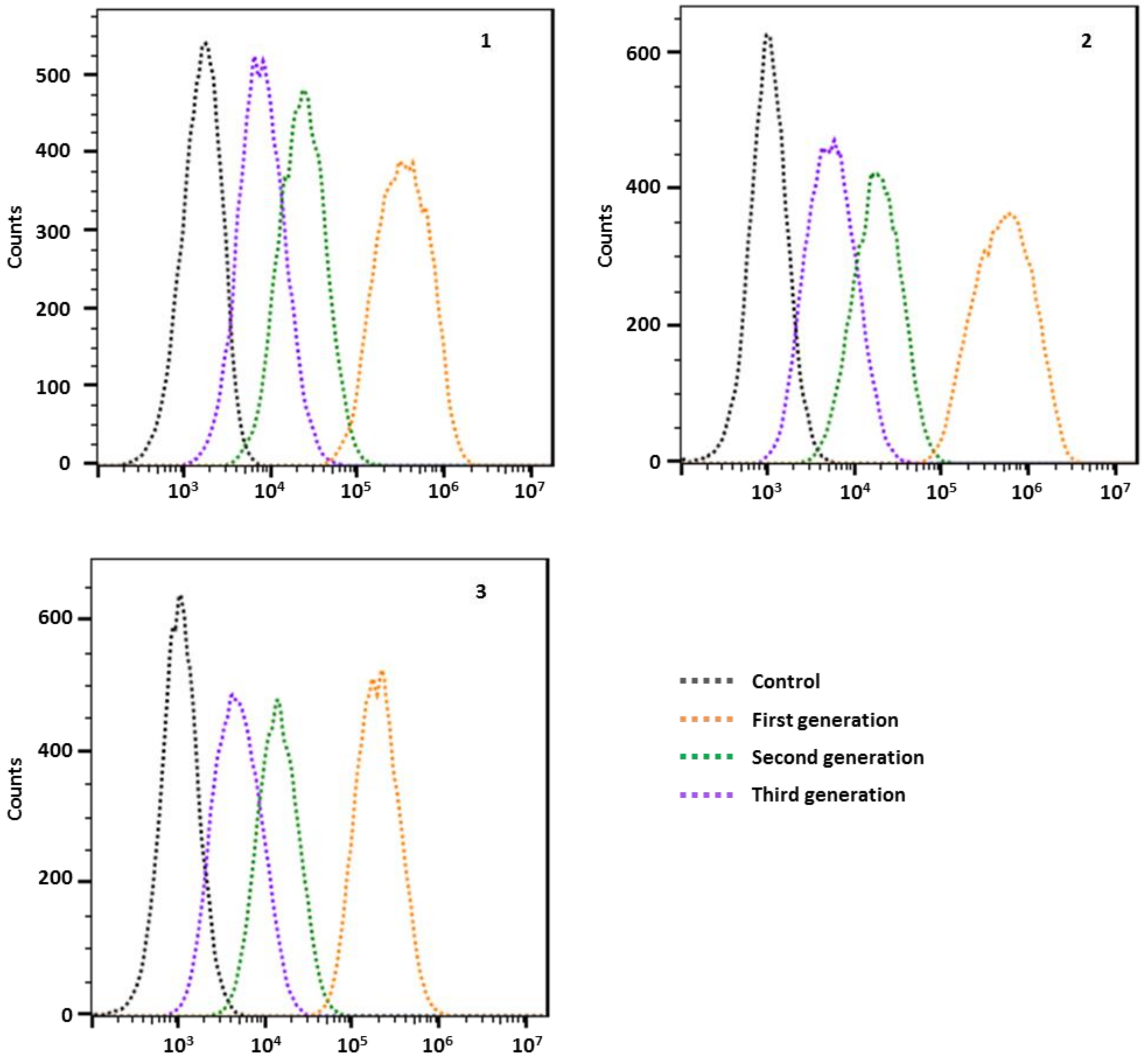

Supplementary Figure 24 | Flow cytometry histograms of H460 cells treated with molecule 1, 2 and 3 (50 μM) at 37 °C for 2 h, respectively, and then subcultured for designated generations (a 24 h interval). The untreated H460 cells were used as the control group.

P1-NBD: AVPIAQKDEVDK(NBD)LVFFAECG

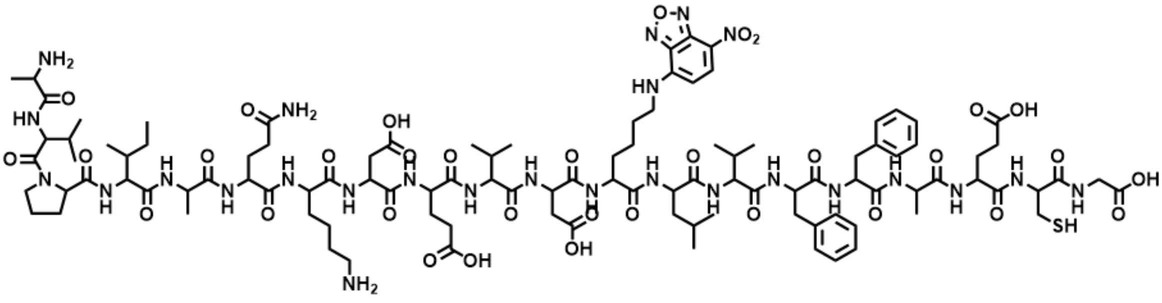

a

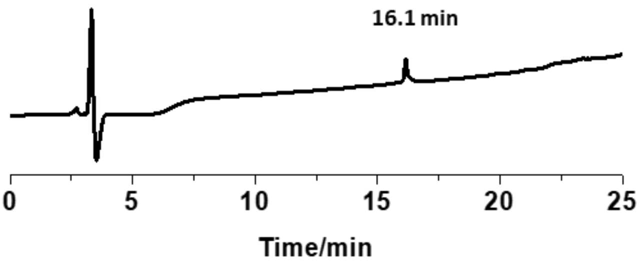

b

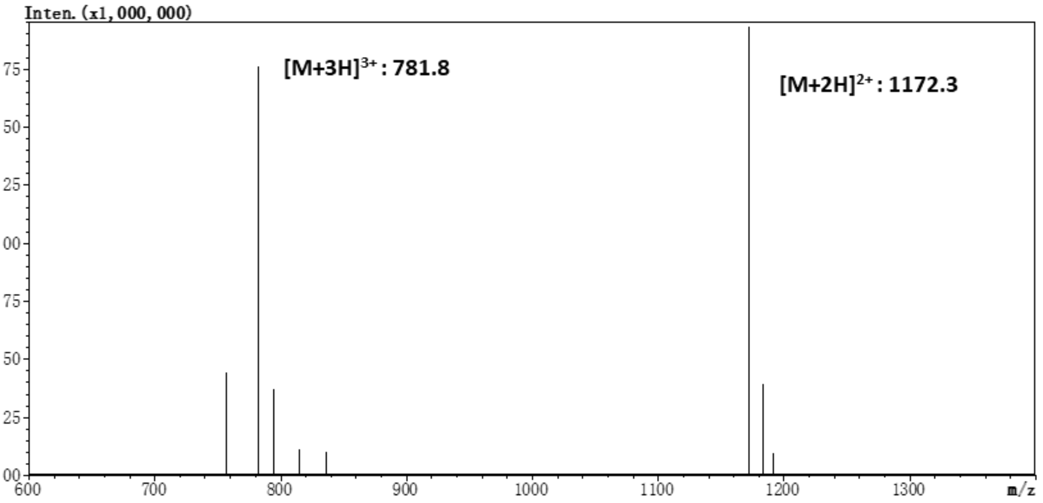

Supplementary Figure 25丨 HPLC spectra (**a**) and MS-ESI (**b**) spectra of **P1-NBD**. The method of HPLC spectra was as follows: solvent A, 0.1% trifluoroacetic in 100% water; solvent B, 0.1% trifluoroacetic in 100% acetonitrile; 0.01 min, 20% solvent B, 25 min, 80% solvent B.

1-NBD: AVPIAQKDEVDK(NBD)LVFFAEC(Cy)G

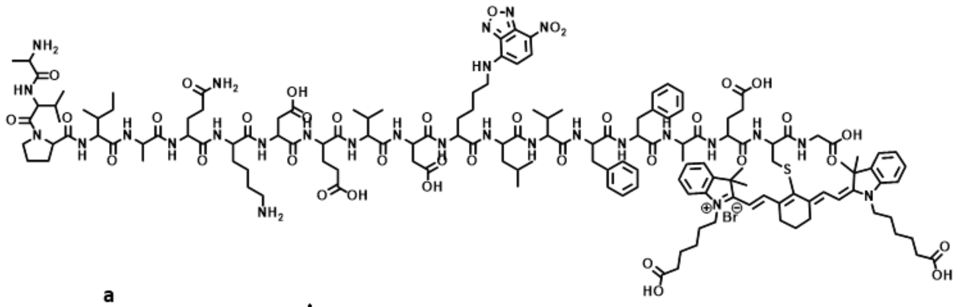

a

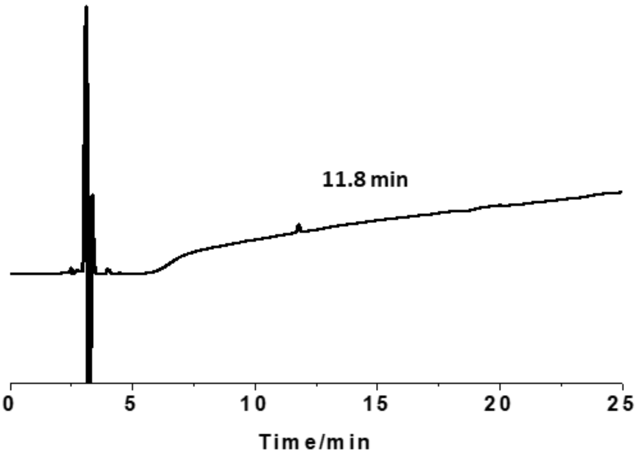

b

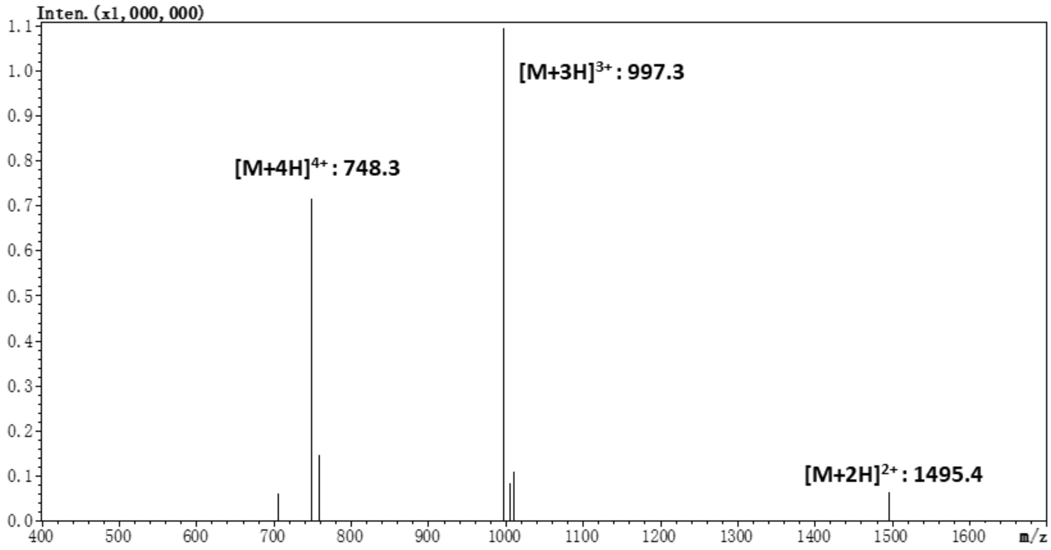

Supplementary Figure 26 | HPLC spectra (**a**) and MS-ESI (**b**) spectra of **1-NBD**. The method of HPLC spectra was as follows: solvent A, 0.1% trifluoroacetic in 100% water; solvent B, 0.1% trifluoroacetic in 100% acetonitrile; 0.01 min, 40% solvent B, 20 min, 90% solvent B; 25 min, 100% solvent B.

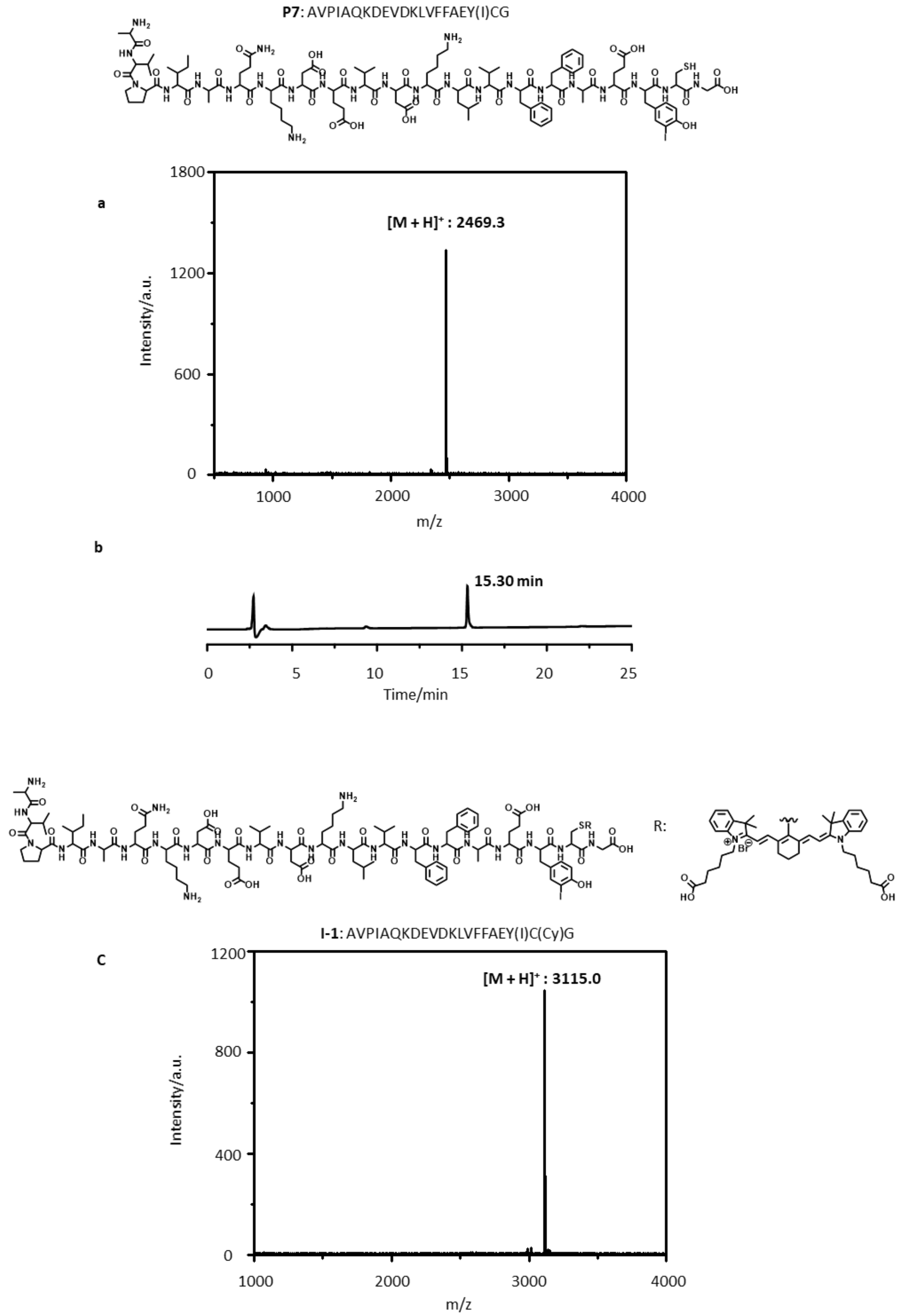

Supplementary Figure 27 | MALDI-TOF (**a**) and HPLC spectra (**b**) of **P7**; **c**, MALDI-TOF of **I-1**. The method of HPLC spectra was as follows: solvent A, 0.1% trifluoroacetic in 100% water; solvent B, 0.1% trifluoroacetic in 100% acetonitrile; 0.01 min, 15% solvent B, 25 min, 70% solvent B.

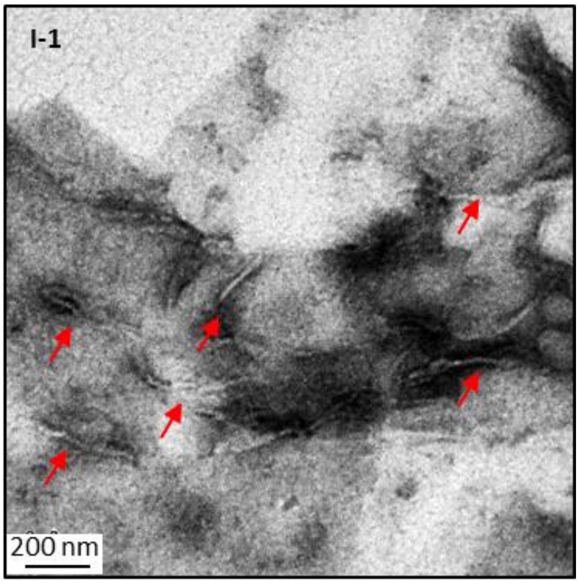

Supplementary Figure 28 | TEM images of molecule **I-1** (50 μM) treated with caspase-3 (5 x 10^{-3} U) in HEPES buffer (50 mM, pH 7.4) at 37 °C for 2 h. The red arrows pointed out the nanofibrils structures.

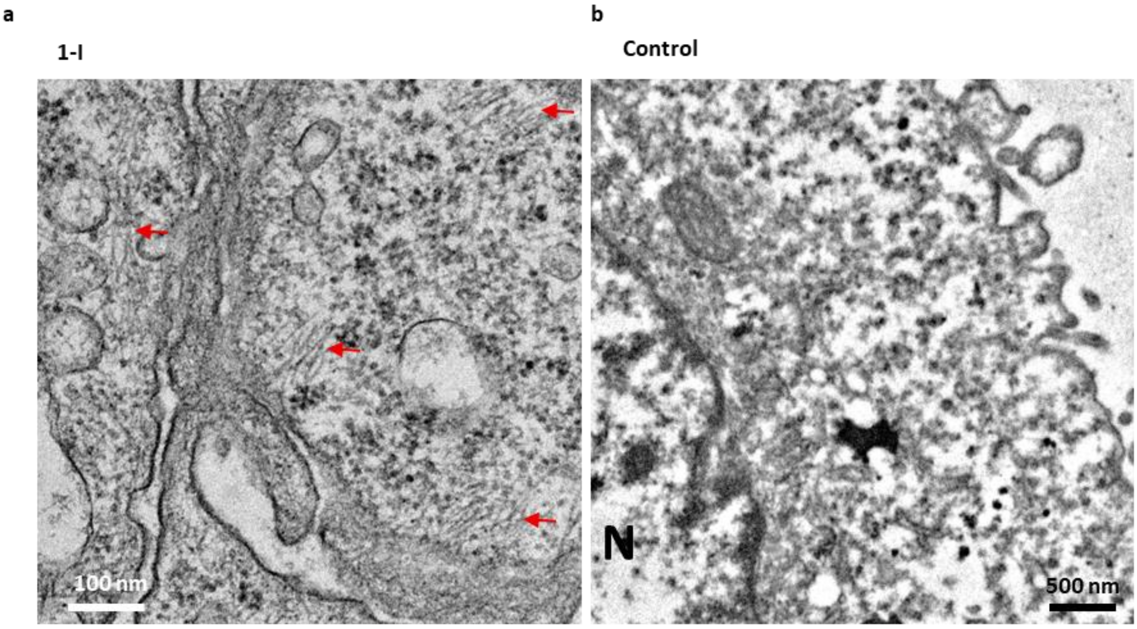

Supplementary Figure 29 | **a**, Bio-TEM images of H460 cells ultrathin sections which were treated with molecule **I-1** (50 μM) for 12 h. **b,** Bio-TEM images of the ultrathin sections which weren't treated with molecule **I-1.** N labeled for nucleus of H460 cell and red arrows pointed the nanofibrils formed from molecule **I-1**.

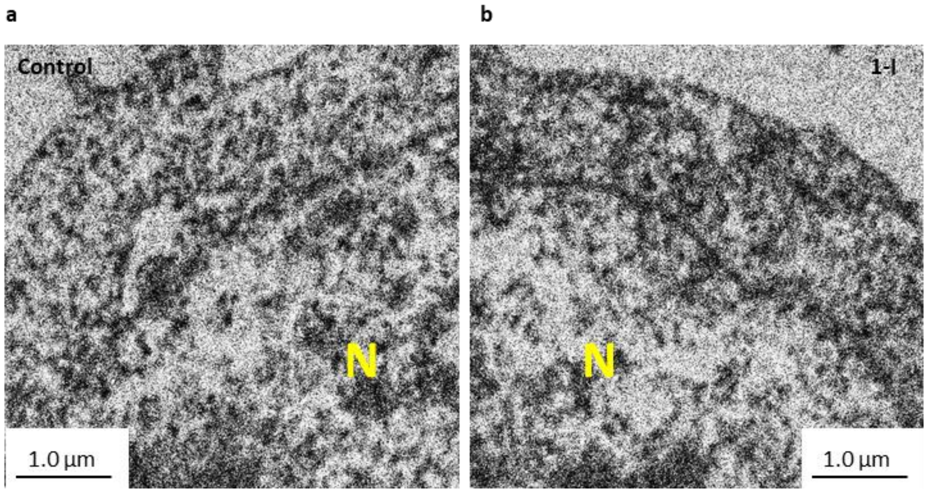

Supplementary Figure 30｜ **a**, Bio-TEM images of the ultrathin sections of untreated with molecule **I-1. b,** Bio-TEM images of ultrathin section of molecule **I-1** (50 µM) treated 293T cells up to 12 h. N labeled for nucleus of 293T cell.

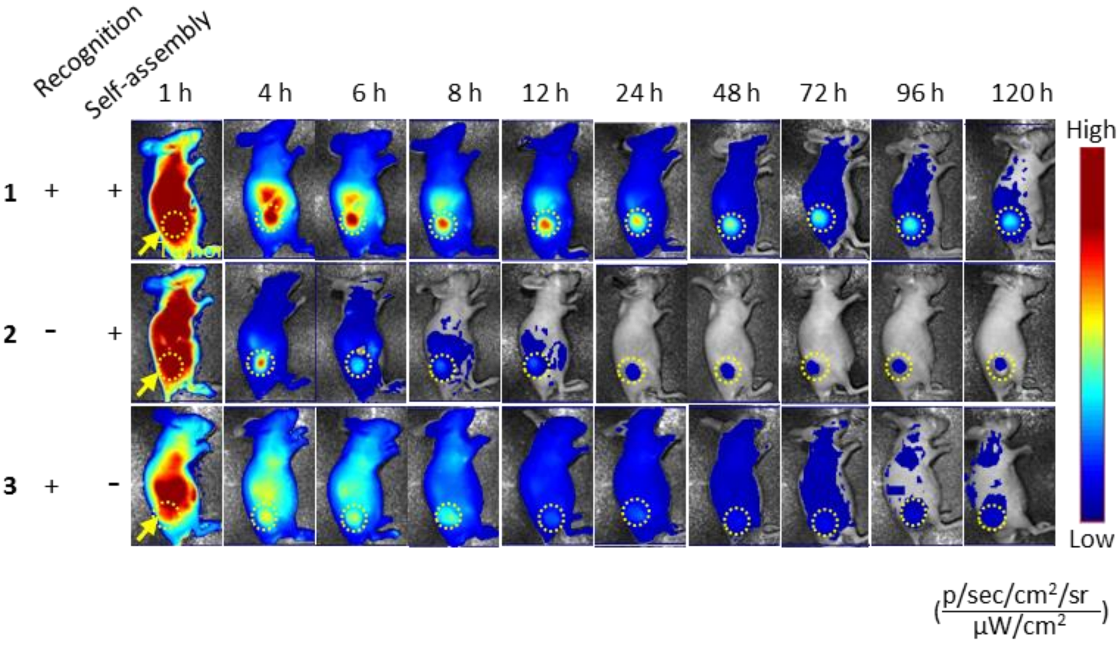

Supplementary Figure 31 | Representative NIR fluorescence images of H460 tumour-bearing mice after intravenous administration with molecules **1**, **2** and **3** (14 mg/kg, n = 3). Images were acquired at 1 h, 4 h, 12 h, 24 h 48 h, 72 h, 96 h and 120 h post injection (p.i.). The yellow circles marked the same size of the tumour region.

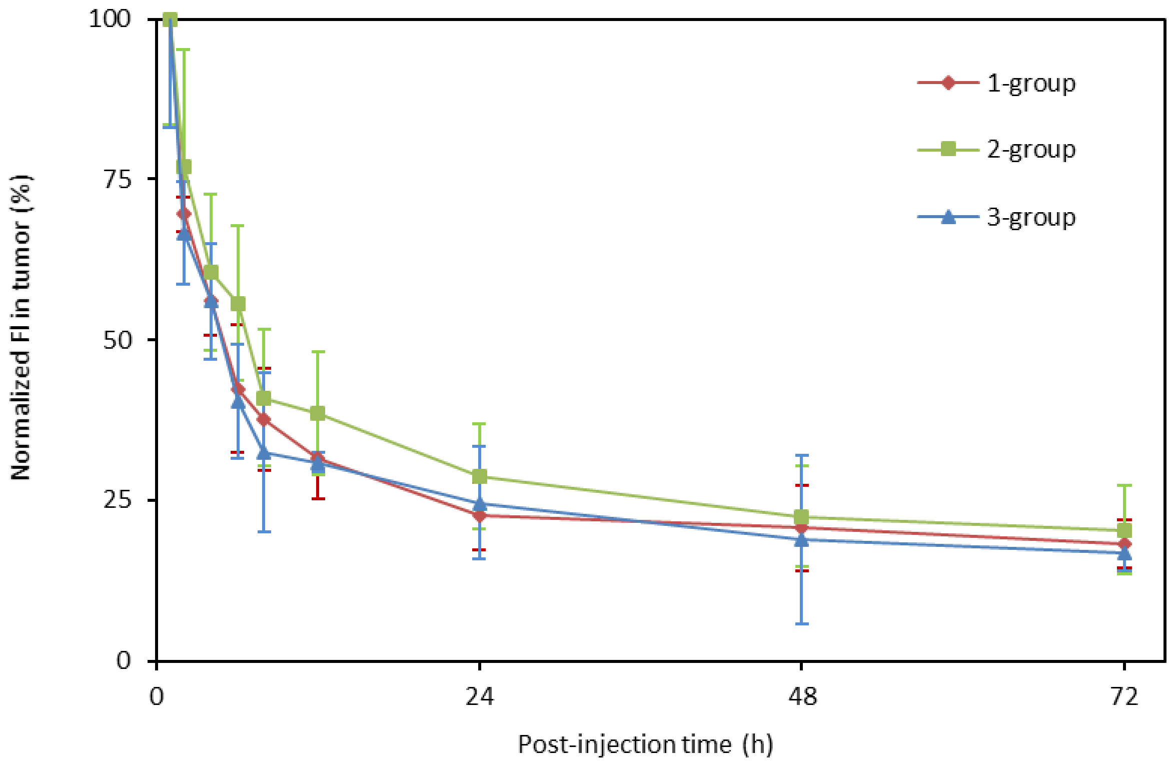

Supplementary Figure 32 | Representative NIR fluorescence images of H460 tumour-bearing mice after intravenous administrated with molecules 1 (14 mg/kg, n = 3) in three groups (In order to test the repeatability of the results, we chose different batches of mice to repeat trials. They were named 1-group, 2-group and 3-group, respectively.). Images were acquired at 1 h, 4 h, 12 h, 24 h 48 h and 72 h post injection (p.i.). All data were obtained by normalized fluorescence intensity in tumour. Data are presented as the mean ± s.d. (n=3).

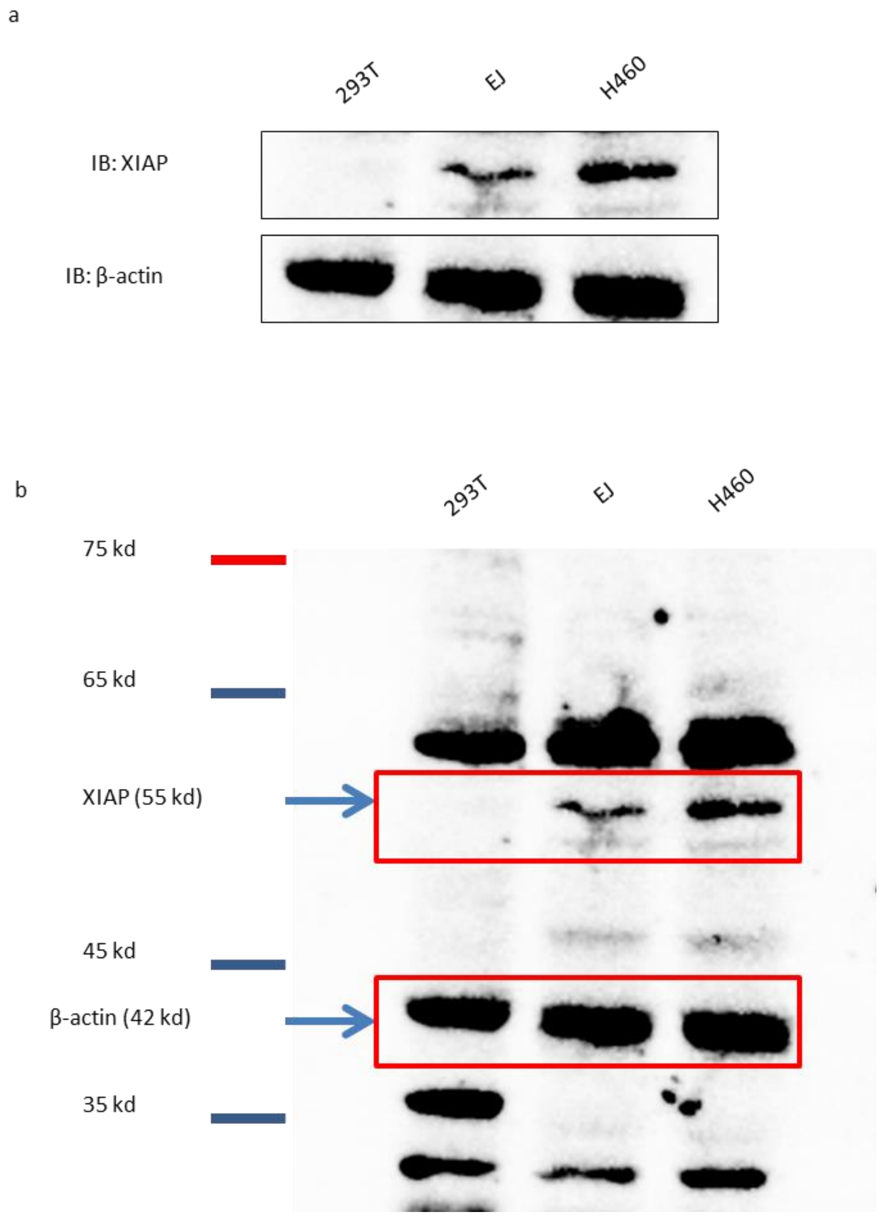

Supplementary Figure 33 | **a,** Western blots of XIAP expression in 293T, EJ and H460 cells. **b,** Uncropped version of XIAP western blot shown in (a).

a

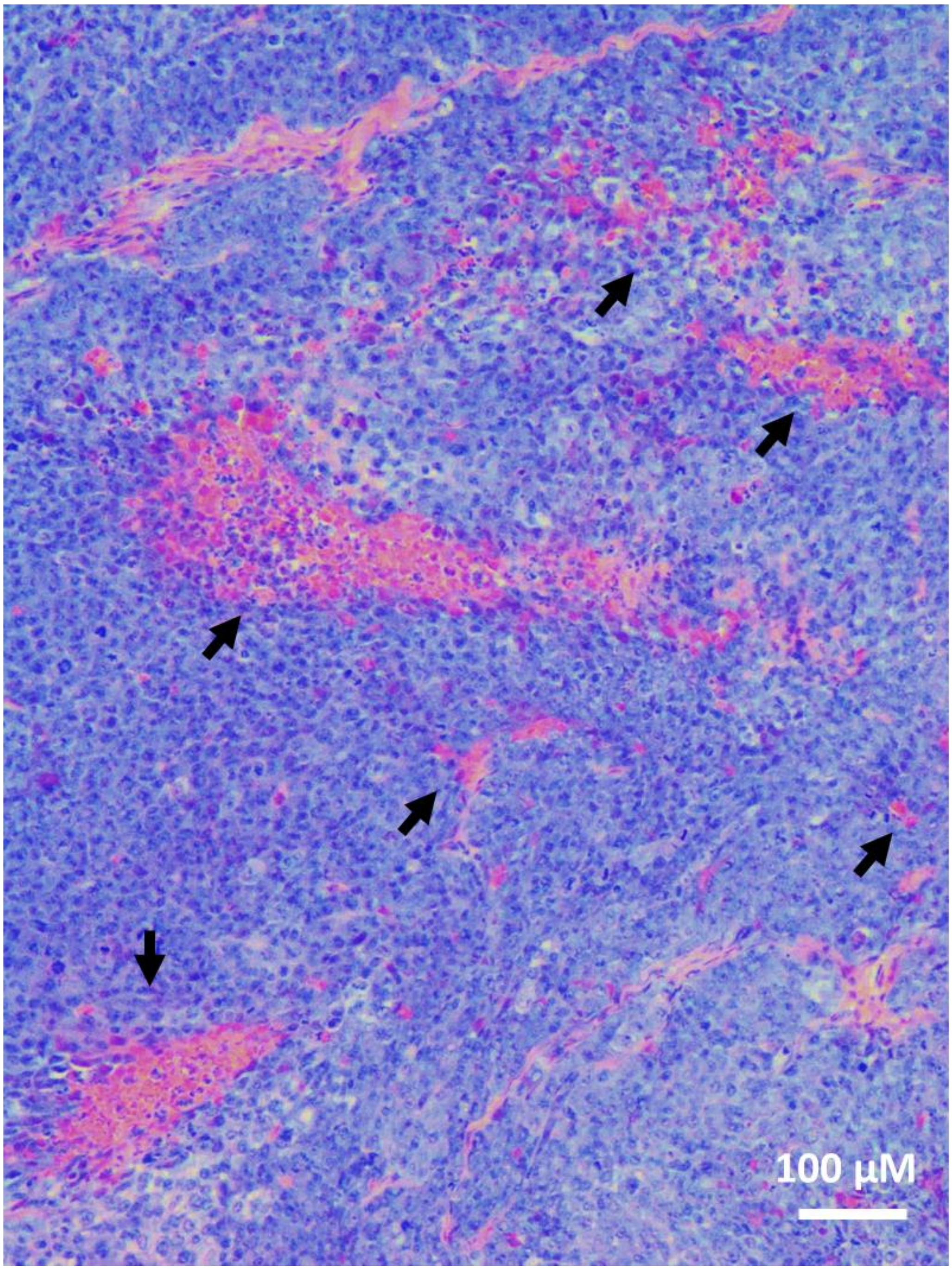

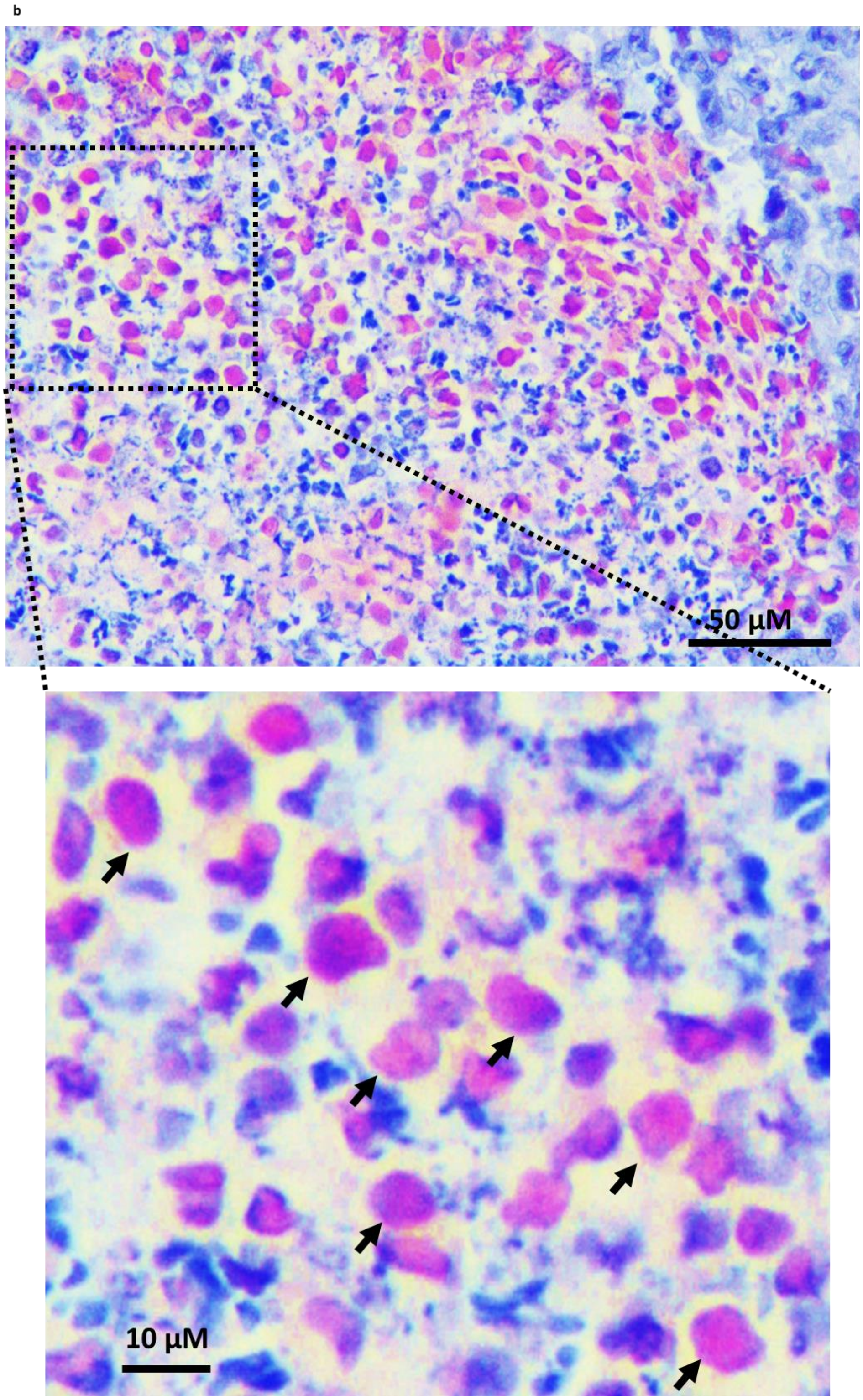

Supplementary Figure 34 | **a.** Light microscopy images of Congo red-stained sections of the tumours. The tumours were removed after 24 h treated with or without molecule **1** (14 mg/kg). The black arrow indicated the nanofibers. **b**. The magnified images of Congo red-stained sections of the tumours. From the magnified image, it can be observed that the stained area is in the cell.

a

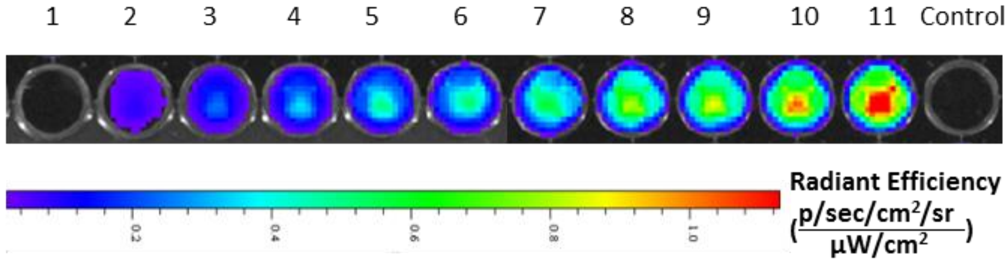

b

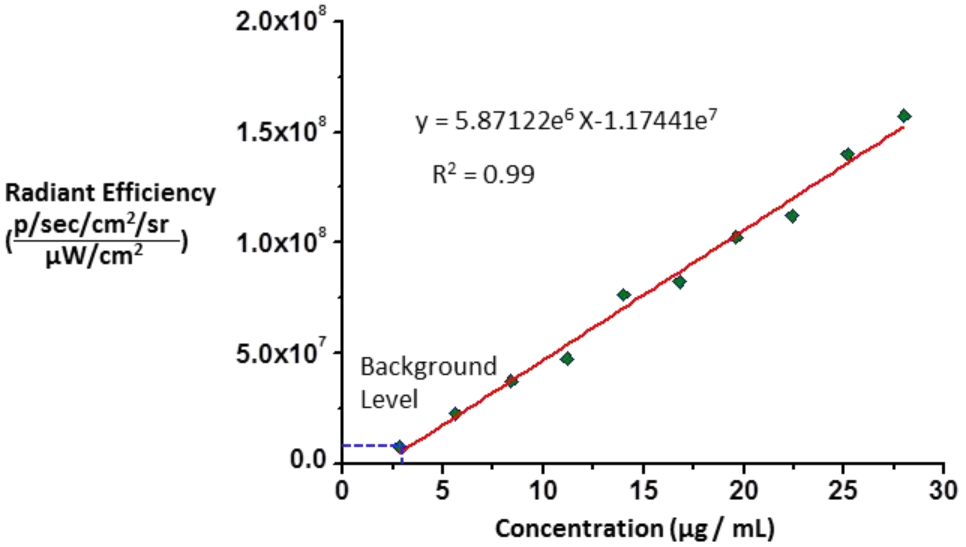

Supplementary Figure 35 | Quantitative analysis of the fluorescence intensity. In order to ensure the accuracy of the results, we added a standard solution in every tested 96-well plate. The fluorescence property of molecules **1**, **2** and **3** were identical. We take molecule **1** as example to illustrate the quantitative analysis. **a**. The top view of fluorescence images of a serial concentration of molecule **1** by Maestro II. The number from 1 to 11 represented 2.8, 5.6, 8.4, 11.2, 14.0, 16.8, 19.6, 22.4, 25.2, 28.0, 35.0 (μg/mL), respectively. The control group represented PBS buffer. **b**. Fluorescence intensity (relative to the background signal) increased linearly with the concentration (R^2 = 0.99).

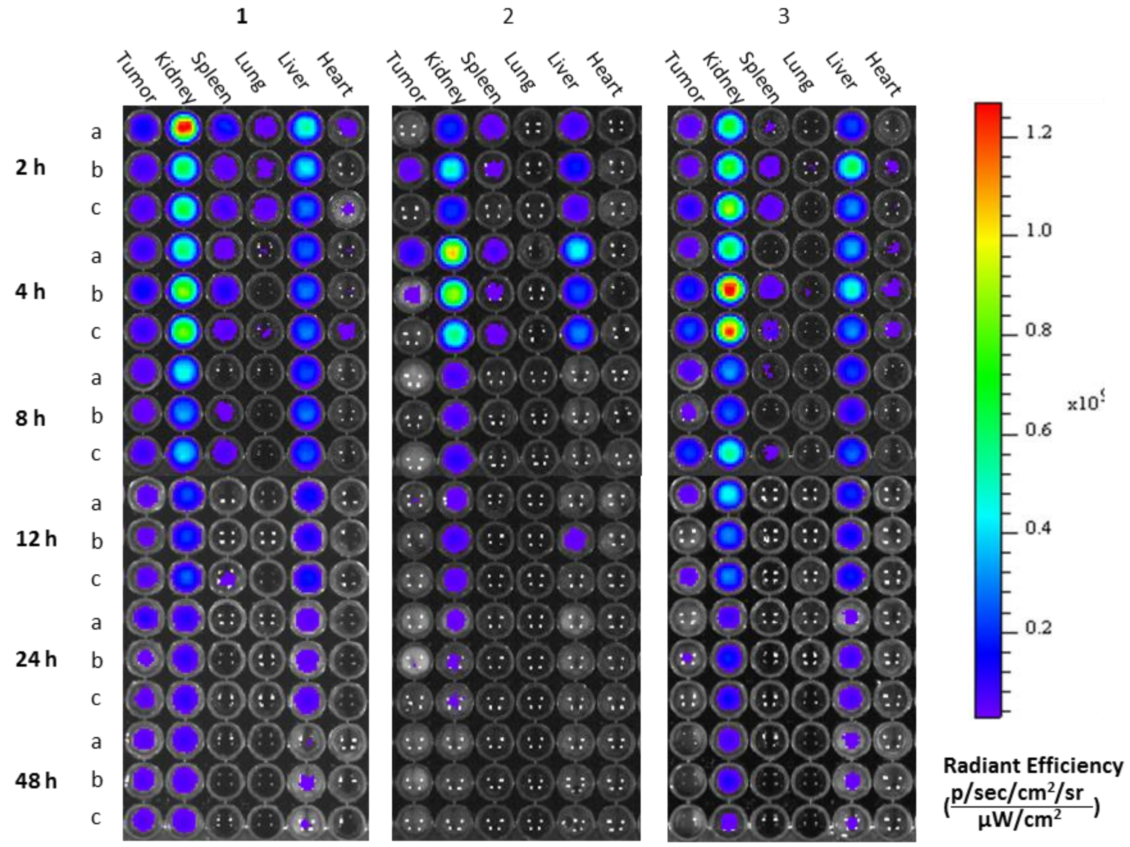

Supplementary Figure 36 | Representative NIR fluorescence images of tissue lysates. The molecules **1**, **2** and **3** (14 mg/kg, n = 3) on H460 tumour-bearing mice after intravenous administration in three groups. The mice were sacrificed at 2 h, 4 h, 8 h, 12 h, 24 h or 48 h post injection. All major organs (*i.e.*, heart, liver, spleen, lung, kidney) and tumour were collected, weighed, and homogenized (0.3 mL of lysis buffer per 100 mg of tissue). The homogenous tissue solutions were obtained for fluorescence imaging.

P8: AVPIAQKDDEEC

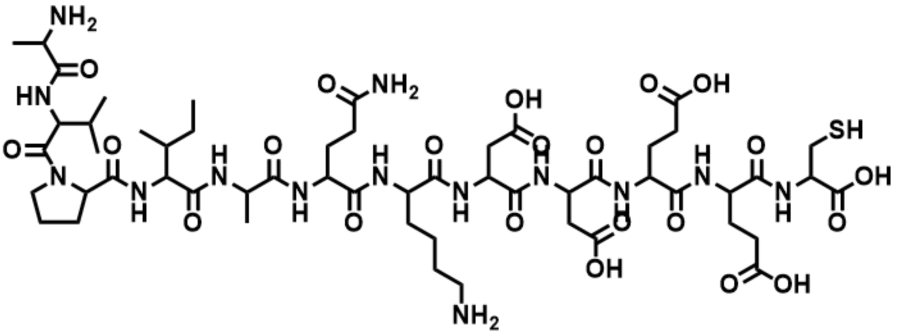

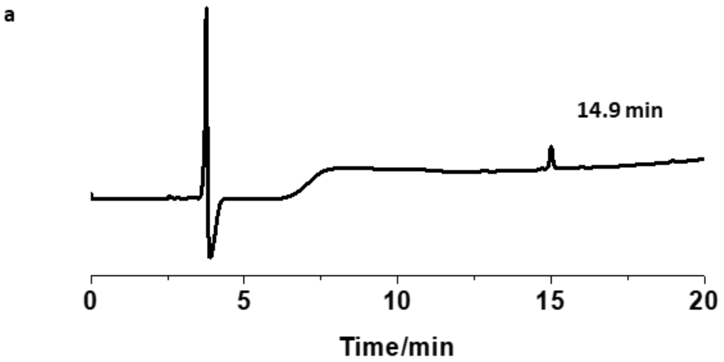

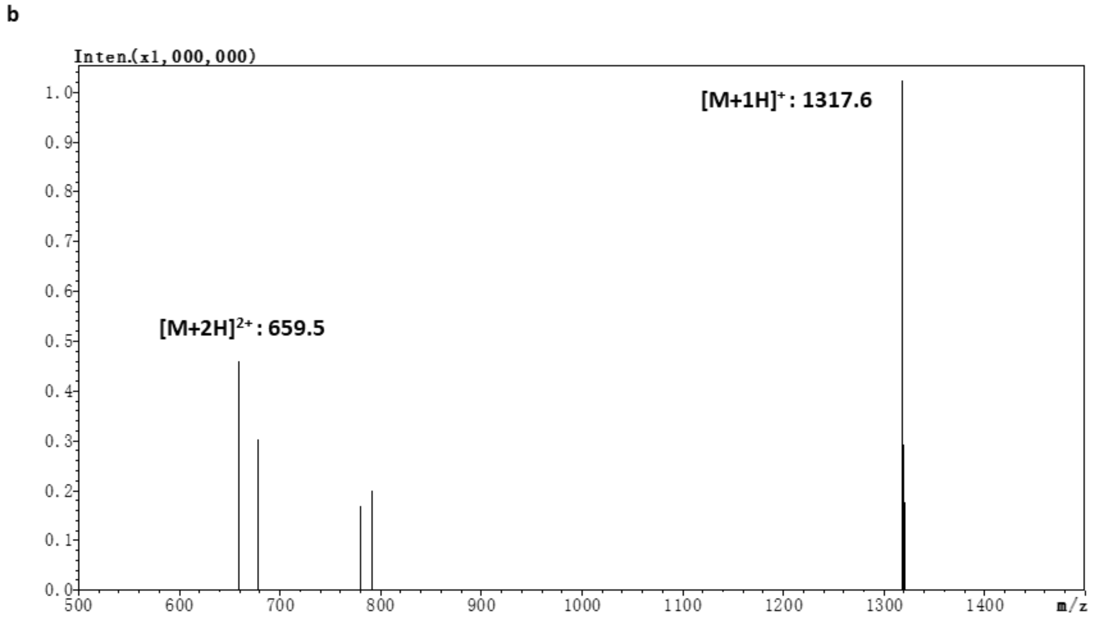

Supplementary Figure 37丨 HPLC spectra (**a**) and ESI-MS (**b**) of **P8**. The method of HPLC spectra was as follows: solvent A, 0.1% trifluoroacetic in 100% water; solvent B, 0.1% trifluoroacetic in 100% acetonitrile; 0.01 min, 10% solvent B, 5 min, 30% solvent B; 20 min, 80% solvent B

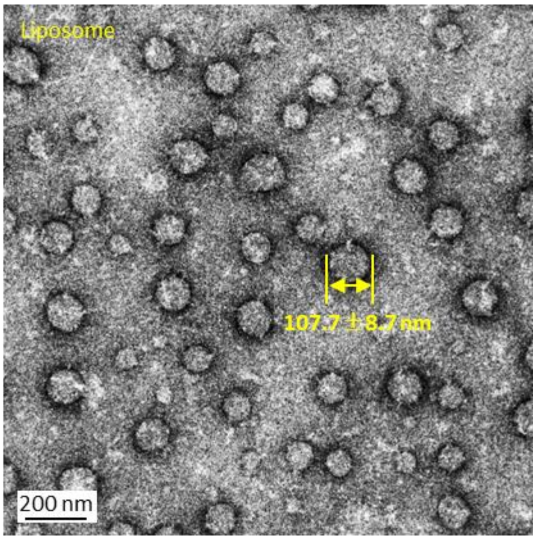

Supplementary Figure 38｜ TEM images of liposomes.

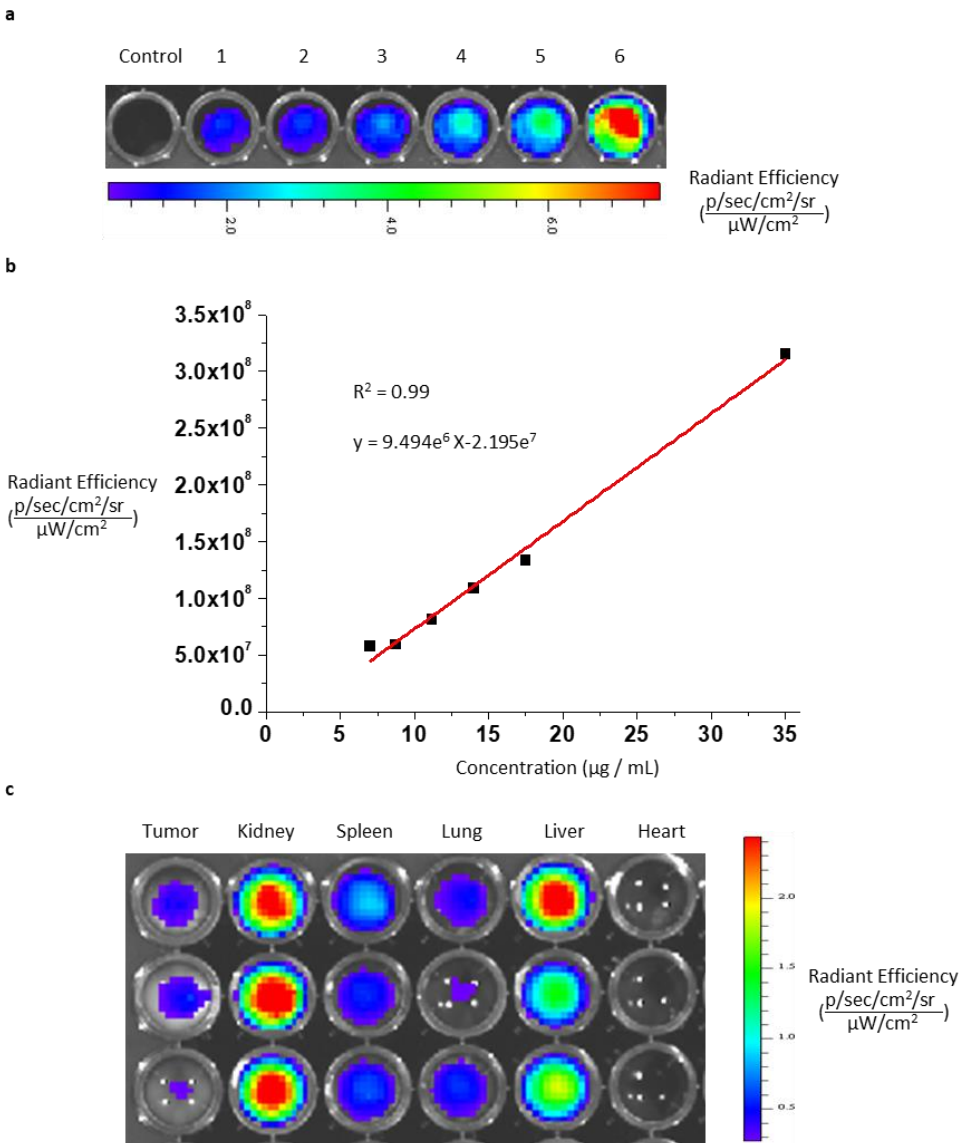

Supplementary Figure 39 | Quantitative analysis of the fluorescence intensity. In order to ensure the accuracy of the results, we added a standard solution in every tested 96-well plate. a. The top view of fluorescence images of a serial's concentration of Pep-nanofibers by Maestro II. The number from 1 to 6 represented 7.0, 8.7, 11.2, 14.0, 17.5, 35.0 (μg/mL), respectively. The control group represented PBS buffer. b. Fluorescence intensity (relative to the background signal) increased linearly with the concentration (R^2 = 0.99). c. The Pep-nanofibers (14 mg/kg, n = 3) on H460-tumour-bearing mice after intravenous administration in three groups. The mice were sacrificed at 48 h post injection. All major organs (i.e., heart, liver, spleen, lung, kidney) and tumour were collected, weighed, and homogenized (0.3 mL of lysis buffer per 100 mg of tissue). The homogenous tissue solutions were obtained for fluorescence imaging.

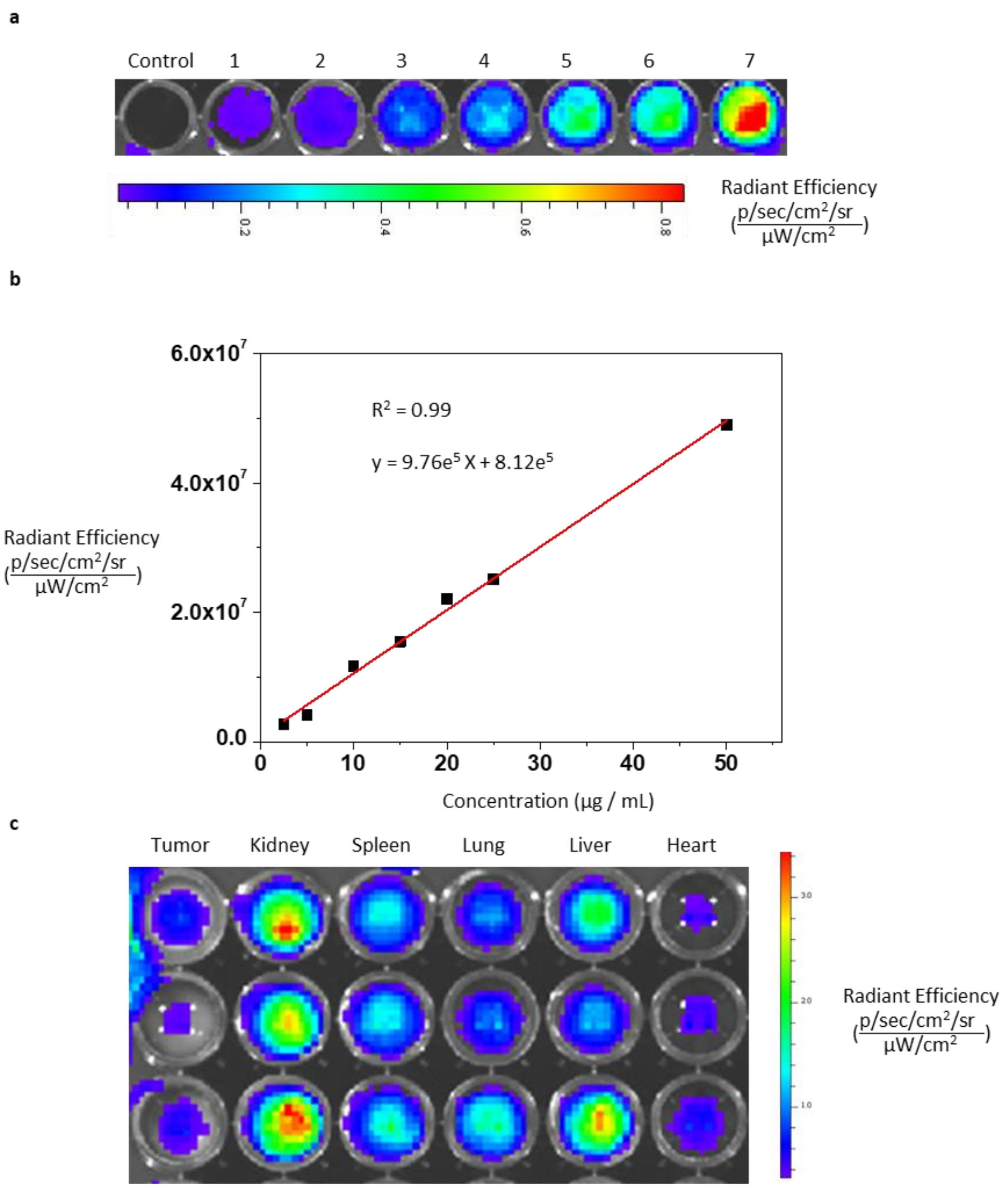

Supplementary Figure 40 | Quantitative analysis of the fluorescence intensity. In order to ensure the accuracy of the results, we added a standard solution in every tested 96-well plate. a. The top view of fluorescence images of a serial's concentration of Liposome by Maestro II. The number from 1 to 7 represented 2.5, 5.0, 10.0, 15.0, 20.0, 25.0, 50.0 (μg/mL), respectively. The control group represented PBS buffer. b. Fluorescence intensity (relative to the background signal) increased linearly with the concentration (R^2 = 0.99). c. The Liposome (14 mg/kg, n = 3) on H460-tumour-bearing mice after intravenous administration in three groups. The mice were sacrificed at 48 h post injection. All major organs (i.e., heart, liver, spleen, lung, kidney) and tumour were collected, weighed, and homogenized (0.3 mL of lysis buffer per 100 mg of tissue). The homogenous tissue solutions were obtained for fluorescence imaging.

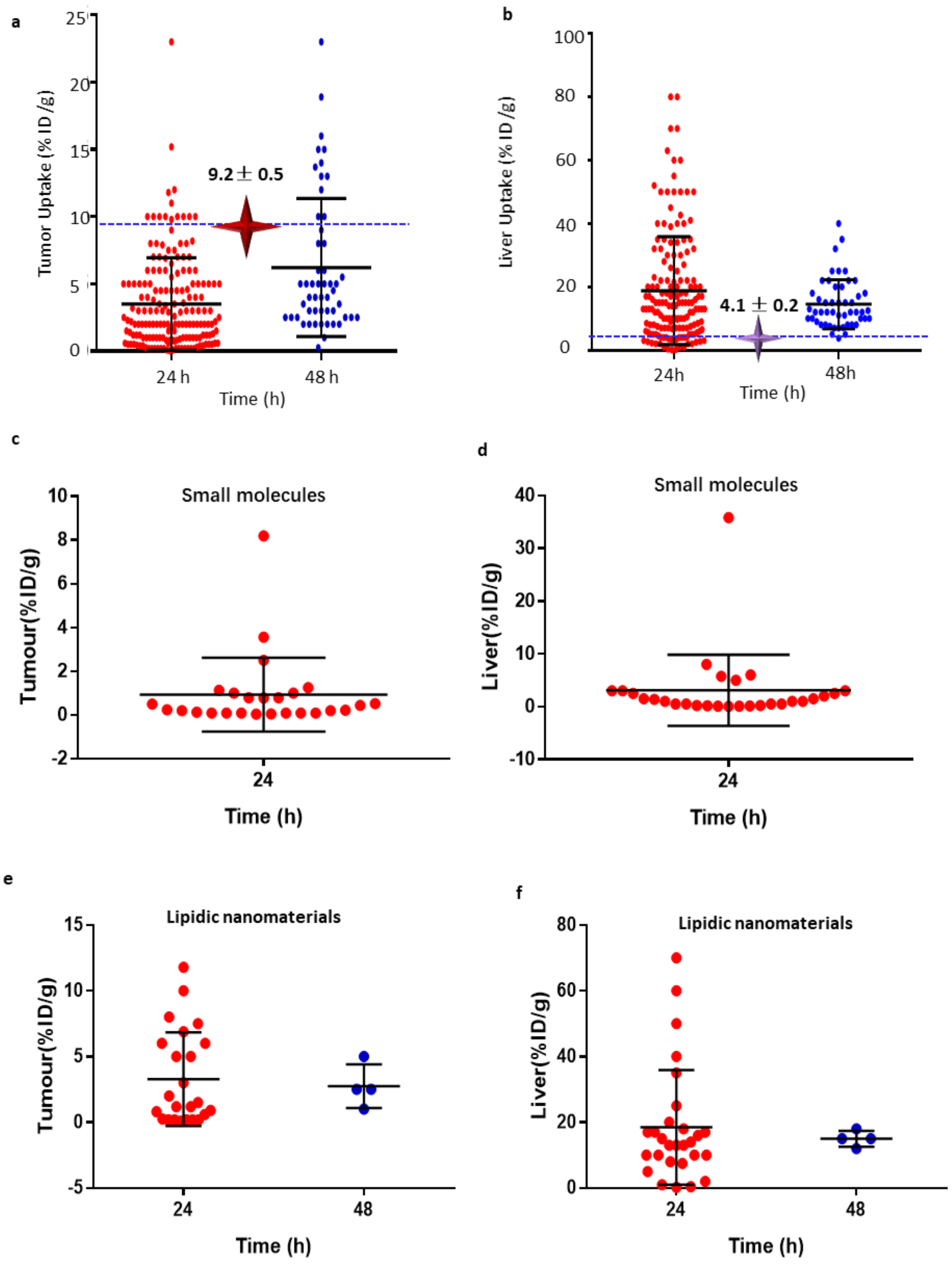

Supplementary Figure 41 | Statistical analysis of the tumour accumulation and liver uptake of organic and inorganic nanomaterials with various parameters (size, shape, surface chemistry, *etc.*) and functions (actively targeting, high penetration, long-circulation, *etc.*) (a and b), small molecule (c and d) and lipidic nanomaterials (e and f) from 120 literatures over the past 10 years. The red star represents the tumour uptake of molecule 1 (9.2±0.5 % ID/g 48 h) by standard quantitative analysis in tissue homogenate in a. The purple star represents the liver uptake of molecule 1 (4.1±0.2 % ID/g 48 h) by standard quantitative analysis in tissue homogenate in b.

Supplementary Table 1 | Pharmacokinetic parameters of intravenously-administered 1, 2 and 3 in tumour.

Pharmacokinetic parameters	1	2	3
$AUC_{0\to t}$ (mg/g*h)	71.2±4.7	12.2±1.9	26.6±8.9
$AUC_{0\to\infty}$ (mg/g*h)	145.9±1.0	14.1±2.2	34.6±12.2
$t_{1/2\beta}$ (h)	69.3±0	10.0±1.9	10.6±2.8
CL (L/h/kg)	0.078±0.03	1.0±0.15	0.44±0.15
Cmax (mg/L)	2.0±0.04	0.8±0.3	1.4±0.5
Tmax (h)	4.0±0	3.3±1.2	6.7±2.3
$MRT_{(0-t)}$ (h)	23.4±0.2	15.2±4.5	10.7±1.8

Data are presented as means ± SD (n = 3).

$t_{1/2\beta}$, elimination half-life; CL, plasma clearance ; Cmax, maximum concentration ; Tmax, peak time； MRT: mean retention time ; AUC(0-t), area under the 1, 2 and 3 concentration-time curve from time zero to the last quantifiable value; AUC(0-∞), area under the 1, 2 and 3 concentration-time curve from time zero to infinity, which represents the total drug exposure over time. h, hour(s).

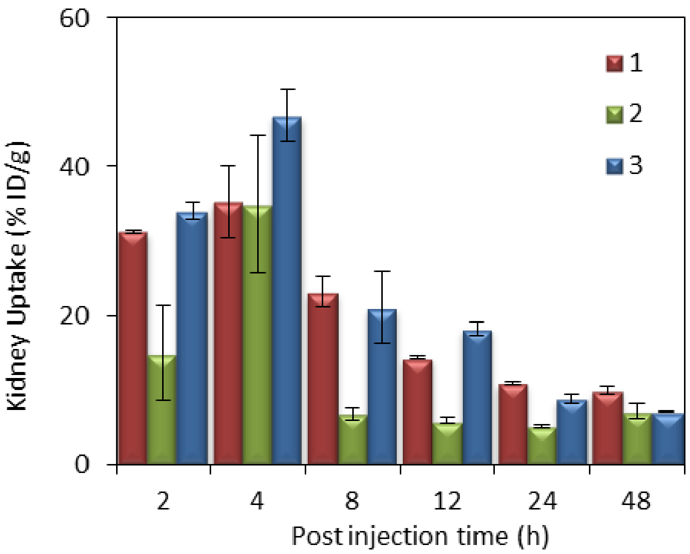

Supplementary Figure 42 | Kidney uptake of molecules **1**, **2** and **3** (14 mg/kg, n = 3) in H460 tumour-bearing nude mice at 2 h, 4 h, 8 h, 12 h, 24 h and 48 h after injection (%ID g^{-1} = percentage of the injected dose per gram of tissue). Data are mean ± s.d. (n = 3).

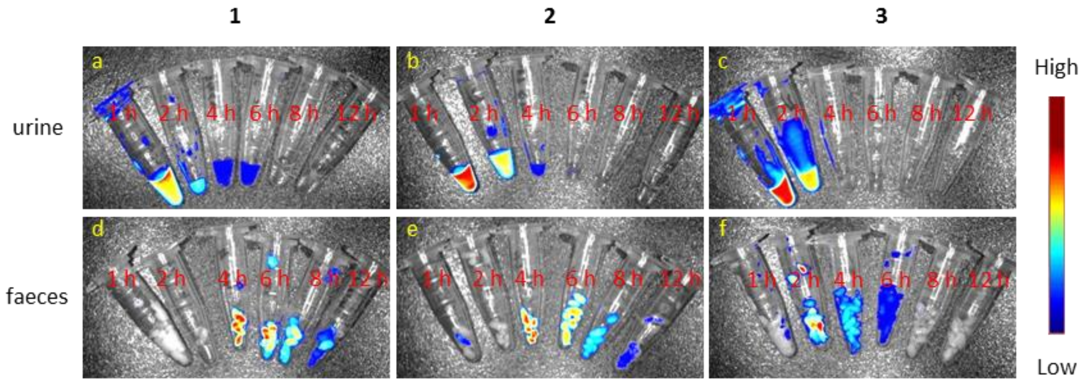

Supplementary Figure 43 | NIR fluorescence imaging of urine and faeces at different time points. a, b and c represented the imaging of urine of H460 tumour-bearing mice injected with molecules **1**, **2** and **3**, respectively. d, e and f represented the imaging of faeces of H460 tumour-bearing mice injected with molecules **1**, **2** and **3**, respectively. H460 tumour-bearing mice were intravenously injected with molecules **1**, **2** and **3** (14 mg/kg, n = 3). Urine and faeces were collected at 1, 2, 4, 6, 8 and 12 h without coercion.

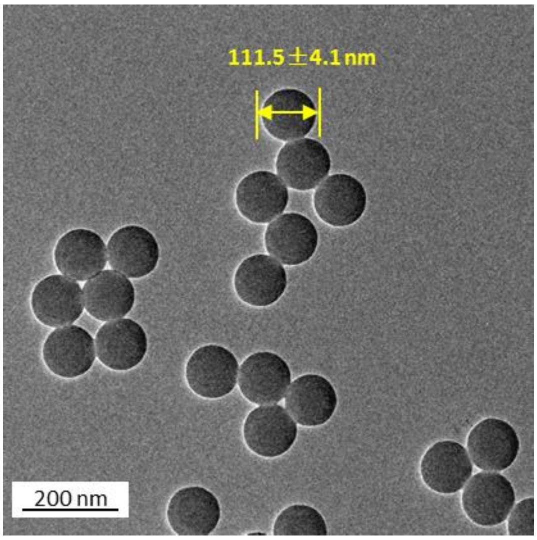

Supplementary Figure 44 | TEM images of SiO_2 NPs.

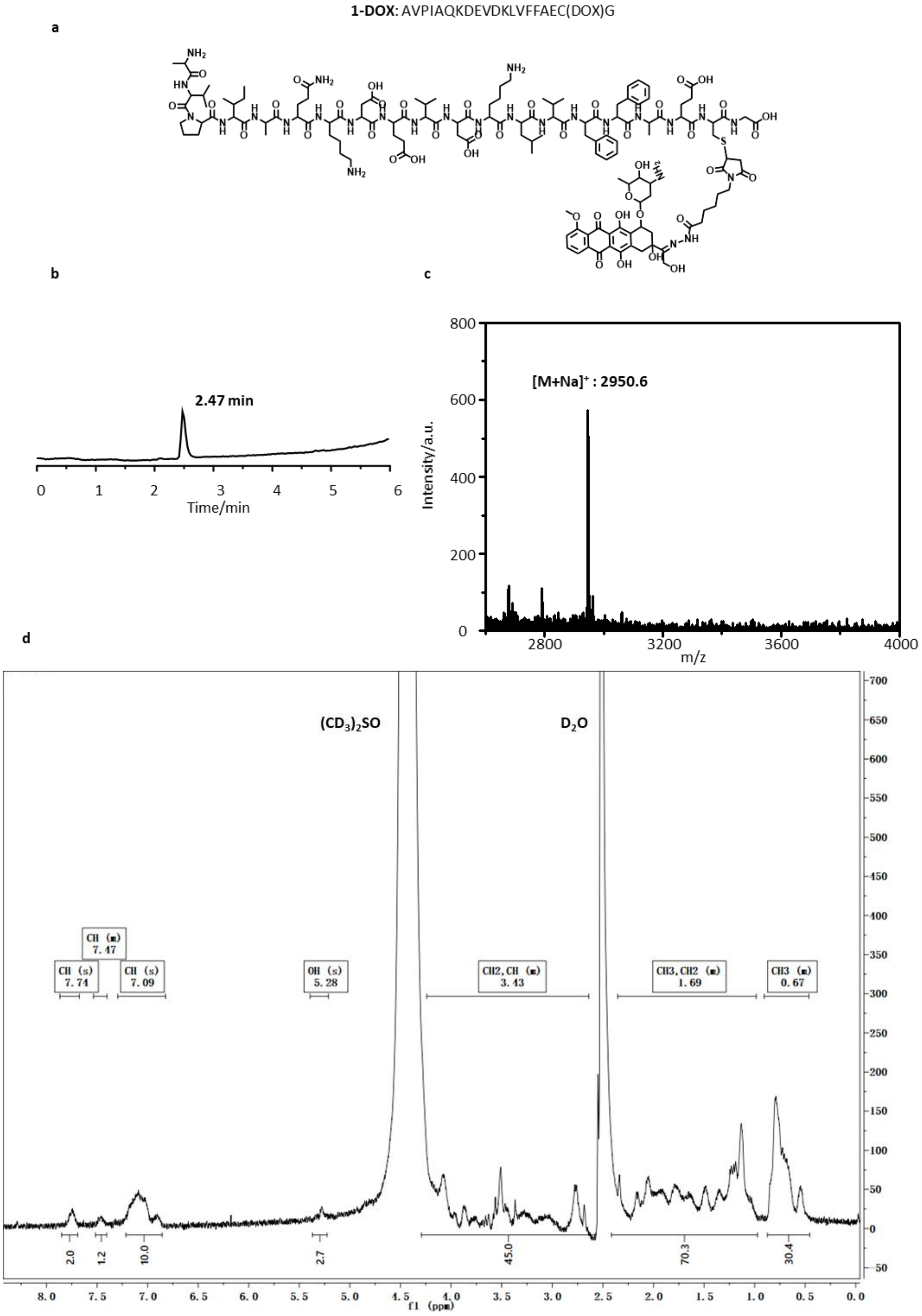

Supplementary Figure 45 | a The structure of molecule **1-DOX**; UPLC spectra (**b**) and MALDI-TOF (**c**) molecule **1-DOX**; The method of UPLC spectra was as follows: solvent A, 100% water; solvent B, 100% acetonitrile; 0.01 min, 30% solvent B, 5 min, 90% solvent B, 5.01 min, 100% solvent B, 6 min, 100% solvent B. **d**, ^{1}H NMR of molecule **1-DOX** in D_2O and DMSO-*d6*. ^{1}H NMR (400 MHz, D_2O, DMSO-*d6*) δ 7.74 (s, 2H, CH), 7.47 (s, 1H, CH), 7.29-6.82 (m, 10H, CH), 5.28 (s, 3H, OH), 4.24-2.64 (m, 45H, CH_2, CH), 2.35-0.96 (m, 70H, CH_3, CH_2), 0.91-0.46 (m, 30H, CH_3).

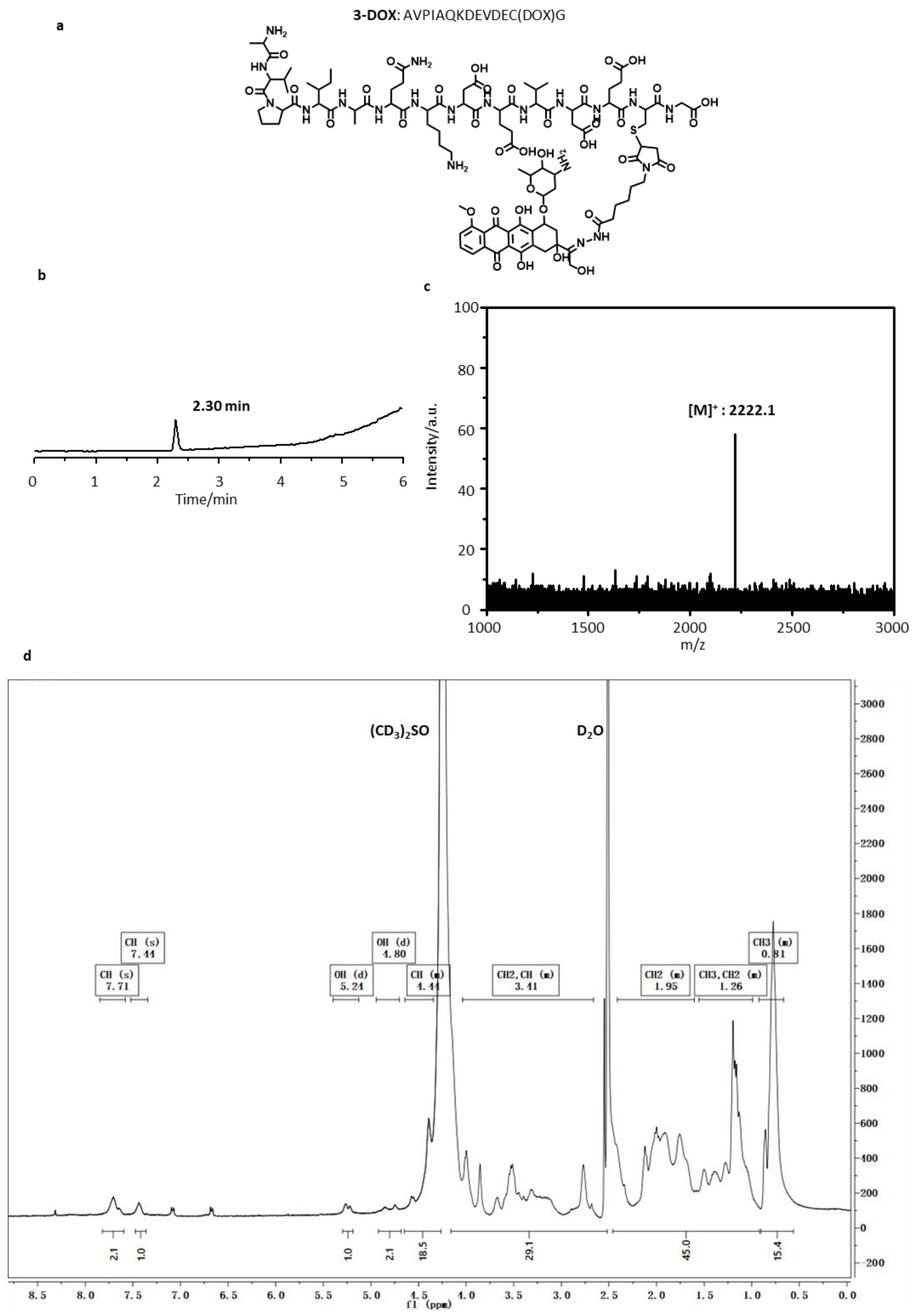

Supplementary Figure 46 | **a** The structure of molecule **3-DOX**; UPLC spectra (**b**) and MALDI-TOF (**c**) molecule **3-DOX**; The method of UPLC spectra was as follows: solvent A, 100% water; solvent B, 100% acetonitrile; 0.01 min, 30% solvent B, 5 min, 90% solvent B, 5.01 min, 100% solvent B, 6 min, 100% solvent B. **d**, ^{1}H NMR of molecule **3-DOX** in in D_2O and DMSO-*d6*. ^{1}H NMR (400 MHz, D_2O, DMSO-*d6*) δ 7.71 (s, 2H, CH), 5.24 (d, 1H, OH), 4.80 (d, 2H, OH), 4.64-4.34 (m, 19H, CH), 4.04-2.66 (m, 29H, CH_2, CH), 4.24-2.64 (m, 45H, CH_2, CH), 2.41-0.99 (m, 45H, CH_3, CH_2), 0.92-0.66 (m, 15H, CH_3).

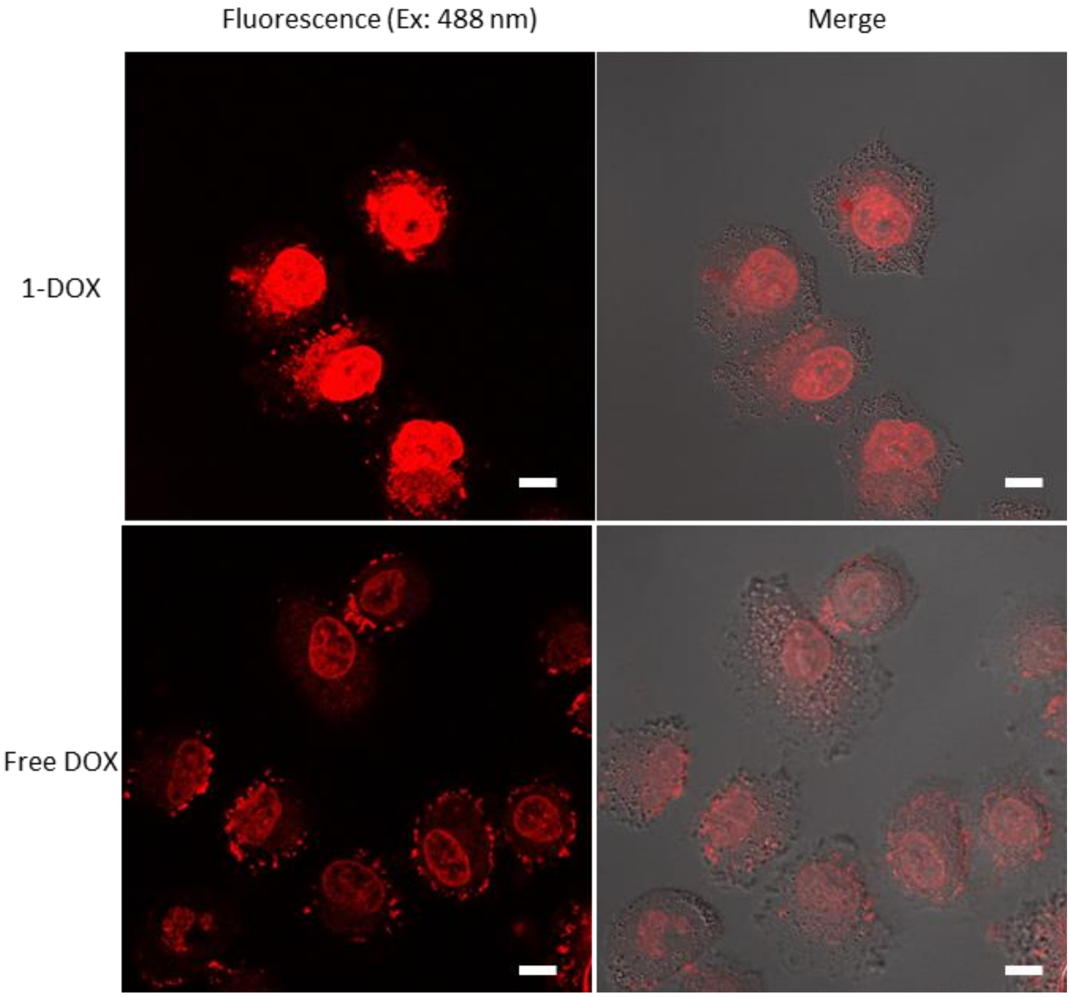

Supplementary Figure 47 | The confocal imaging of H460 cells treated with molecule 1-DOX (0.1 μM) and free DOX (0.1 μM) for 1 h at 37 ^{o}C. Scale bar, 10 μM.

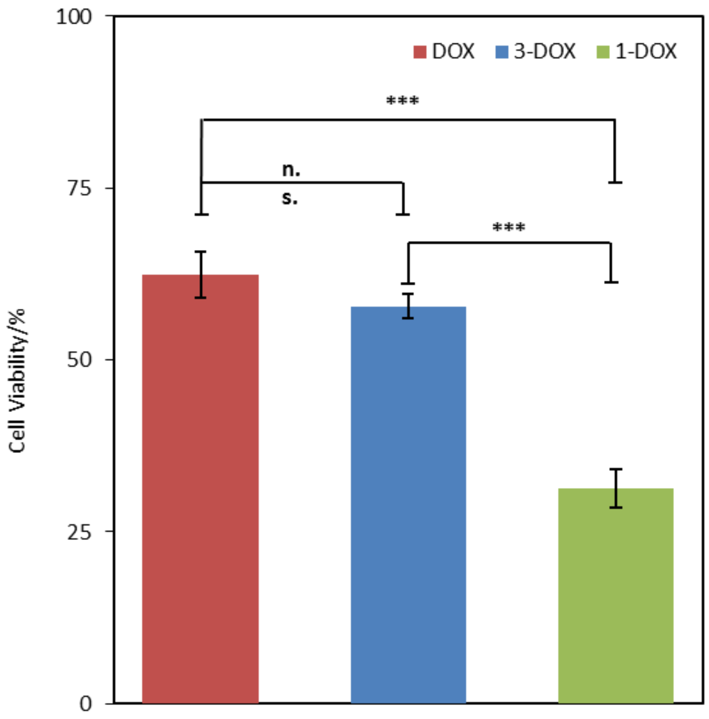

Supplementary Figure 48 | The cytotoxicity assay: tumour cells (H460) were treated with the molecule 1-DOX (100 nM), free DOX (100 nM) and the molecule 3-DOX (100 nM). The sample solutions were added to each well, and the cells were incubated for additional 24 h. Then each well was added with 10 μL of CCK-8 solutions and cultured for another 4 h. Microplate reader was used to measure the UV-Vis absorptions of sample wells (A_{sample}), A_{black} and control wells ($A_{control}$) at a test wavelength of 450 nm and a reference wavelength of 690 nm, respectively. Cell viability (%) was equal to (A_{sample}-A_{blank})/ ($A_{control}$-A_{blank}) x 100. All the experiments were performed in quadruplicate. Data are presented as the mean ± s.d. (n = 4). *** $p < 0.001$, p values were performed with one-way ANOVA followed by post hoc Tukey's test for the indicated comparison. n. s. represents no significance.

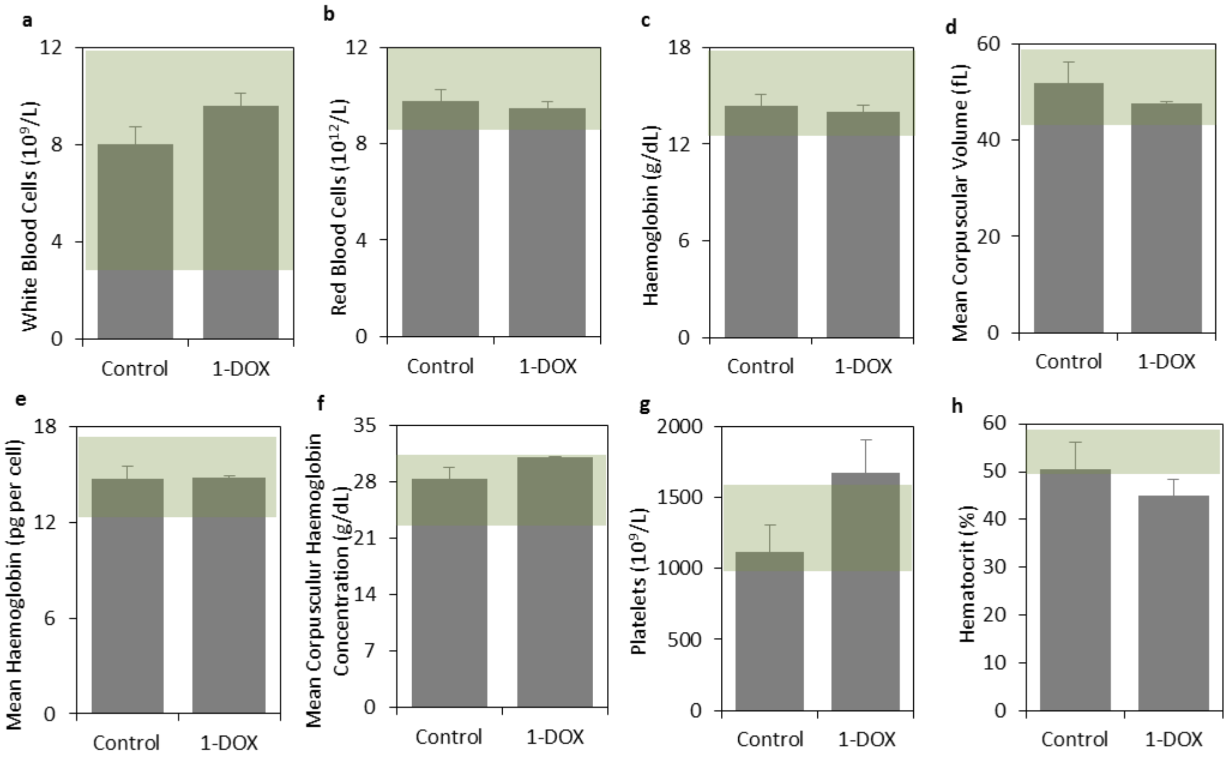

Supplementary Figure 49 | Toxicology evaluation of the tumour therapy. The changes of hematology data, including a) white blood cells, b) red blood cells, c) haemoglobin, d) mean corpuscular volume, e) mean corpuscular haemoglobin, f) mean corpuscular haemoglobin concentration, g) platelets and h) haematocrit after treated with molecule 1-DOX (an identical DOX dose of 2.0 mg/kg) treatment comparing with normal range (green backgrounds). Data are presented as mean ± s.d. (n=5).

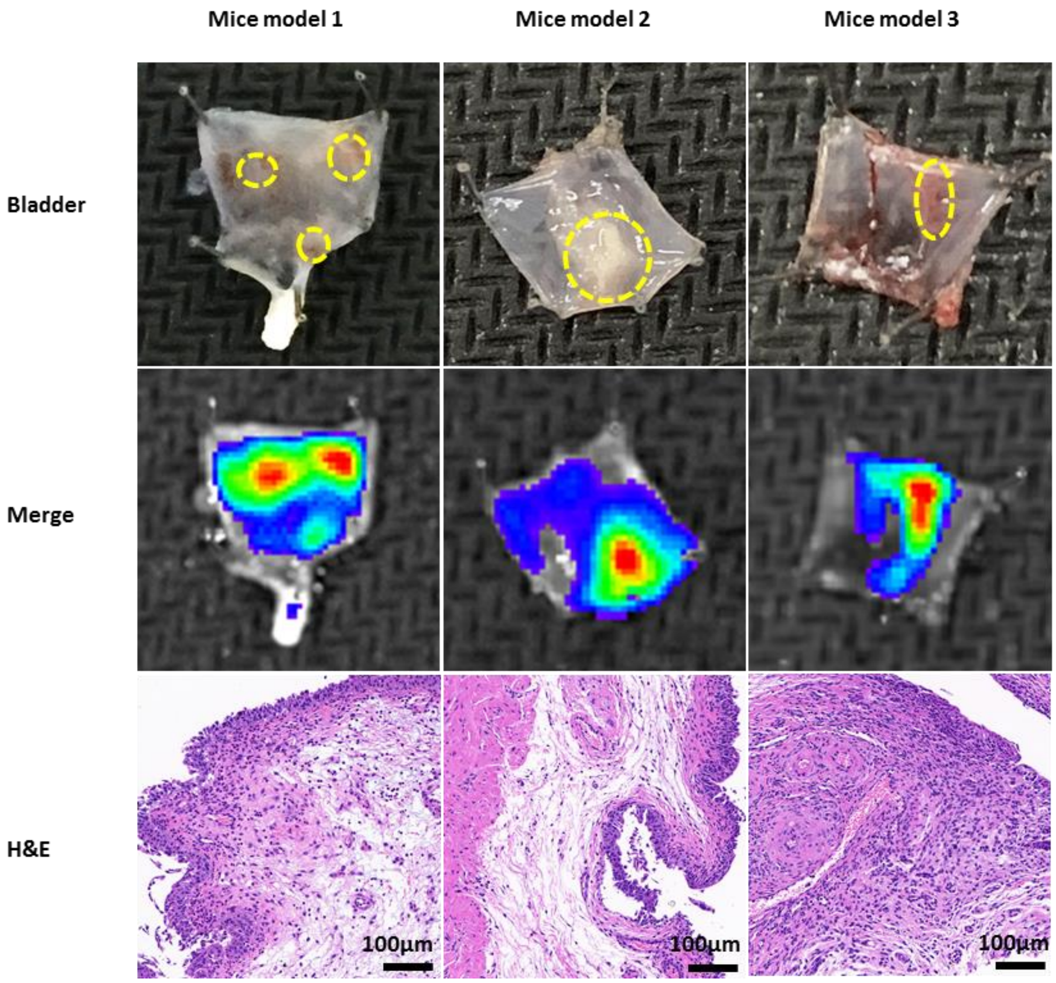

Supplementary Figure 50 | The fluorescence images of the ex-bladder in orthotopic bladder tumour mice. The mice were treated with 100 uL molecule 1 (50 μM) in PBS for 1 h through intravesical instillation. Histology evaluations of the tissues were collected from the signal site in the ex-bladder.

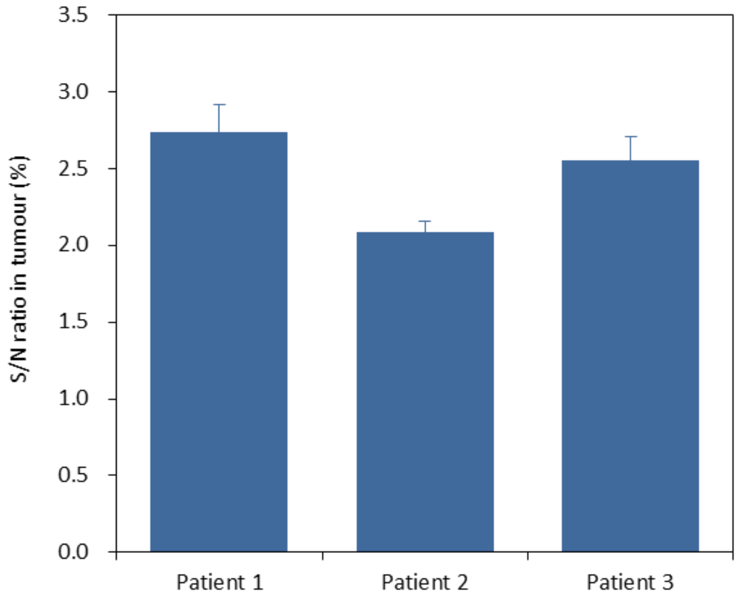

Supplementary Figure 51 | The S/N ratio of NIR fluorescence in tumour and its surrounding tissues in Fig.6b.

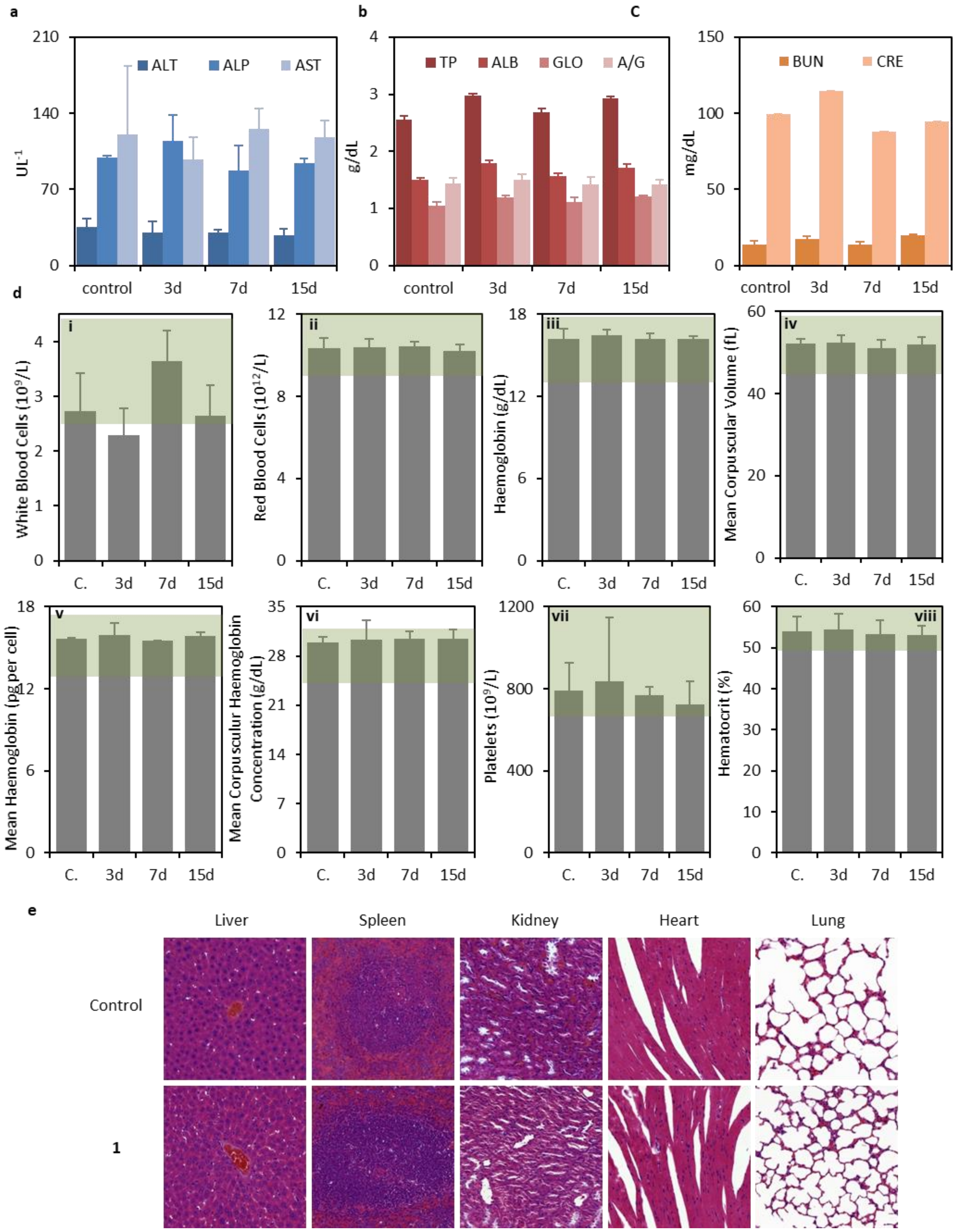

Supplementary Figure 52 | Toxicology evaluation of the TCASS. Blood biochemistry and hematology data of the healthy female Balb/c nude mice which were treated with molecule 1 (14

mg/kg, n=3) at 3, 7 and 15 d. The control group was healthy mice group without treatment. a, As major indicators of liver function, alanine aminotransferase (ALT), alkaline phosphatase (ALP) and aspartate aminotransferase (AST) levels were evaluated at different time points. b, Total Protein (TP), albumin (ALB) and globulin (GLO) concentration and A/G (ALB/GLO) levels were evaluated at different time points. c, The creatinine (CRE) and blood urea nitrogen (BUN) levels, important indicators of kidney function, were evaluated at different time points. d, Time-course changes of haematology data, including i) white blood cells, ii) red blood cells, iii) haemoglobin, iv) mean corpuscular volume, v) mean corpuscular haemoglobin, vi) mean corpuscular haemoglobin concentration, vii) platelets and viii) haematocrit after treated with molecule 1 (14 mg/kg) treatment comparing with normal range (green backgrounds). Data are presented as mean ± s.d. (n=3). e, Histology evaluation of the major organs (liver, spleen, kidney, heart and lung) were collected from the control group without treatment and molecule 1 treated (12 h post injection) group. Scale bars, 100 µm.

Supplementary Reference

1. An, H. W., Qiao, S. L., Hou, C. Y. *et al.* Self-assembled NIR nanovesicles for long-term photoacoustic imaging in vivo. *Chem. Commun.* **51**, 13488-13491 (2015).
2. Chen, X., Peng, X., Cui, A. *et al.* Photostabilities of novel heptamethine 3H-indolenine cyanine dyes with different N-substituents. *J. of Photochem. and Photobio. A: Chemistry* **181**, 79-85 (2006).
3. Frens, G. Controlled nucleation for regulation of particle size in monodisperse gold suspensions. *Nat. Phys. Sci.* **241**, 20-22 (1973).
