# Supplementary material for: A tumour-selective cascade activatable self-detained system for drug delivery and cancer imaging
Source: Nat Commun. 2019 Oct 24;10:4861. doi: 10.1038/s41467-019-12848-5 (PMC6813295; doi:10.1038/s41467-019-12848-5)
Supplement: Supplementary file 2 — Description of Additional Supplementary Files [file 41467_2019_12848_MOESM2_ESM.pdf]

Description of Additional Supplementary Files

File Name: Supplementary Movie 1
Description: The imaging of fresh isolated intact bladder from patients after intravesical instilled with molecule 1.
